# Supplementary material for: A novel ilarvirus protein CP-RT is expressed via stop codon readthrough and suppresses RDR6-dependent RNA silencing
Source: PLoS Pathog. 2024 May 30;20(5):e1012034. doi: 10.1371/journal.ppat.1012034 (PMC11166343; doi:10.1371/journal.ppat.1012034)
Supplement: S1 Data — (PDF) [file ppat.1012034.s001.pdf]

A novel ilarvirus protein CP-RT is expressed via stop codon readthrough and suppresses RDR6-dependent RNA silencing

Nina Lukhovitskaya[1*], Katherine Brown[1], Lei Hua[2], Adrienne E. Pate[2], John P. Carr[2] and Andrew E. Firth[1*]

Correspondence: nl358@cam.ac.uk (NL), aef24@cam.ac.uk (AEF)

[1]Department of Pathology, University of Cambridge, United Kingdom

[2]Department of Plant Sciences, University of Cambridge, United Kingdom

Supplementary Material

Supplementary Table A. Readthrough statistics for the different clusters. Columns 1 and 2 show the cluster designation and the accession number of the reference sequence for the cluster, respectively. Column 3 indicates the apparent presence (Y) or absence (N) of a lengthy insert in RNA3 when the 3′-of-CP region was compared with the 3′UTRs of RNAs 1 and 2; we take presence of a lengthy insert as a proxy for the putative presence of a readthrough domain in that cluster; a "U" for clusters 26 and 32 indicates "unknown" due to the absence of appropriate RNA1/RNA2 sequence data. Column 4 shows the median length across the cluster of the ORF in-frame with and immediately 3′-adjacent to the CP stop codon, whether or not readthrough is hypothesised to occur in that cluster; ORFs that are incomplete as a result of running off the 3′ end of the available RNA3 nucleotide sequence are excluded from the median calculation. Columns 5 and 6 show the identity of the CP stop codon, and the six 3′-adjacent nucleotides in the reference sequence (which is generally typical for the cluster, but see Supplementary Dataset C for data for all sequences in each cluster). Clusters are presented in descending order by column 4.

Cluster	Reference	Insert	Length	CP stop	Context
**5a**	NC_006568	Y	111	UAG	CAAUUA
**5b**	MN527504	Y	84	UAG	CAGUUA
**35**	NC_038776	Y	60	UGA	CCACAC
**34**	OL964098	Y	57	UGA	CCACAC
**12**	NC_005854	Y	57	UGA	AUGGUU
**4**	NC_011555	Y	49	UGA	CUGGUU
**26**	MN386958	U	49	UGA	AUCGCG
**8b**	NC_003570	Y	48	UGA	CUACAC
**30**	NC_022129	Y	48	UGA	CUCACU
**29**	NC_027930	Y	48	UGA	CUAGAU
**14**	NC_011807	Y	48	UGA	CCACGC
**37**	NC_040437	Y	46	UGA	CUACAC
**36**	MG800791	Y	46	UGA	AAGGUU
**11**	NC_008706	Y	46	UGA	CUGUCU
**1**	NC_003845	Y	45	UGA	CUAGAU
**10**	OL472062	Y	45	UGA	CGGUCA
**25**	OL539725	Y	44	UGA	AGGUCA
**20**	OM323991	Y	44	UGA	AGGUCC
**16**	OL584350	Y	44	UGA	CUACUU
**15**	NC_040394	Y	44	UGA	CUAUCU
**8a**	NC_009536	Y	43	UGA	CUACAC
**27**	MZ170698	Y	42	UGA	CUACAC
**33**	OL472059	Y	41	UGA	CCACGC
**17a**	NC_003835	Y	41	UGA	CUACAC
**17b**	NC_039076	Y	39	UGA	CCACGC
**13**	NC_003810	Y	39	UGA	CUACAC
**3**	NC_008038	Y	38	UGA	CUAUAU
**23**	KX196164	Y	38	UGA	CUAUAU
**18**	NC_003546	Y	37	UGA	CCACAA
**21**	MW962375	N	35	UAG	GUUGGG
**6b**	HG328285	Y	30	UAG	CAAUCA
**32**	JN107639	U	≥23	UGA	CUAUUU
**2**	NC_004364	N	23	UAG	AGUGAG
**22**	ON932436	N	19	UGA	CUCUAC
**7**	NC_040470	N	9	UAG	GUUGAG
**28**	KU947035	N	9	UGA	GAACCG
**6a**	NC_003480	N	3	UGA	UGGAGC
**9**	NC_022252	N	1	UAG	AAGUGA
**31**	NC_025481	N	1	UAG	GUAUGA
**24**	NC_006066	N	1	UAA	AUCUAG
**19**	NC_003453	N	0	UAA	UGAAAA

Supplementary Table B. Application of HHpred to the putative RT sequence. For each cluster with a putative RT domain (column 1) the amino acid sequence of the reference sequence RT domain (column 2) was submitted to HHpred [1] using the subject databases PDB_mmCIF70_18Jun [2] and Pfam-A_v36 [3]. Many queries returned large numbers of "hits"; the top hit by HHpred "Probability" score is reported in columns 3–5 and the region of the top hit is indicated with blue text and underlining in column 2. HHpred e-values below 1.0 are highlighted in bold red text. Sequences are ordered according to the CP phylogenetic tree (Supplementary Figure B). In the case of NC_008706 (cluster 11), the RT sequence is shown after insertion of the frame-correcting "A" nucleotide (see Supplementary Dataset C for details). All cysteine and histidine residues are highlighted.

Cluster	HHpred match on RT sequence	PDB/Pfam top hit	HHpred Probability score	HHpred e-value score
37	LHSGDYLSELEYDPMSGWFLNRGVRWYYCPIPACVFTNGYRLGEDH	PF17780.5	74.41	10
6b	QSGSQISNIVRIFDNLDDLMERFAPYAEGA	1GW4_A	74.85	5.6
5a	QLGRDISRLRDVSRRAKVPNDFIVGCEPAYDIDMCEQAHDGYPEVNPAVLRPRFFSVPRTVRRSRRLNPRLVDEANLPYFTGECVRCGYTPKSLIDQREWECSSCYMLYAA	1SPW_A	89.33	**0.25**
5b	QLGRVNTQFRVLARRVEIPGIDPRCEFDAQGSLTFRSRSVPRVFNRPRRLNPRLSKEEEEALDHFDQVDNSDGADYLDDWRSYVD	PF14584.10	58.39	13
27	LHSGGVNFDTWNGICSRCGFKPKRLVDQYTYTCRECGKIYFA	PF09297.15	97.7	**0.000037**
3	LYDPSFDCASTMSIPRNIRSWKCCFCNRIHHSEFVTEC	2NUT_A	91.71	**0.089**
23	LYDPFFDCSDSANFSFLPPEEMEFKAWRCCYCNEIHYV	PF14803.10	87.6	**0.49**
34	PHSGGRCLVCEVSWGLQTYTRIDDFSLRDECLYCGADAEIGLLCPSFKCPQGCTQCL	PF04055.25	82.35	1.4
35	PHSGSRCLVCGTSWSLQTYTYLEDFSLRDECLLCGADAEIGLICPSFKCPLGCTQYPCFG	4QIW_W	87.27	**0.67**
33	PRSGCESCGYVPYCHCKCYNDACSVVGCEQNIWFIPDEVFP	PF14803.10	87.5	**0.48**
18	PQDCESCGYIPYCHCECWPYRCYTIGCNANDLWFIDS	PF14803.10	84.17	**0.88**
17a	LHSGFCPVCGYVPLCHCMCYNYSCDVIGCDANDTFCIEDDQV	PF04216.16	93.85	**0.042**
17b	PRSGLCQSCGFIPYCHCYCCDDVCDVIGCLANDTVYDEV	PF10868.12	89.53	**0.36**
13	LHSDDCSSRNVIPFCHCSCCPFHCDVIGCVCNDAFFYED	PF20365.2	91.6	**0.21**
14	PRSGECVYLHSNECEKKNVIPFCHCECYDNACNTLWCPANVFCFHEP	PF20365.2	48.19	15
8a	LHSDDCPSQSVIPFCHCECYDNACGTPWCPANANCFHEFHENY	PF21803.1	70.87	3
8b	LHSGDYLHSVECERKCVIPFCHCVCYDDACDVLWCSPNDLCYHESNDW	PF20365.2	42.99	13
25	RSTSSGSELPCIMNNSCARCGFRPPYVIDQYIWSCALCHYKHYA	2K4X_A	91.63	**0.024**
36	KVAASGVVPEMPHVDGNSCVRCGYTPKYLIDSYIWTCFMCSYEQYA	PF01907.23	80.52	**0.97**
12	MVVTDVSGVDANVKSDDSYQDGDYYYDTDMFQYDCTDDDDGTAYMQDFFSCVAAMNF	PF15601.10	29.07	64
20	RSQSSHDEMPHVKNNSCVRCGFRPPQIIDQYVWRCRRCYLEHAA	PF18547.5	95.09	**0.0042**
10	RSQVPSPRLELPYFTEKCVRCGFAPKYTVDQYIWKCHVCSYEHYA	2K4X_A	96.19	**0.0035**
30	LTADSSSTVVDELPHMDGNSCVRCGFTPKRIVDQYVWRCYLCDYPQMA	PF18547.5	94.53	**0.023**
15	LSDMPQVNELPYITNCCVRCGFKPRRVVDQLVWSCTFCLYKQRA	2CJS_C	88.5	**0.062**
4	LVNYLEENFEVPHILPTIVGSCVRCGFRPRRVPDARYLWCGMCKYLHYA	PF04216.16	86.83	**0.39**
11	LSPILEFDLELPHIKNNSCVRCGYHPMYVVDQHIWKCDVCHQRHYA	PF09297.15	96.26	**0.0056**
16	LLSDLDNVELPHMRSSCVRCGFCPKYAVDQLIWRCHLCGYPHSA	3PWF_B	93.88	**0.012**
1	LDGHLGETEMPHIDGNSCVRCGFTPRRIVDQYIWKCDICSYEQYA	2K4X_A	95.41	**0.012**
29	LDGLVTSGRVSDLPHIDGNSCVRCGYAPRYIVDQCYWECTLCSYEQRA	PF11781.12	91.44	**0.27**

Supplementary Table C. Host and virus read counts for Ribo-Seq samples.

virus	read length	total reads	host rRNA	host mRNA	vRNA(+)
AV2	all	48,320,160	19,007,036	8,818,112	177,767
	27 nt	5,566,016	1,264,450	2,240,342	35,011
	28 nt	4,654,014	1,233,414	1,692,623	25,832
AV2-2st	all	50,912,256	22,067,441	8,727,297	4,598
	27 nt	6,342,426	1,636,497	2,480,632	1,053
	28 t	5,335,007	1,544,451	1,856,318	849

Supplementary Table D. Raw read counts for the siRNA sequencing experiment.

Virus	Timepoint	Replicate	Source	Strand	Read length (nt)	Raw read count
mock	7 dpi	1	virus	+	21	276
mock	7 dpi	1	virus	−	21	273
mock	7 dpi	1	host	+	21	1055407
mock	7 dpi	1	host	−	21	538183
mock	7 dpi	1	unmapped	.	21	115730
mock	7 dpi	1	virus	+	22	163
mock	7 dpi	1	virus	−	22	193
mock	7 dpi	1	host	+	22	1205193
mock	7 dpi	1	host	−	22	628443
mock	7 dpi	1	unmapped	.	22	139983
mock	7 dpi	1	virus	+	24	22
mock	7 dpi	1	virus	−	24	39
mock	7 dpi	1	host	+	24	5431754
mock	7 dpi	1	host	−	24	3175028
mock	7 dpi	1	unmapped	.	24	627142
mock	7 dpi	1	virus	+	all	633
mock	7 dpi	1	virus	−	all	683
mock	7 dpi	1	host	+	all	11436024
mock	7 dpi	1	host	−	all	6331472
mock	7 dpi	1	unmapped	.	all	1881361
mock	7 dpi	2	virus	+	21	438
mock	7 dpi	2	virus	−	21	373
mock	7 dpi	2	host	+	21	821755
mock	7 dpi	2	host	−	21	417490
mock	7 dpi	2	unmapped	.	21	111977
mock	7 dpi	2	virus	+	22	299
mock	7 dpi	2	virus	−	22	353
mock	7 dpi	2	host	+	22	915328
mock	7 dpi	2	host	−	22	465674
mock	7 dpi	2	unmapped	.	22	129937
mock	7 dpi	2	virus	+	24	128
mock	7 dpi	2	virus	−	24	114
mock	7 dpi	2	host	+	24	5213716
mock	7 dpi	2	host	−	24	3075212
mock	7 dpi	2	unmapped	.	24	669773
mock	7 dpi	2	virus	+	all	1117
mock	7 dpi	2	virus	−	all	1094
mock	7 dpi	2	host	+	all	10359426
mock	7 dpi	2	host	−	all	5788137
mock	7 dpi	2	unmapped	.	all	1951177
mock	7 dpi	3	virus	+	21	334

mock	7 dpi	3	virus	−	21	232
mock	7 dpi	3	host	+	21	1200057
mock	7 dpi	3	host	−	21	610788
mock	7 dpi	3	unmapped	.	21	179005
mock	7 dpi	3	virus	+	22	169
mock	7 dpi	3	virus	−	22	204
mock	7 dpi	3	host	+	22	1346617
mock	7 dpi	3	host	−	22	691810
mock	7 dpi	3	unmapped	.	22	182305
mock	7 dpi	3	virus	+	24	29
mock	7 dpi	3	virus	−	24	30
mock	7 dpi	3	host	+	24	6397060
mock	7 dpi	3	host	−	24	3728144
mock	7 dpi	3	unmapped	.	24	772605
mock	7 dpi	3	virus	+	all	697
mock	7 dpi	3	virus	−	all	627
mock	7 dpi	3	host	+	all	13367056
mock	7 dpi	3	host	−	all	7385924
mock	7 dpi	3	unmapped	.	all	2363181
mock	14 dpi	1	virus	+	21	789
mock	14 dpi	1	virus	−	21	693
mock	14 dpi	1	host	+	21	695413
mock	14 dpi	1	host	−	21	369866
mock	14 dpi	1	unmapped	.	21	81650
mock	14 dpi	1	virus	+	22	407
mock	14 dpi	1	virus	−	22	478
mock	14 dpi	1	host	+	22	805908
mock	14 dpi	1	host	−	22	415724
mock	14 dpi	1	unmapped	.	22	96738
mock	14 dpi	1	virus	+	24	93
mock	14 dpi	1	virus	−	24	72
mock	14 dpi	1	host	+	24	4321272
mock	14 dpi	1	host	−	24	2547719
mock	14 dpi	1	unmapped	.	24	511430
mock	14 dpi	1	virus	+	all	1735
mock	14 dpi	1	virus	−	all	1656
mock	14 dpi	1	host	+	all	8559199
mock	14 dpi	1	host	−	all	4817189
mock	14 dpi	1	unmapped	.	all	1534583
mock	14 dpi	2	virus	+	21	1563
mock	14 dpi	2	virus	−	21	1283
mock	14 dpi	2	host	+	21	635364
mock	14 dpi	2	host	−	21	333793
mock	14 dpi	2	unmapped	.	21	73459

mock	14 dpi	2	virus	+	22	784
mock	14 dpi	2	virus	−	22	874
mock	14 dpi	2	host	+	22	832277
mock	14 dpi	2	host	−	22	429652
mock	14 dpi	2	unmapped	.	22	97363
mock	14 dpi	2	virus	+	24	206
mock	14 dpi	2	virus	−	24	133
mock	14 dpi	2	host	+	24	4979894
mock	14 dpi	2	host	−	24	2993230
mock	14 dpi	2	unmapped	.	24	589592
mock	14 dpi	2	virus	+	all	3424
mock	14 dpi	2	virus	−	all	3078
mock	14 dpi	2	host	+	all	9175618
mock	14 dpi	2	host	−	all	5300838
mock	14 dpi	2	unmapped	.	all	1574112
mock	14 dpi	3	virus	+	21	1465
mock	14 dpi	3	virus	−	21	1220
mock	14 dpi	3	host	+	21	888192
mock	14 dpi	3	host	−	21	471621
mock	14 dpi	3	unmapped	.	21	106845
mock	14 dpi	3	virus	+	22	788
mock	14 dpi	3	virus	−	22	848
mock	14 dpi	3	host	+	22	1093864
mock	14 dpi	3	host	−	22	562598
mock	14 dpi	3	unmapped	.	22	129804
mock	14 dpi	3	virus	+	24	161
mock	14 dpi	3	virus	−	24	138
mock	14 dpi	3	host	+	24	6419086
mock	14 dpi	3	host	−	24	3845886
mock	14 dpi	3	unmapped	.	24	764725
mock	14 dpi	3	virus	+	all	3185
mock	14 dpi	3	virus	−	all	2893
mock	14 dpi	3	host	+	all	11938439
mock	14 dpi	3	host	−	all	6865971
mock	14 dpi	3	unmapped	.	all	2077932
AV2	7 dpi	1	virus	+	21	81896
AV2	7 dpi	1	virus	−	21	69623
AV2	7 dpi	1	host	+	21	999049
AV2	7 dpi	1	host	−	21	546714
AV2	7 dpi	1	unmapped	.	21	163759
AV2	7 dpi	1	virus	+	22	45422
AV2	7 dpi	1	virus	−	22	45133
AV2	7 dpi	1	host	+	22	1029517
AV2	7 dpi	1	host	−	22	525103

AV2	7 dpi	1	unmapped	.	22	159358
AV2	7 dpi	1	virus	+	24	5391
AV2	7 dpi	1	virus	−	24	4531
AV2	7 dpi	1	host	+	24	5624994
AV2	7 dpi	1	host	−	24	3287128
AV2	7 dpi	1	unmapped	.	24	779313
AV2	7 dpi	1	virus	+	all	187386
AV2	7 dpi	1	virus	−	all	166376
AV2	7 dpi	1	host	+	all	11413369
AV2	7 dpi	1	host	−	all	6338447
AV2	7 dpi	1	unmapped	.	all	2198590
AV2	7 dpi	2	virus	+	21	43781
AV2	7 dpi	2	virus	−	21	34181
AV2	7 dpi	2	host	+	21	916479
AV2	7 dpi	2	host	−	21	457744
AV2	7 dpi	2	unmapped	.	21	172149
AV2	7 dpi	2	virus	+	22	28955
AV2	7 dpi	2	virus	−	22	25748
AV2	7 dpi	2	host	+	22	1012069
AV2	7 dpi	2	host	−	22	507742
AV2	7 dpi	2	unmapped	.	22	187860
AV2	7 dpi	2	virus	+	24	4930
AV2	7 dpi	2	virus	−	24	2982
AV2	7 dpi	2	host	+	24	5401836
AV2	7 dpi	2	host	−	24	3206735
AV2	7 dpi	2	unmapped	.	24	724886
AV2	7 dpi	2	virus	+	all	116297
AV2	7 dpi	2	virus	−	all	88050
AV2	7 dpi	2	host	+	all	10938522
AV2	7 dpi	2	host	−	all	6052006
AV2	7 dpi	2	unmapped	.	all	2432103
AV2	7 dpi	3	virus	+	21	62424
AV2	7 dpi	3	virus	−	21	54734
AV2	7 dpi	3	host	+	21	1266850
AV2	7 dpi	3	host	−	21	656481
AV2	7 dpi	3	unmapped	.	21	154681
AV2	7 dpi	3	virus	+	22	40174
AV2	7 dpi	3	virus	−	22	40071
AV2	7 dpi	3	host	+	22	1453283
AV2	7 dpi	3	host	−	22	754158
AV2	7 dpi	3	unmapped	.	22	178617
AV2	7 dpi	3	virus	+	24	5291
AV2	7 dpi	3	virus	−	24	4802
AV2	7 dpi	3	host	+	24	6973629

AV2	7 dpi	3	host	−	24	4134853
AV2	7 dpi	3	unmapped	.	24	874838
AV2	7 dpi	3	virus	+	all	154063
AV2	7 dpi	3	virus	−	all	136658
AV2	7 dpi	3	host	+	all	14275505
AV2	7 dpi	3	host	−	all	8009527
AV2	7 dpi	3	unmapped	.	all	2553106
AV2	14 dpi	1	virus	+	21	150890
AV2	14 dpi	1	virus	−	21	131937
AV2	14 dpi	1	host	+	21	772030
AV2	14 dpi	1	host	−	21	403352
AV2	14 dpi	1	unmapped	.	21	109222
AV2	14 dpi	1	virus	+	22	91813
AV2	14 dpi	1	virus	−	22	94685
AV2	14 dpi	1	host	+	22	856996
AV2	14 dpi	1	host	−	22	437223
AV2	14 dpi	1	unmapped	.	22	116884
AV2	14 dpi	1	virus	+	24	12121
AV2	14 dpi	1	virus	−	24	12334
AV2	14 dpi	1	host	+	24	5139755
AV2	14 dpi	1	host	−	24	3032017
AV2	14 dpi	1	unmapped	.	24	590159
AV2	14 dpi	1	virus	+	all	333981
AV2	14 dpi	1	virus	−	all	310585
AV2	14 dpi	1	host	+	all	9581894
AV2	14 dpi	1	host	−	all	5406854
AV2	14 dpi	1	unmapped	.	all	1698819
AV2	14 dpi	2	virus	+	21	131349
AV2	14 dpi	2	virus	−	21	112081
AV2	14 dpi	2	host	+	21	673390
AV2	14 dpi	2	host	−	21	358323
AV2	14 dpi	2	unmapped	.	21	94780
AV2	14 dpi	2	virus	+	22	79447
AV2	14 dpi	2	virus	−	22	80229
AV2	14 dpi	2	host	+	22	814725
AV2	14 dpi	2	host	−	22	419359
AV2	14 dpi	2	unmapped	.	22	106645
AV2	14 dpi	2	virus	+	24	10259
AV2	14 dpi	2	virus	−	24	10080
AV2	14 dpi	2	host	+	24	5130658
AV2	14 dpi	2	host	−	24	3048568
AV2	14 dpi	2	unmapped	.	24	601212
AV2	14 dpi	2	virus	+	all	287925
AV2	14 dpi	2	virus	−	all	259739

AV2	14 dpi	2	host	+	all	9311113
AV2	14 dpi	2	host	−	all	5316208
AV2	14 dpi	2	unmapped	.	all	1622039
AV2	14 dpi	3	virus	+	21	116259
AV2	14 dpi	3	virus	−	21	101956
AV2	14 dpi	3	host	+	21	756952
AV2	14 dpi	3	host	−	21	405676
AV2	14 dpi	3	unmapped	.	21	94740
AV2	14 dpi	3	virus	+	22	77014
AV2	14 dpi	3	virus	−	22	80673
AV2	14 dpi	3	host	+	22	965815
AV2	14 dpi	3	host	−	22	498493
AV2	14 dpi	3	unmapped	.	22	113323
AV2	14 dpi	3	virus	+	24	10574
AV2	14 dpi	3	virus	−	24	10150
AV2	14 dpi	3	host	+	24	5724424
AV2	14 dpi	3	host	−	24	3397048
AV2	14 dpi	3	unmapped	.	24	649078
AV2	14 dpi	3	virus	+	all	265636
AV2	14 dpi	3	virus	−	all	244574
AV2	14 dpi	3	host	+	all	10447761
AV2	14 dpi	3	host	−	all	5976742
AV2	14 dpi	3	unmapped	.	all	1800072
AV2-2st	7 dpi	1	virus	+	21	786803
AV2-2st	7 dpi	1	virus	−	21	630903
AV2-2st	7 dpi	1	host	+	21	862000
AV2-2st	7 dpi	1	host	−	21	440807
AV2-2st	7 dpi	1	unmapped	.	21	226766
AV2-2st	7 dpi	1	virus	+	22	401503
AV2-2st	7 dpi	1	virus	−	22	427125
AV2-2st	7 dpi	1	host	+	22	818031
AV2-2st	7 dpi	1	host	−	22	410786
AV2-2st	7 dpi	1	unmapped	.	22	191654
AV2-2st	7 dpi	1	virus	+	24	88321
AV2-2st	7 dpi	1	virus	−	24	63639
AV2-2st	7 dpi	1	host	+	24	4216389
AV2-2st	7 dpi	1	host	−	24	2533803
AV2-2st	7 dpi	1	unmapped	.	24	567599
AV2-2st	7 dpi	1	virus	+	all	1612218
AV2-2st	7 dpi	1	virus	−	all	1434578
AV2-2st	7 dpi	1	host	+	all	8679920
AV2-2st	7 dpi	1	host	−	all	4857658
AV2-2st	7 dpi	1	unmapped	.	all	2009068
AV2-2st	7 dpi	2	virus	+	21	586591

AV2-2st	7 dpi	2	virus	−	21	508997
AV2-2st	7 dpi	2	host	+	21	857503
AV2-2st	7 dpi	2	host	−	21	443205
AV2-2st	7 dpi	2	unmapped	.	21	167184
AV2-2st	7 dpi	2	virus	+	22	345691
AV2-2st	7 dpi	2	virus	−	22	384369
AV2-2st	7 dpi	2	host	+	22	1012463
AV2-2st	7 dpi	2	host	−	22	517723
AV2-2st	7 dpi	2	unmapped	.	22	165559
AV2-2st	7 dpi	2	virus	+	24	73430
AV2-2st	7 dpi	2	virus	−	24	54284
AV2-2st	7 dpi	2	host	+	24	5230251
AV2-2st	7 dpi	2	host	−	24	3084689
AV2-2st	7 dpi	2	unmapped	.	24	649565
AV2-2st	7 dpi	2	virus	+	all	1230117
AV2-2st	7 dpi	2	virus	−	all	1167815
AV2-2st	7 dpi	2	host	+	all	9964675
AV2-2st	7 dpi	2	host	−	all	5613881
AV2-2st	7 dpi	2	unmapped	.	all	1942258
AV2-2st	7 dpi	3	virus	+	21	552816
AV2-2st	7 dpi	3	virus	−	21	439666
AV2-2st	7 dpi	3	host	+	21	785651
AV2-2st	7 dpi	3	host	−	21	404422
AV2-2st	7 dpi	3	unmapped	.	21	155251
AV2-2st	7 dpi	3	virus	+	22	301820
AV2-2st	7 dpi	3	virus	−	22	320952
AV2-2st	7 dpi	3	host	+	22	898216
AV2-2st	7 dpi	3	host	−	22	459754
AV2-2st	7 dpi	3	unmapped	.	22	151440
AV2-2st	7 dpi	3	virus	+	24	71443
AV2-2st	7 dpi	3	virus	−	24	51663
AV2-2st	7 dpi	3	host	+	24	4747985
AV2-2st	7 dpi	3	host	−	24	2839289
AV2-2st	7 dpi	3	unmapped	.	24	611546
AV2-2st	7 dpi	3	virus	+	all	1140637
AV2-2st	7 dpi	3	virus	−	all	1010579
AV2-2st	7 dpi	3	host	+	all	8882301
AV2-2st	7 dpi	3	host	−	all	5037836
AV2-2st	7 dpi	3	unmapped	.	all	1773338
AV2-2st	14 dpi	1	virus	+	21	500457
AV2-2st	14 dpi	1	virus	−	21	434532
AV2-2st	14 dpi	1	host	+	21	737781
AV2-2st	14 dpi	1	host	−	21	379320
AV2-2st	14 dpi	1	unmapped	.	21	158246

AV2-2st	14 dpi	1	virus	+	22	226080
AV2-2st	14 dpi	1	virus	−	22	262190
AV2-2st	14 dpi	1	host	+	22	855869
AV2-2st	14 dpi	1	host	−	22	438130
AV2-2st	14 dpi	1	unmapped	.	22	142326
AV2-2st	14 dpi	1	virus	+	24	63261
AV2-2st	14 dpi	1	virus	−	24	52838
AV2-2st	14 dpi	1	host	+	24	4399578
AV2-2st	14 dpi	1	host	−	24	2578037
AV2-2st	14 dpi	1	unmapped	.	24	583481
AV2-2st	14 dpi	1	virus	+	all	1091744
AV2-2st	14 dpi	1	virus	−	all	1077262
AV2-2st	14 dpi	1	host	+	all	8877328
AV2-2st	14 dpi	1	host	−	all	4940102
AV2-2st	14 dpi	1	unmapped	.	all	1777157
AV2-2st	14 dpi	2	virus	+	21	611986
AV2-2st	14 dpi	2	virus	−	21	510763
AV2-2st	14 dpi	2	host	+	21	862146
AV2-2st	14 dpi	2	host	−	21	446526
AV2-2st	14 dpi	2	unmapped	.	21	170055
AV2-2st	14 dpi	2	virus	+	22	272373
AV2-2st	14 dpi	2	virus	−	22	305109
AV2-2st	14 dpi	2	host	+	22	983602
AV2-2st	14 dpi	2	host	−	22	504189
AV2-2st	14 dpi	2	unmapped	.	22	148657
AV2-2st	14 dpi	2	virus	+	24	71808
AV2-2st	14 dpi	2	virus	−	24	60454
AV2-2st	14 dpi	2	host	+	24	5204807
AV2-2st	14 dpi	2	host	−	24	3094605
AV2-2st	14 dpi	2	unmapped	.	24	649306
AV2-2st	14 dpi	2	virus	+	all	1318672
AV2-2st	14 dpi	2	virus	−	all	1249833
AV2-2st	14 dpi	2	host	+	all	10312606
AV2-2st	14 dpi	2	host	−	all	5810738
AV2-2st	14 dpi	2	unmapped	.	all	1947214
AV2-2st	14 dpi	3	virus	+	21	753070
AV2-2st	14 dpi	3	virus	−	21	640413
AV2-2st	14 dpi	3	host	+	21	956017
AV2-2st	14 dpi	3	host	−	21	491360
AV2-2st	14 dpi	3	unmapped	.	21	215224
AV2-2st	14 dpi	3	virus	+	22	340707
AV2-2st	14 dpi	3	virus	−	22	388792
AV2-2st	14 dpi	3	host	+	22	1086332
AV2-2st	14 dpi	3	host	−	22	556084

AV2-2st	14 dpi	3	unmapped	.	22	181428
AV2-2st	14 dpi	3	virus	+	24	87592
AV2-2st	14 dpi	3	virus	−	24	69277
AV2-2st	14 dpi	3	host	+	24	5542900
AV2-2st	14 dpi	3	host	−	24	3276754
AV2-2st	14 dpi	3	unmapped	.	24	766900
AV2-2st	14 dpi	3	virus	+	all	1625210
AV2-2st	14 dpi	3	virus	−	all	1569510
AV2-2st	14 dpi	3	host	+	all	11200593
AV2-2st	14 dpi	3	host	−	all	6274925
AV2-2st	14 dpi	3	unmapped	.	all	2240760

Supplementary Table E. Comparisons of vsiRNA read counts across different samples and timepoints. Each count (columns 4 and 5) is based on the average of three replicates where, for each replicate, the sum of positive-sense and negative-sense virus-mapping read counts from Supplementary Table D is multiplied by the corresponding normalisation factor from Supplementary Table F.

Comparison (group1 − group 2)	Read length (nt)	Time point	Mean read count – group 1 (normalised)	Mean read count – group 2 (normalised)	Mean difference	*t*	df	*p*-value	Fold change
AV2 − AV2-2st	21	7 dpi	28464	301891	273427	−18.4	2	0.0000	10.6
AV2 − AV2-2st	21	14 dpi	104040	420645	316605	−18.7	2	0.0000	4.0
AV2 − AV2-2st	22	7 dpi	18380	188607	170226	−23.7	2	0.0000	10.3
AV2 − AV2-2st	22	14 dpi	70457	218828	148370	−15.3	2	0.0001	3.1
AV2 − AV2-2st	24	7 dpi	2260	34871	32610	−121.3	2	0.0000	15.4
AV2 − AV2-2st	24	14 dpi	9150	49663	40512	−23.5	2	0.0000	5.4
AV2 − AV2-2st	all	7 dpi	69471	654452	584981	−19.4	2	0.0000	9.4
AV2 − AV2-2st	all	14 dpi	237837	967540	729702	−18.9	2	0.0000	4.1
7 dpi − 14 dpi AV2	21	–	28464	104040	75576	−8.0	2	0.0006	3.7
7 dpi − 14 dpi AV2	22	–	18380	70457	52077	−11.7	2	0.0002	3.8
7 dpi − 14 dpi AV2	24	–	2260	9150	6889	−18.8	2	0.0000	4.0
7 dpi − 14 dpi AV2	all	–	69471	237837	168366	−8.9	2	0.0004	3.4
7 dpi − 14 dpi AV2-2st	21	–	301891	420645	118754	−5.8	2	0.0022	1.4
7 dpi − 14 dpi AV2-2st	22	–	188607	218828	30221	−2.7	2	0.0271	1.2
7 dpi − 14 dpi AV2-2st	24	–	34871	49663	14791	−8.7	2	0.0005	1.4
7 dpi − 14 dpi AV2-2st	all	–	654452	967540	313087	−6.9	2	0.0011	1.5

Supplementary Table F. Normalisation factors for each library. These were calculated based on the total number of 21 nt reads mapped in positive sense to the curated set of 128 miRNAs (see main text). (Note, the products of the values in columns 4 and 5 equates to 34477, the size of the smallest library i.e. mock 14 dpi replicate 2.)

Virus	Timepoint	Replicate	Number of reads	Normalisation factor
AV2-2st	14 dpi	1	76778	0.4490
AV2-2st	7 dpi	1	149678	0.2303
AV2-2st	14 dpi	2	98284	0.3508
AV2-2st	7 dpi	2	126994	0.2715
AV2-2st	14 dpi	3	107183	0.3217
AV2-2st	7 dpi	3	121476	0.2838
mock	14 dpi	1	75187	0.4586
mock	7 dpi	1	144431	0.2387
mock	14 dpi	2	34477	1.0000
mock	7 dpi	2	95164	0.3623
mock	14 dpi	3	62630	0.5505
mock	7 dpi	3	109721	0.3142
AV2	14 dpi	1	95636	0.3605
AV2	7 dpi	1	124258	0.2775
AV2	14 dpi	2	72417	0.4761
AV2	7 dpi	2	140068	0.2461
AV2	14 dpi	3	79809	0.4320
AV2	7 dpi	3	167169	0.2062

Supplementary Figure A. Histograms of median length across a cluster of the ORF in-frame with and immediately 3′-adjacent to the CP stop codon, for sequences with (pink) and sequences without (black) a putative RT domain. Data from columns 3 and 4 of Supplementary Table A.

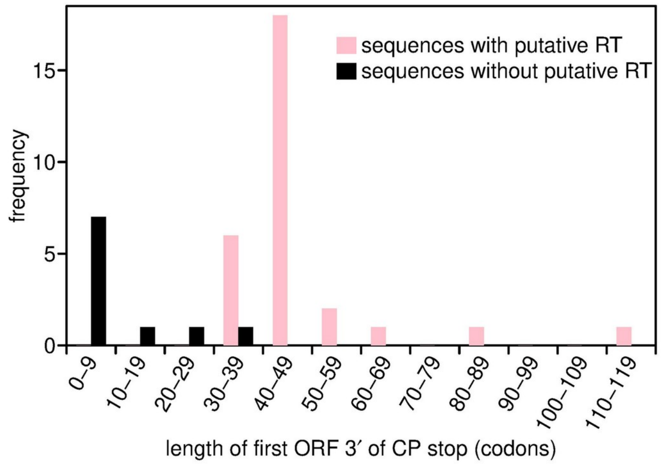

Supplementary Figure B. Phylogenetic tree of the *Ilarvirus* CP protein. CP amino acid sequences translated from reference sequences (one for each cluster) were aligned with MUSCLE [7] and a phylogenetic tree was estimated using the Bayesian Markov chain Monte Carlo method implemented in MrBayes version 3.2.3 [4], sampling across the default set of fixed amino acid rate matrices, with one million generations, discarding the first 25% as burn-in. The tree was visualized with FigTree (http://tree.bio.ed.ac.uk/software/figtree/). Reference sequences that have a putative readthrough domain (as defined by the apparent presence of a lengthy insert in RNA3 when the 3′-of-CP region was compared with the 3′UTRs of RNAs 2 and 3) are in red. Sequences for which this is unknown due to the absence of appropriate RNA1/RNA2 sequence data are in purple. Sequences that are proposed not to have a readthrough domain are in black. The tree is midpoint rooted and nodes are labelled with posterior probability values. Note that apple mosaic virus and lilac leaf chlorosis virus each occur twice on the tree since each species has representatives in >1 clusters (indicated by the cluster suffixes 6a/6b and 28/31, respectively) – see Supplementary Data C for details.

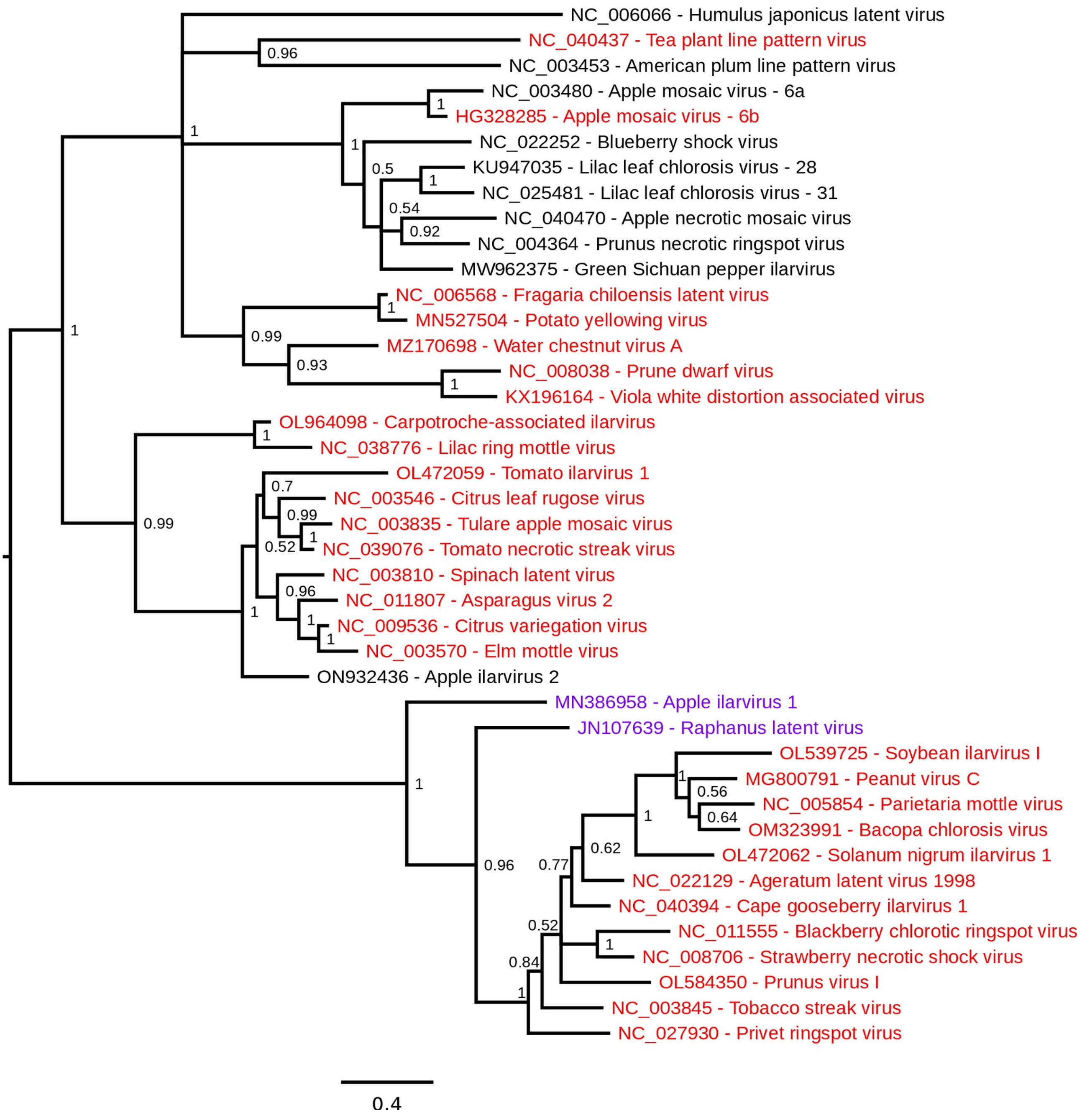

Supplementary Figure C. Phylogenetic tree of the *Ilarvirus* MP protein. MP amino acid sequences were translated from the same set of reference sequences used in Supplementary Figure B. Other details are as described in the Supplementary Figure B caption.

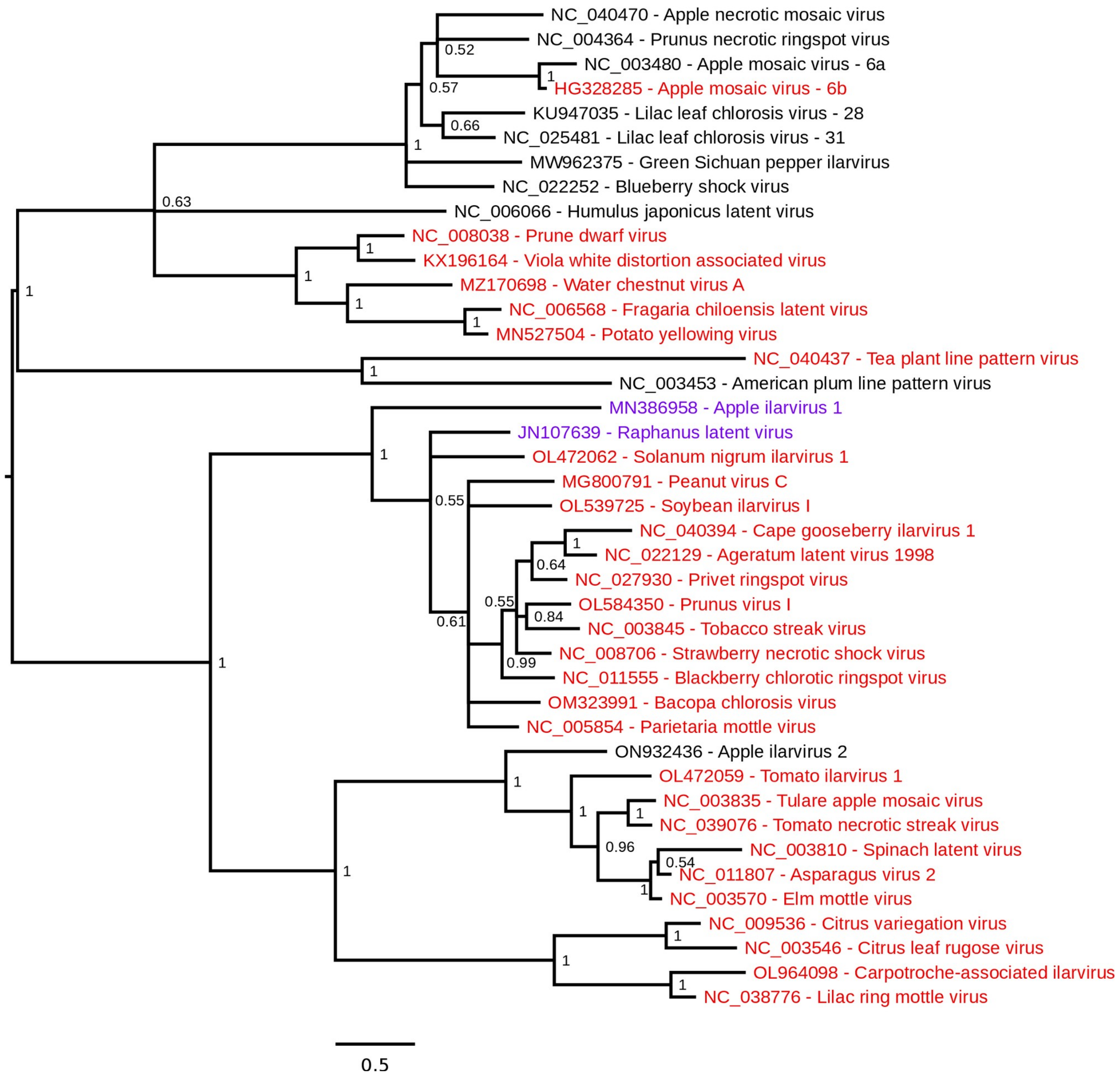

Supplementary Figure D. Phylogenetic tree of the *Ilarvirus* ORF1 protein. ORF1 amino acid sequences were translated from a set of RNA1 reference sequences matched to the RNA3 refererence sequences used in Supplementary Figure B and elsewhere in this study. Other details are as described in the Supplementary Figure B caption. Note that, due to the absence of matched RNA1 sequences for the RNA3 sequences HG328285, MN386958 and JN107639, these species are excluded from the ORF1 tree.

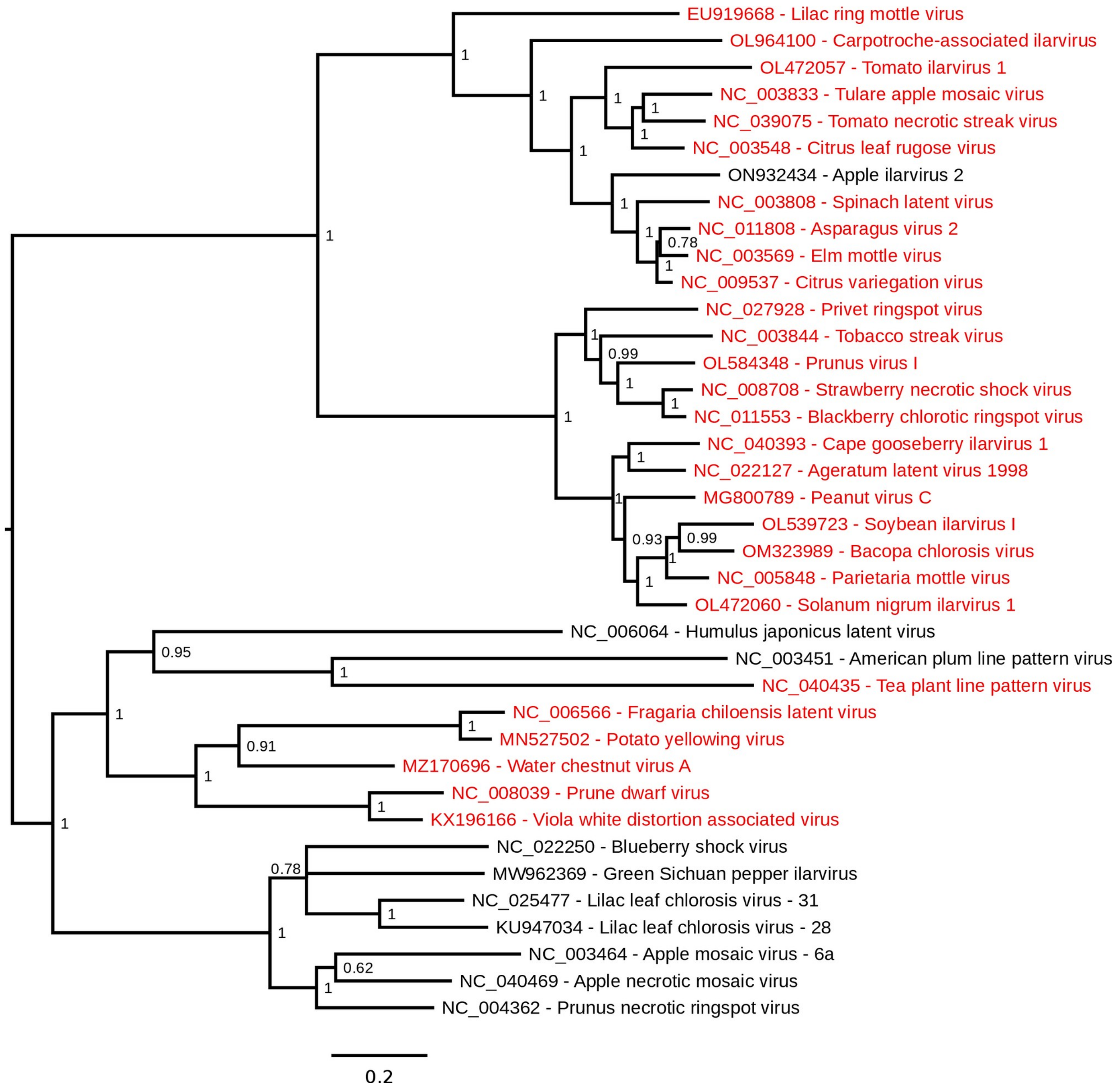

Supplementary Figure E. Phylogenetic tree of the *Ilarvirus* ORF2a protein. ORF2a amino acid sequences were translated from a set of RNA2 reference sequences matched to the RNA3 refererence sequences used in Supplementary Figure B and elsewhere in this study. Other details are as described in the Supplementary Figure B caption. Note that, due to the absence of matched RNA2 sequences for the RNA3 sequences HG328285, MN386958 and JN107639, these species are excluded from the ORF2a tree.

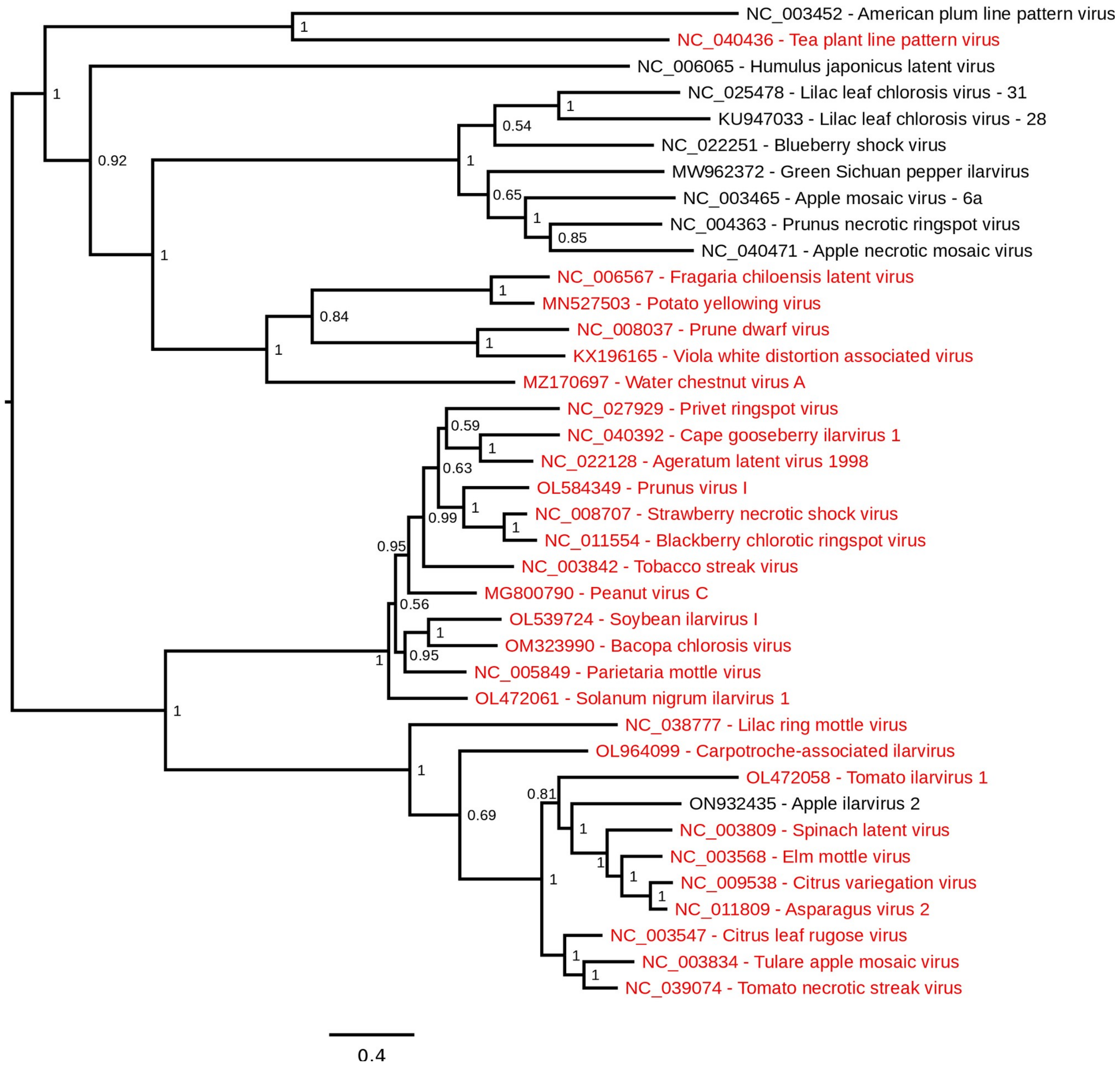

Supplementary Figure F. Amino acid sequences of the putative readthrough domains in the cluster reference sequences. Columns are: cluster; reference sequence accession number; readthrough peptide sequence. Sequences are ordered according to the CP phylogenetic tree (Supplementary Figure B), and clades based on the tree are separated by blank lines. In the case of NC_008706, the peptide is shown after insertion of the frame-correcting "A" nucleotide (see Supplementary Dataset C for details). All cysteine and histidine residues are highlighted.

```
37   NC_040437   LHSGDYLSELEYDPMSGWFLNRGVRWYYCPIPACVFTNGYRLGEDH*

6b   HG328285    QSGSQISNIVRIFDNLDDLMERFAPYAEGA*

5a   NC_006568   QLGRDISRLRDVSRRAKVPNDFIVGCEPAYDIDMCEQAHDGYPEVNPAVLRPRFFSVPRT
                 VRRSRRLNPRLVDEANLPYFTGECVRCGYTPKSLIDQREWECSSCYMLYAA*
5b   MN527504    QLGRVNTQFRVLARRVEIPGIDPRCEFDAQGSLTFRSRSVPRVFNRPRRLNPRLSKEEEE
                 ALDHFDQVDNSDGADYLDDWRSYVD*

27   MZ170698    LHSGGVNFDTWNGICSRCGFKPKRLVDQYTYTCRECGKIYFA*
3    NC_008038   LYDPSFDCASTMSIPRNIRSWKCCFCNRIHHSEFVTEC*
23   KX196164    LYDPFFDCSDSANFSFLPPEEMEFKAWRCCYCNEIHYV*

34   OL964098    PHSGGRCLVCEVSWGLQTYTRIDDFSLRDECLYCGADAEIGLLCPSFKCPQGCTQCL*
35   NC_038776   PHSGSRCLVCGTSWSLQTYTYLEDFSLRDECLLCGADAEIGLICPSFKCPLGCTQYPCFG*

33   OL472059    PRSGCESCGYVPYCHCKCYNDACSVVGCEQNIWFIPDEVFP*
18   NC_003546   PQDCESCGYIPYCHCECWPYRCYTIGCNANDLWFIDS*
17a  NC_003835   LHSGFCPVCGYVPLCHCMCYNYSCDVIGCDANDTFCIEDDQV*
17b  NC_039076   PRSGLCQSCGFIPYCHCYCCDDVCDVIGCLANDTVYDEV*
13   NC_003810   LHSDDCSSRNVIPFCHCSCCPFHCDVIGCVCNDAFFYED*
14   NC_011807   PRSGECVYLHSNECEKKNVIPFCHCECYDNACNTLWCPANVFCFHEP*
8a   NC_009536   LHSDDCPSQSVIPFCHCECYDNACGTPWCPANANCFHEFHENY*
8b   NC_003570   LHSGDYLHSVECERKCVIPFCHCVCYDDACDVLWCSPNDLCYHESNDW*

25   OL539725    RSTSSGSELPCIMNNSCARCGFRPPYVIDQYIWSCALCHYKHYA*
36   MG800791    KVAASGVVPEMPHVDGNSCVRCGYTPKYLIDSYIWTCFMCSYEQYA*
12   NC_005854   MVVTDVSGVDANVKSDDSYQDGDYYYDTDMFQYDCTDDDDGTAYMQDFFSCVAAMNF*
20   OM323991    RSQSSHDEMPHVKNNSCVRCGFRPPQIIDQYVWRCRRCYLEHAA*
10   OL472062    RSQVPSPRLELPYFTEKCVRCGFAPKYTVDQYIWKCHVCSYEHYA*
30   NC_022129   LTADSSSTVVDELPHMDGNSCVRCGFTPKRIVDQYVWRCYLCDYPQMA*
15   NC_040394   LSDMPQVNELPYITNCCVRCGFKPRRVVDQLVWSCTFCLYKQRA*
4    NC_011555   LVNYLEENFEVPHILPTIVGSCVRCGFRPRRVPDARYLWCGMCKYLHYA*
11   NC_008706   LSPILEFDLELPHIKNNSCVRCGYHPMYVVDQHIWKCDVCHQRHYA*
16   OL584350    LLSDLDNVELPHMRSSCVRCGFCPKYAVDQLIWRCHLCGYPHSA*
1    NC_003845   LDGHLGETEMPHIDGNSCVRCGFTPRRIVDQYIWKCDICSYEQYA*
29   NC_027930   LDGLVTSGRVSDLPHIDGNSCVRCGYAPRYIVDQCYWECTLCSYEQRA*
```

Supplementary Figure G. Positions of start and stop codons in ilarvirus reference sequences. RNAs 1, 2 and 3 are shown at left, middle and right, respectively. For each RNA, the three tracks show the positions of stop codons (black) and AUG codons (green) in each of the three positive-sense reading frames. Note that EU919668, JN107639, KX196166 and KX196165 (at least) are not coding-complete and, in addition, various sequences are missing various amounts of the 5′ and/or 3′ UTR.

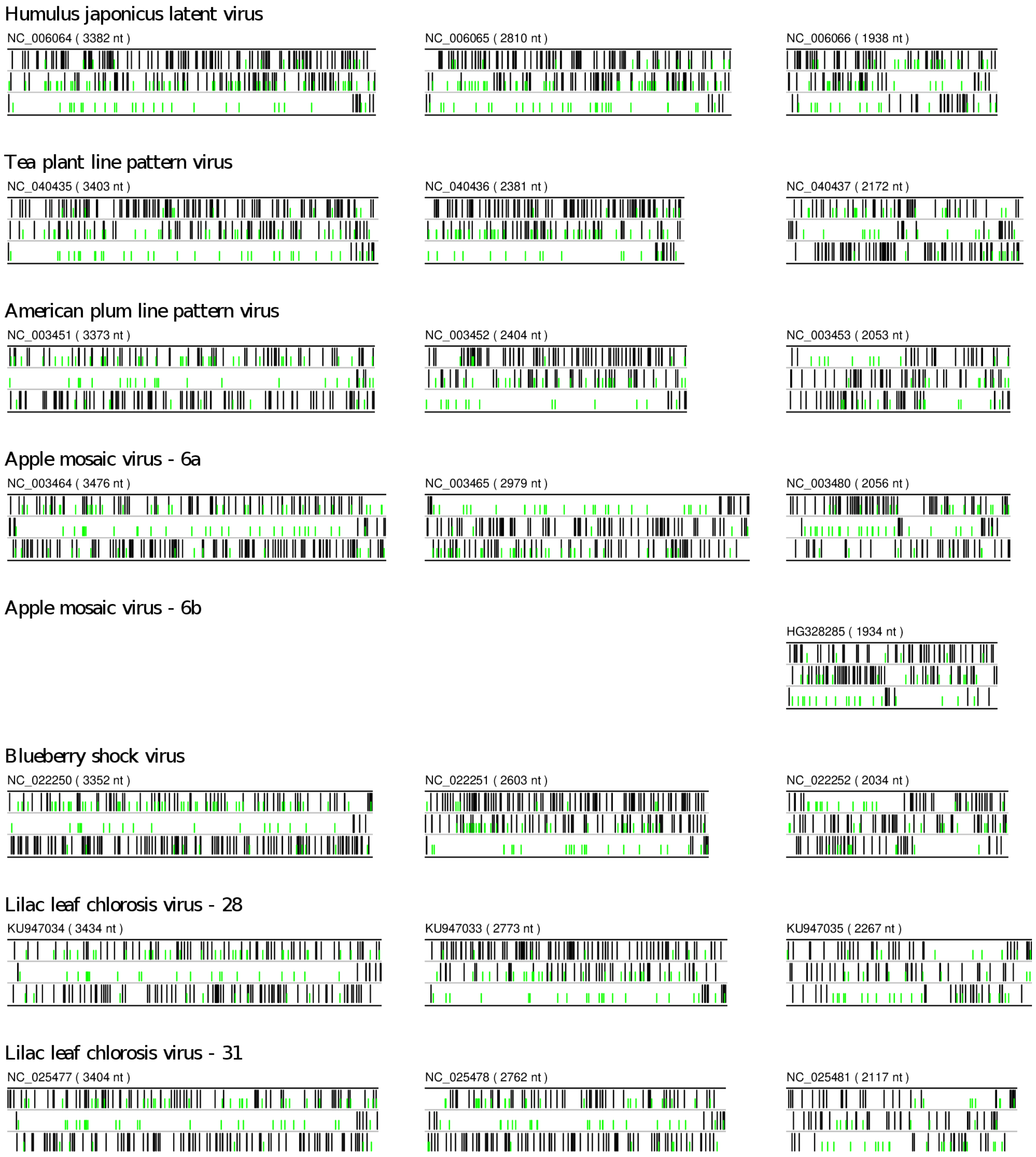

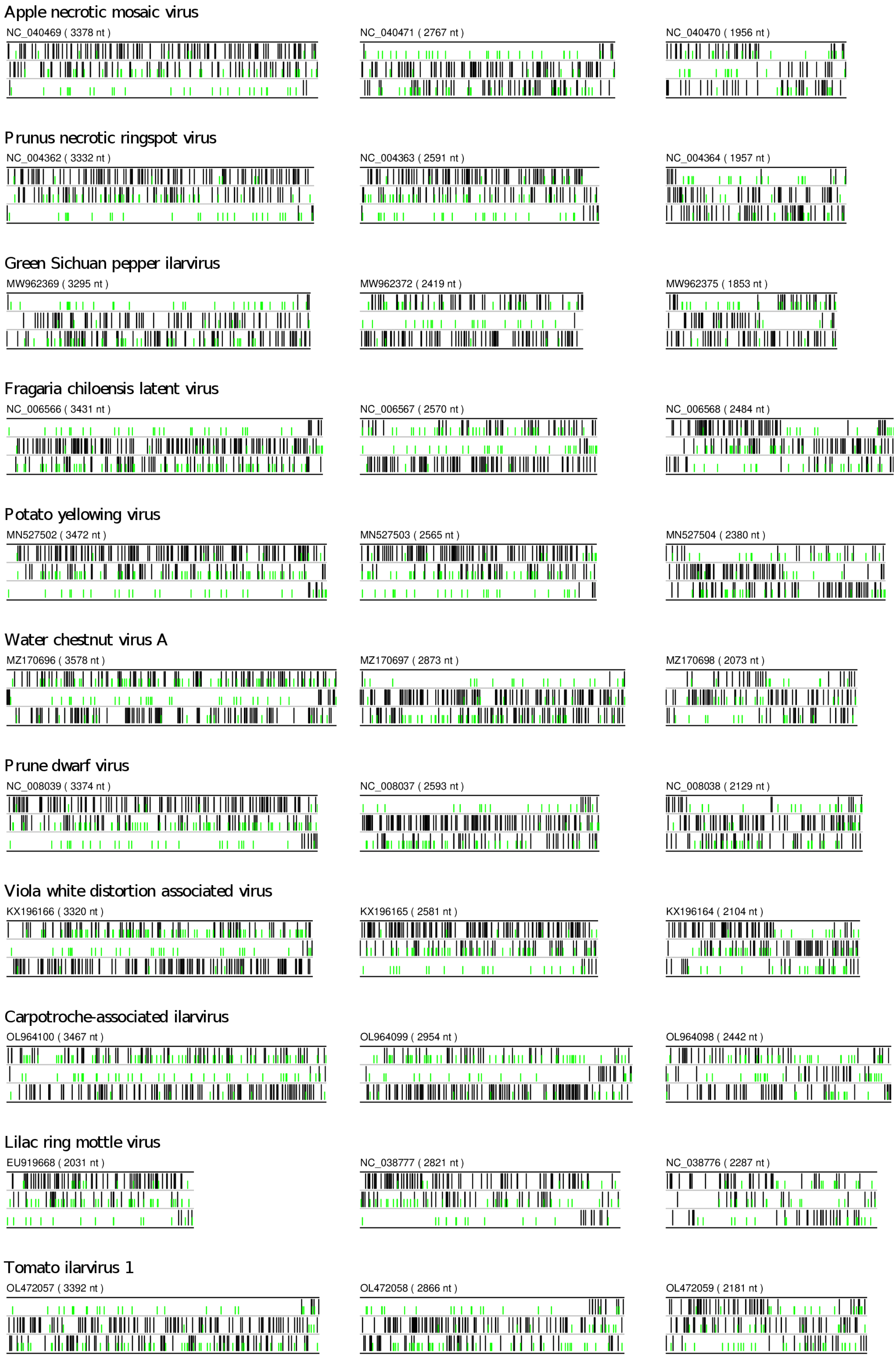
Apple necrotic mosaic virus
NC_040469 (3378 nt)
NC_040471 (2767 nt)
NC_040470 (1956 nt)
Prunus necrotic ringspot virus
NC_004362 (3332 nt)
NC_004363 (2591 nt)
NC_004364 (1957 nt)
Green Sichuan pepper ilarvirus
MW962369 (3295 nt)
MW962372 (2419 nt)
MW962375 (1853 nt)
Fragaria chiloensis latent virus
NC_006566 (3431 nt)
NC_006567 (2570 nt)
NC_006568 (2484 nt)
Potato yellowing virus
MN527502 (3472 nt)
MN527503 (2565 nt)
MN527504 (2380 nt)
Water chestnut virus A
MZ170696 (3578 nt)
MZ170697 (2873 nt)
MZ170698 (2073 nt)
Prune dwarf virus
NC_008039 (3374 nt)
NC_008037 (2593 nt)
NC_008038 (2129 nt)
Viola white distortion associated virus
KX196166 (3320 nt)
KX196165 (2581 nt)
KX196164 (2104 nt)
Carpotroche-associated ilarvirus
OL964100 (3467 nt)
OL964099 (2954 nt)
OL964098 (2442 nt)
Lilac ring mottle virus
EU919668 (2031 nt)
NC_038777 (2821 nt)
NC_038776 (2287 nt)
Tomato ilarvirus 1
OL472057 (3392 nt)
OL472058 (2866 nt)
OL472059 (2181 nt)

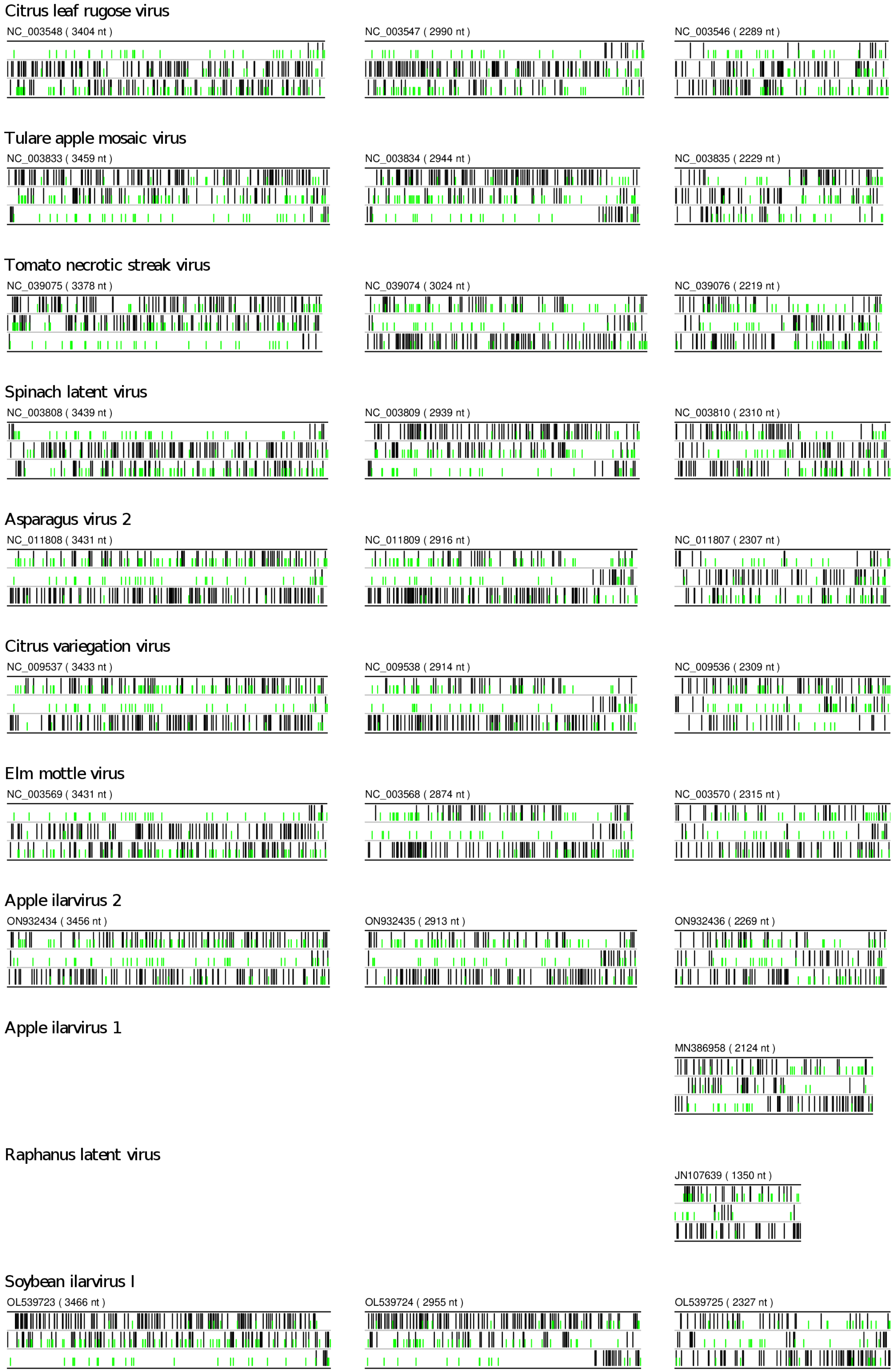

Citrus leaf rugose virus
NC_003548 (3404 nt)
NC_003547 (2990 nt)
NC_003546 (2289 nt)
Tulare apple mosaic virus
NC_003833 (3459 nt)
NC_003834 (2944 nt)
NC_003835 (2229 nt)
Tomato necrotic streak virus
NC_039075 (3378 nt)
NC_039074 (3024 nt)
NC_039076 (2219 nt)
Spinach latent virus
NC_003808 (3439 nt)
NC_003809 (2939 nt)
NC_003810 (2310 nt)
Asparagus virus 2
NC_011808 (3431 nt)
NC_011809 (2916 nt)
NC_011807 (2307 nt)
Citrus variegation virus
NC_009537 (3433 nt)
NC_009538 (2914 nt)
NC_009536 (2309 nt)
Elm mottle virus
NC_003569 (3431 nt)
NC_003568 (2874 nt)
NC_003570 (2315 nt)
Apple ilarvirus 2
ON932434 (3456 nt)
ON932435 (2913 nt)
ON932436 (2269 nt)
Apple ilarvirus 1
MN386958 (2124 nt)
Raphanus latent virus
JN107639 (1350 nt)
Soybean ilarvirus I
OL539723 (3466 nt)
OL539724 (2955 nt)
OL539725 (2327 nt)

Peanut virus C

MG800789 (3474 nt) MG800790 (2925 nt) MG800791 (2160 nt)

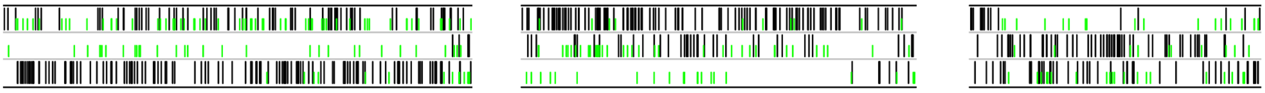

Parietaria mottle virus

NC_005848 (3518 nt) NC_005849 (2922 nt) NC_005854 (2268 nt)

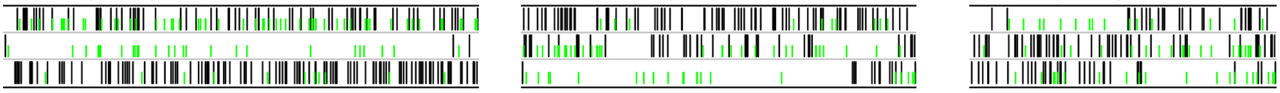

Bacopa chlorosis virus

OM323989 (3462 nt) OM323990 (2969 nt) OM323991 (2321 nt)

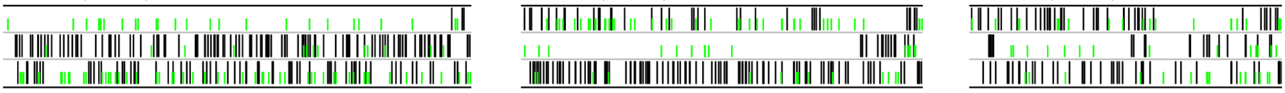

Solanum nigrum ilarvirus 1

OL472060 (3445 nt) OL472061 (2842 nt) OL472062 (2272 nt)

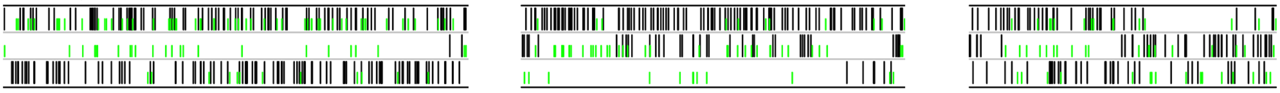

Ageratum latent virus 1998

NC_022127 (3481 nt) NC_022128 (2872 nt) NC_022129 (2224 nt)

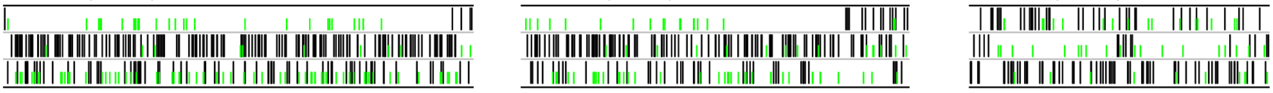

Cape gooseberry ilarvirus 1

NC_040393 (3493 nt) NC_040392 (2862 nt) NC_040394 (2234 nt)

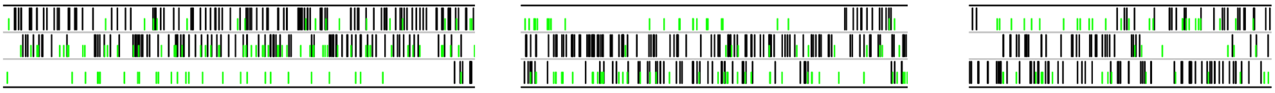

Blackberry chlorotic ringspot virus

NC_011553 (3478 nt) NC_011554 (2879 nt) NC_011555 (2290 nt)

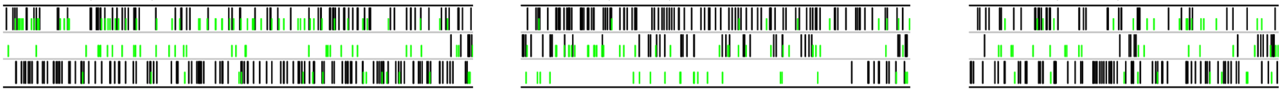

Strawberry necrotic shock virus

NC_008708 (3429 nt) NC_008707 (2876 nt) NC_008706 (2245 nt)

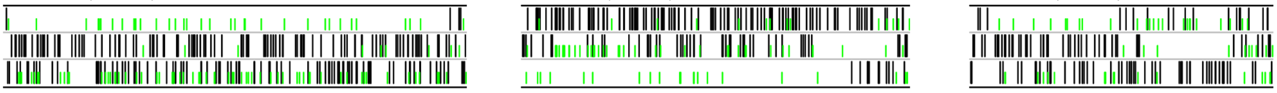

Prunus virus I

OL584348 (3478 nt) OL584349 (2910 nt) OL584350 (2225 nt)

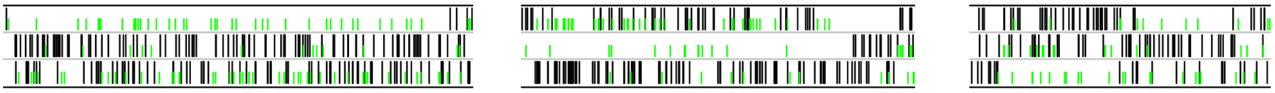

Tobacco streak virus

NC_003844 (3491 nt) NC_003842 (2926 nt) NC_003845 (2205 nt)

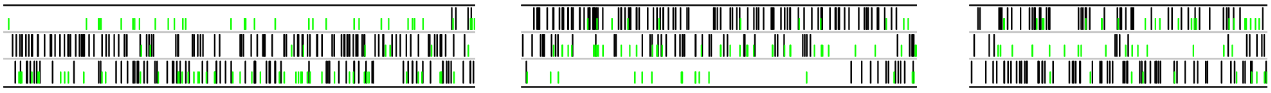

Privet ringspot virus

NC_027928 (3415 nt) NC_027929 (2863 nt) NC_027930 (2140 nt)

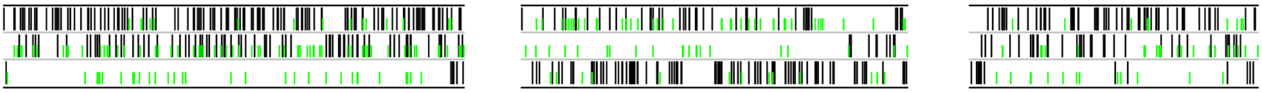

Supplementary Figure H. RNA-seq analysis of AV2 and AV2-2st viruses. *N. benthamiana* plants were agroinfected with AV2 or AV2-2st, and systemically infected leaves were harvested at 7 dpi for RNA-seq. Libraries were generated for three separate plants for each virus. Note these samples are not paired with the Ribo-seq samples. **(i)** RNA-seq density on the AV2 and AV2-2st genomes. Green – read coverage of vRNA(+); brown – read coverage of vRNA(−) (scaled 100×). **(ii)** Mean RNA-seq density for each segment relative to RNA 2.

(i)

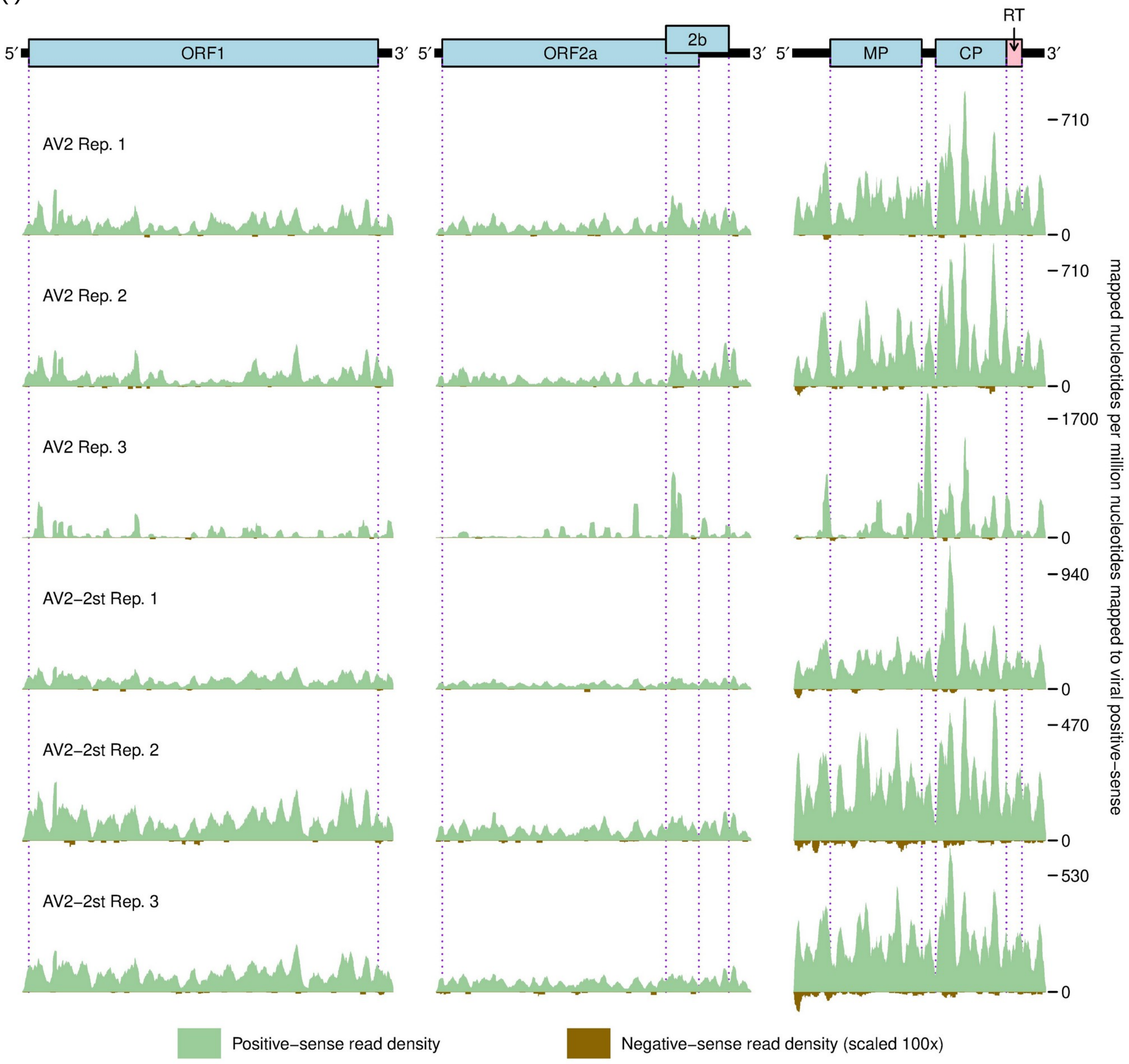

(ii)

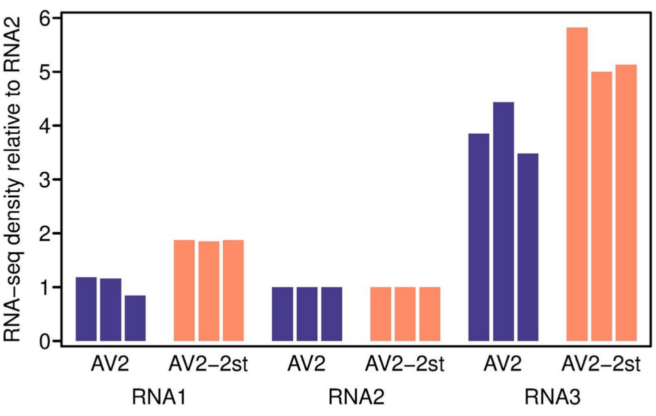

Supplementary Figure I. Detection of CP and CP-RT-HA by western blot. **(i)** Plants were infected with AV2 or AV2-HA, and samples were collected from the inoculated leaf and 2nd non-inoculated leaf at 4 dpi and 7 dpi, and from the 3rd non-inoculated leaf at 14 dpi. **(ii)** Plants were infected with AV2, AV2-HA or AV2-2st-HA, and samples were collected from the 2nd non-inoculated leaf at 4 dpi. Sizes of molecular weight markers are indicated on the right. Ponceau red staining (lower panels) of the large Rubisco subunit (RbcL) was used as a loading control.

(i)

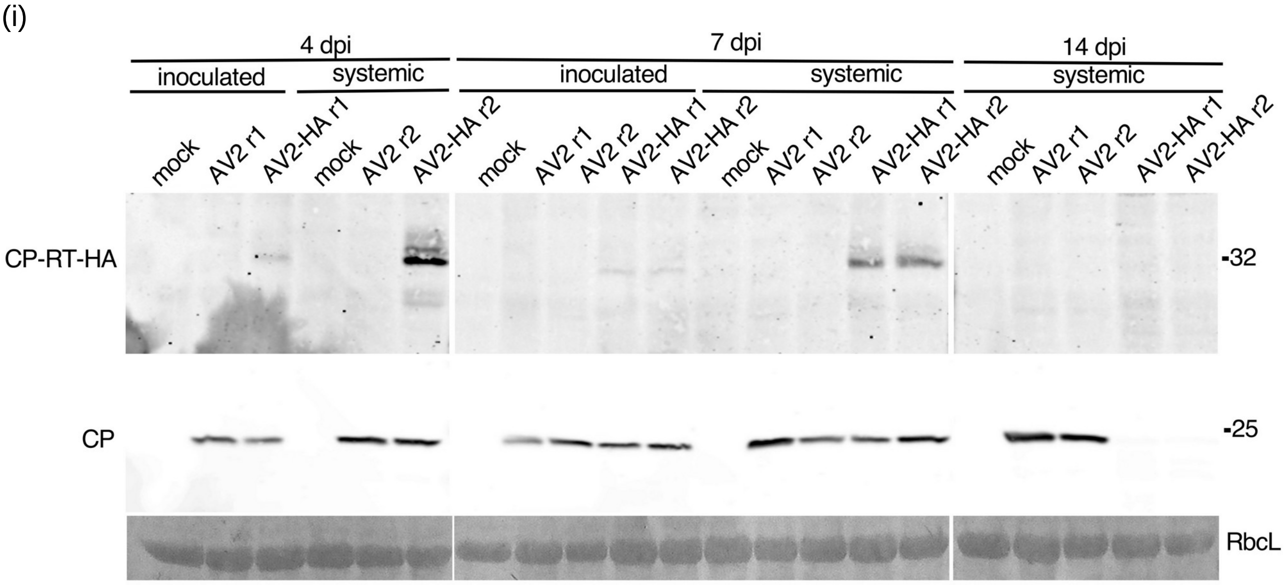

(ii)

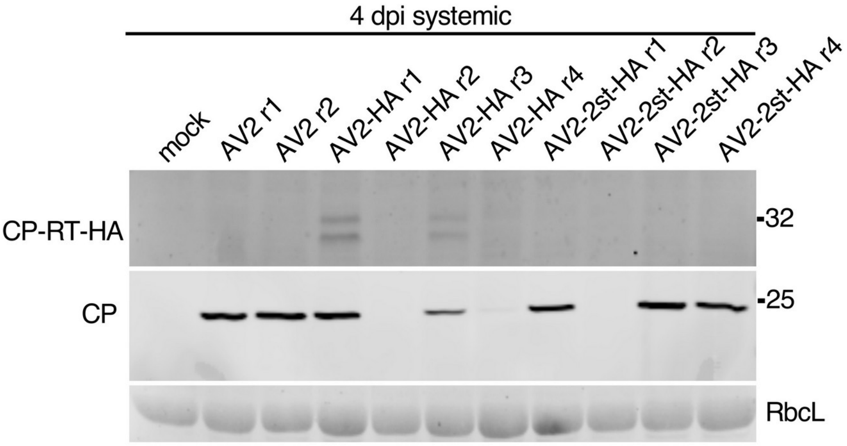

Supplementary Figure J. Quality assessment of ribosome profiling samples for *Nicotiana benthamiana* agroinfected with AV2 or AV2-2st. **(i)** Phasing of 5′ ends of 27-nt and 28-nt RPFs that map to the viral ORFs (excluding dual coding regions) or host mRNA coding regions. **(ii)** Histograms of approximate P-site positions of 27-nt and 28-nt RPFs relative to annotated initiation and termination sites summed over all host mRNAs.

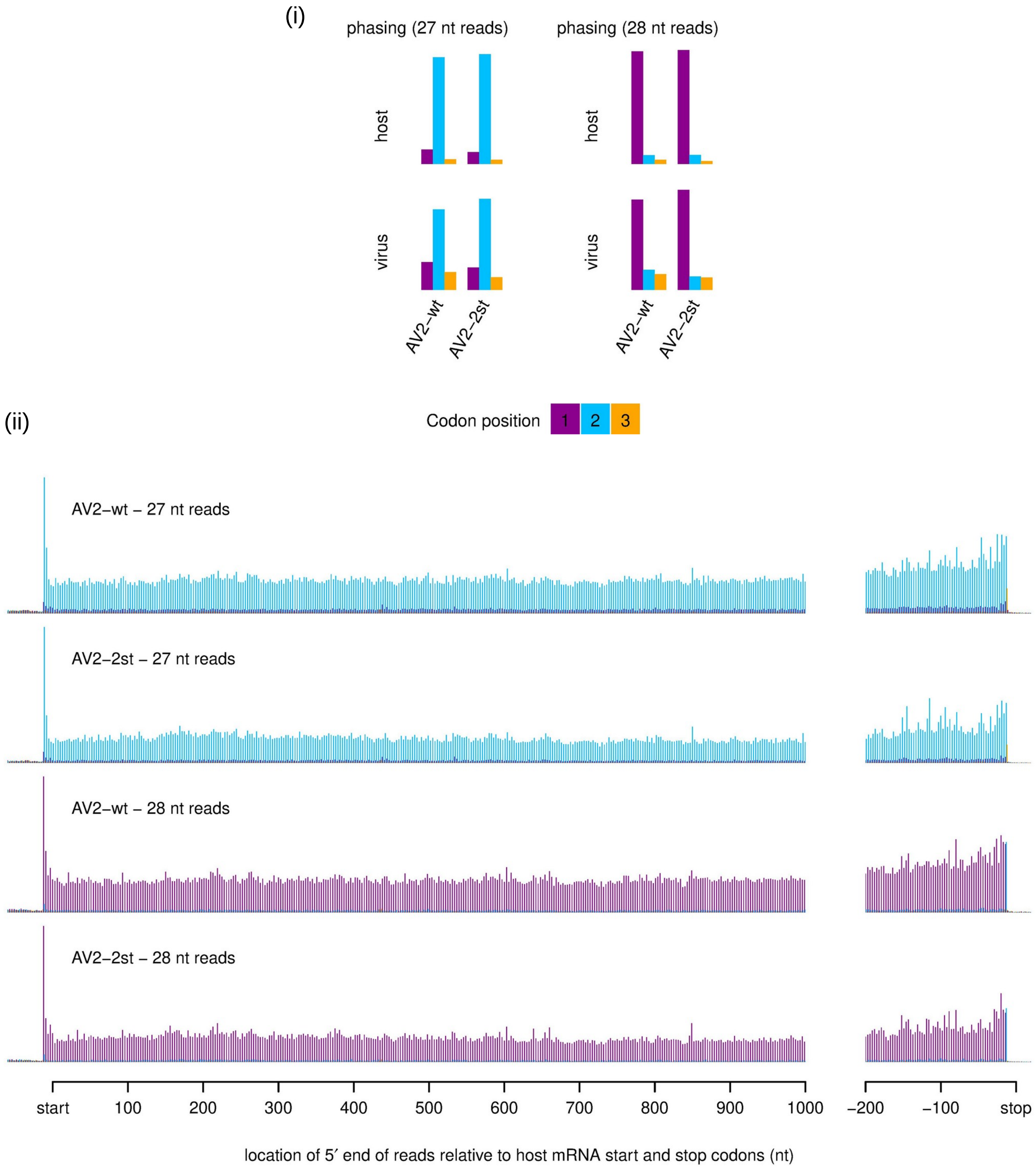

Supplementary Figure K. Distribution of 27 nt ribosome profiling reads on the AV2 and AV2-2st genomes*. N. benthamiana* plants were agroinfected with AV2 or AV2-2st, and systemically infected leaves were harvested at 12 dpi for Ribo-seq. Histograms show the positions of the 5′ ends of reads, with a +12 nt offset to map approximate P-site positions. Colours purple, blue and orange indicate the three different phases relative to the reading frame of ORFs 1, 2a and CP in RNAs 1, 2 and 3 respectively. Therefore, consistent with Supplementary Figure J(i), most 27 nt reads map to the blue phase in ORFs 1, 2a, and CP as well as MP; reads map predominantly to the yellow phase in ORF2b, and true RPF reads are expected to map to the blue phase in the RT ORF. Note that nucleotide-to-nucleotide variation in RPF counts may be influenced by technical biases besides ribosome codon dwell-times. Reads in the 3′UTR and a fraction of reads throughout the genome undoubtably derive from non-RPF contamination, and contamination is likely to be relatively more pronounced in the lowly expressed AV2-2st (cf. Supplementary Table C). Reads that map to the 3′UTR sequences that are shared between all three RNAs cannot be assigned to a specific RNA; the mapping algorithm, bowtie [5], assigns such multimapping reads at random; thus many reads that probably arise from the highly expressed RNA3 3′UTR would be mismapped to the lowly expressed RNA1 and RNA2 3′UTRs. Therefore, for each RNA, 3′UTR peaks have been truncated to a maximum of 1.25 times the peak value outside of the 3′UTR conserved-sequence region.

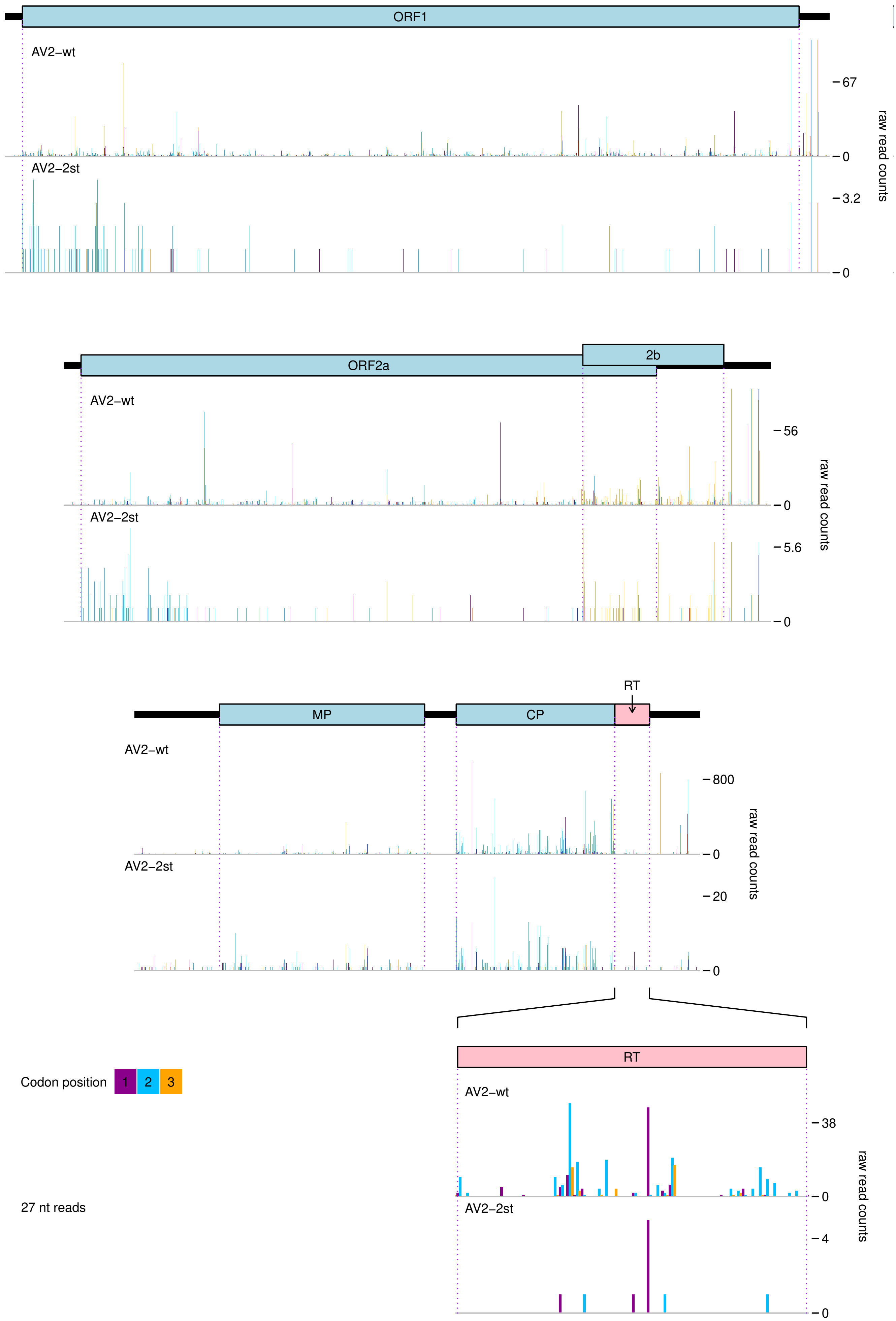
ORF1
AV2–wt
AV2–2st
67
0
3.2
0
raw read counts
ORF2a
2b
AV2–wt
AV2–2st
56
0
5.6
0
raw read counts
RT
MP
CP
AV2–wt
AV2–2st
800
0
20
0
raw read counts
RT
AV2–wt
AV2–2st
38
0
4
0
raw read counts
Codon position
1
2
3
27 nt reads

Supplementary Figure L. Distribution of 28 nt ribosome profiling reads on the AV2 and AV2-2st genomes. *N. benthamiana* plants were agroinfected with AV2 or AV2-2st, and systemically infected leaves were harvested at 12 dpi for Ribo-seq. Histograms show the positions of the 5′ ends of reads, with a +12 nt offset to map approximate P-site positions. Colours purple, blue and orange indicate the three different phases relative to the reading frame of ORFs 1, 2a and CP in RNAs 1, 2 and 3 respectively. Therefore, consistent with Supplementary Figure J(i), most 28 nt reads map to the purple phase in ORFs 1, 2a, and CP as well as MP; reads map predominantly to the blue phase in ORF2b, and true RPF reads are expected to map to the purple phase in the RT ORF. See the Supplementary Figure K caption for additional details.

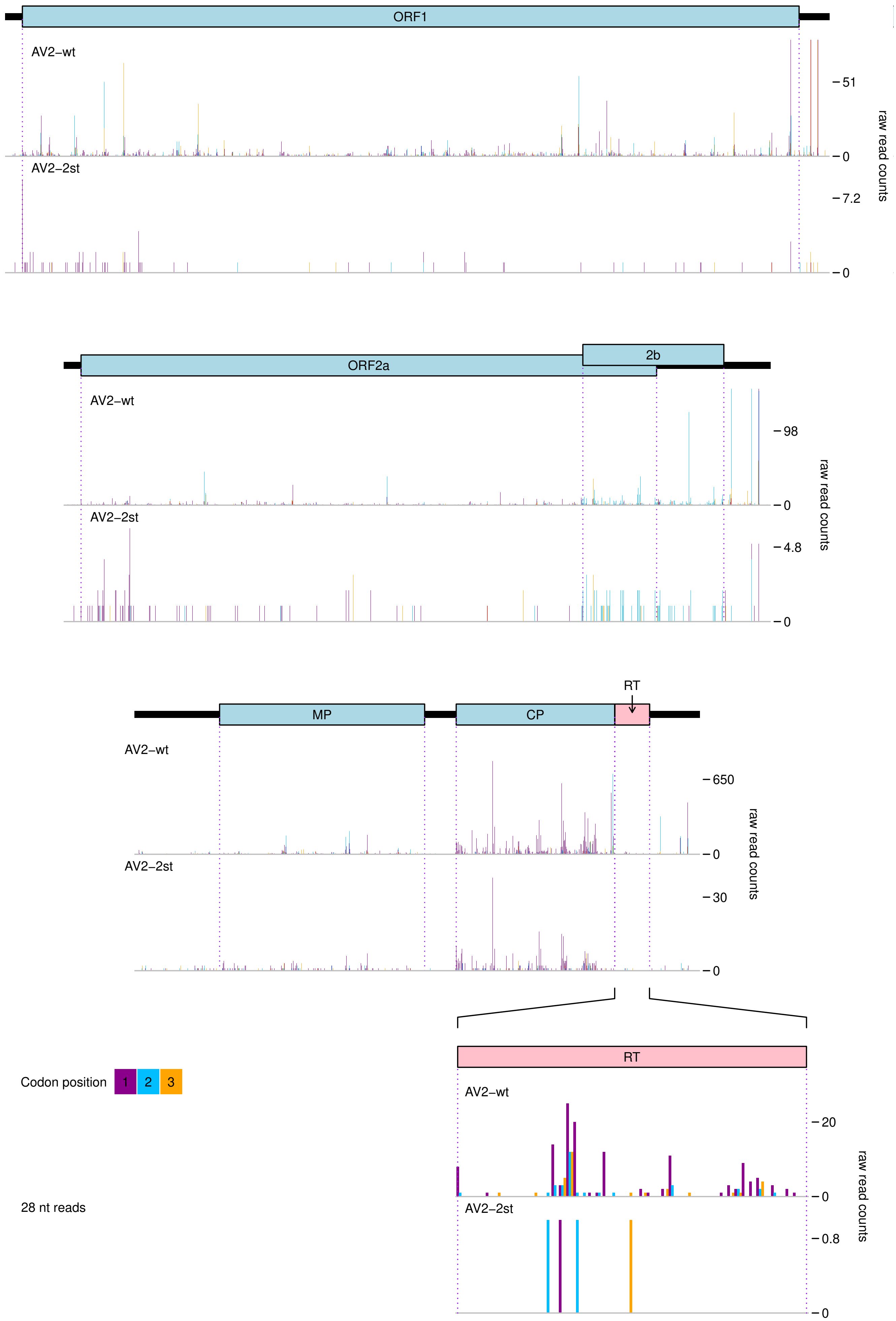
ORF1
AV2−wt
AV2−2st
51
0
7.2
0
raw read counts
ORF2a
2b
AV2−wt
AV2−2st
98
0
4.8
0
raw read counts
RT
MP
CP
AV2−wt
AV2−2st
650
0
30
0
raw read counts
RT
AV2−wt
AV2−2st
20
0
0.8
0
raw read counts
Codon position 1 2 3
28 nt reads

Supplementary Figure M. Detection of AV2 CP by western blot in plants infected with AV2. Samples were collected from the upper leaves at 47 dpi. Sizes of molecular weight markers are indicated on the right. Ponceau red staining of the large Rubisco subunit (RbcL) was used as a loading control.

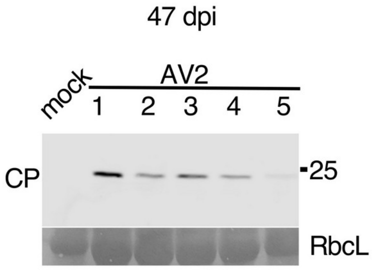

Supplementary Figure N. Detection of CP by western blot in wild type *N. benthamiana* (upper panels) and RDR6i *N. benthamiana* (lower panels) infected by AV2, AV2-2st, AV2-mutZF, AV2-st2b, AV2-st2b-2st or AV2-st2b-mutZF. Samples were collected from the 2nd non-inoculated leaf at 7 dpi and from the 3rd non-inoculated leaf at 14 dpi. Sizes of molecular weight markers are indicated on the right. Ponceau red staining of the large Rubisco subunit (RbcL) was used as a loading control.

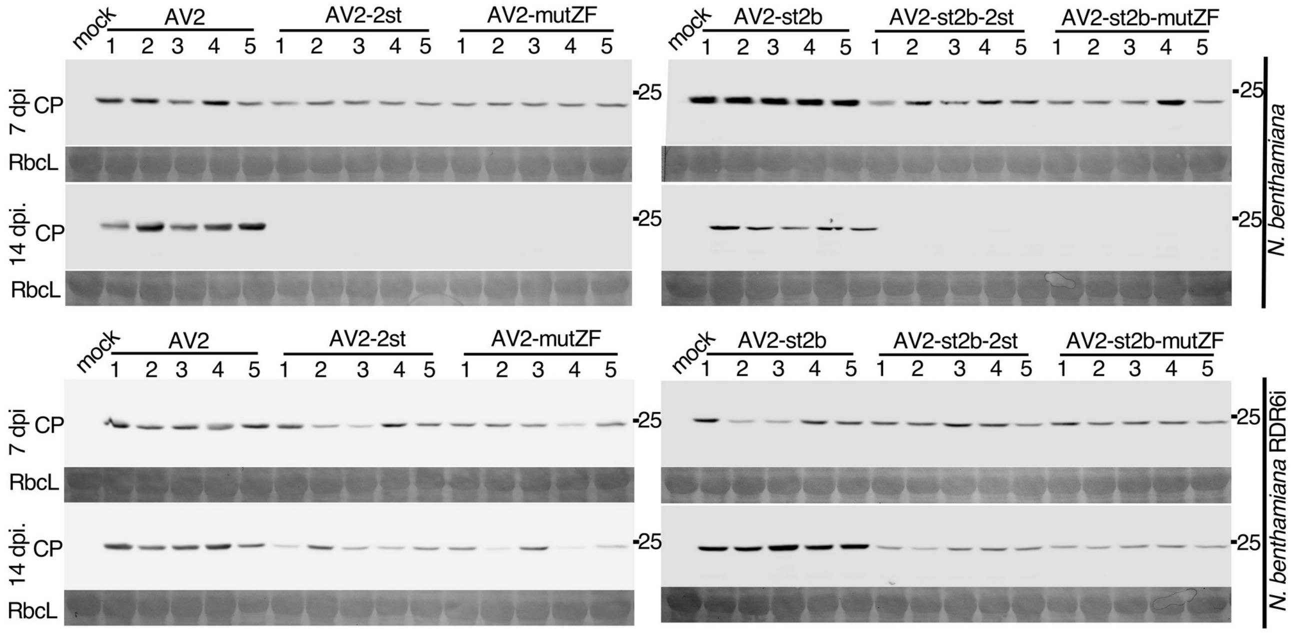

Supplementary Figure O. The number of 22 nt, 24 nt and all reads mapping to each RNA, divided by the number of virus mapping reads in the replicate, for each replicate, time point and treatment, for AV2 and AV2-2st infected samples. RNA1 is shown in black and at the bottom of each bar, RNA2 in cyan and in the centre, RNA3 in orange and at the top.

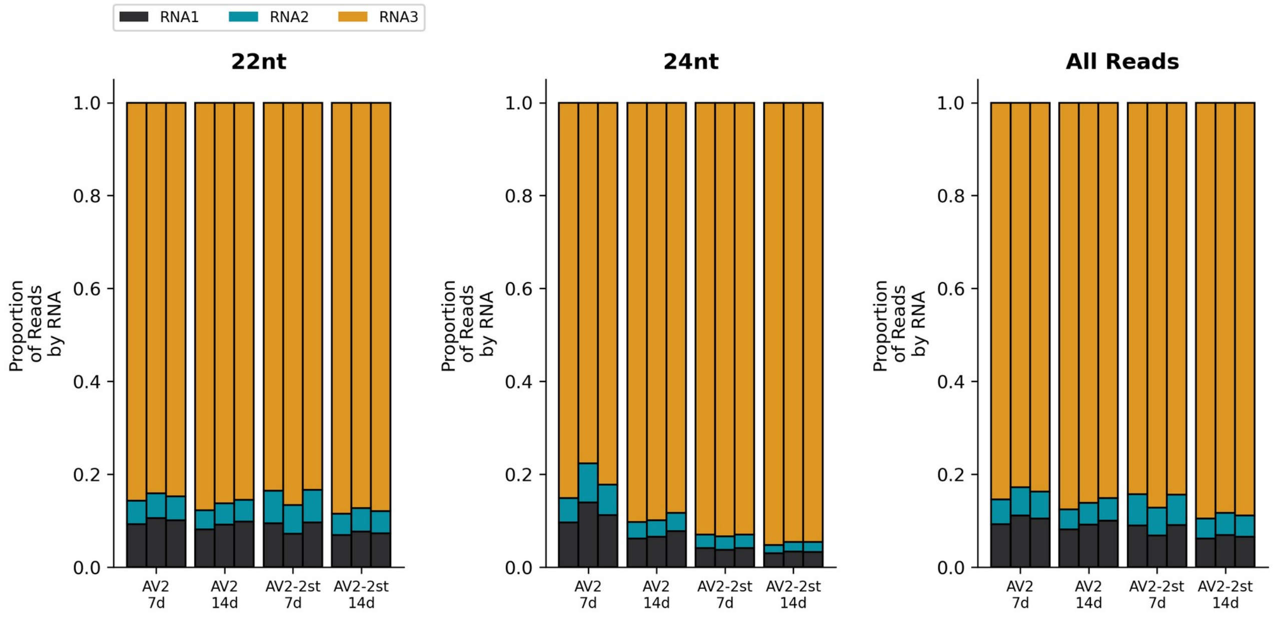

Supplementary Figure P. (i) The proportion of 21 nt, 22 nt, 24 nt and all reads which mapped to the host (bottom section of each bar, light colours), virus (middle section, dark colours) and was unmapped for each sample (top section, grey), separated by treatment, time point and replicate. **(ii)** The raw number of reads mapping to the each strand of the host and the virus, plus the number of unmapped reads (unstranded but shown as "+") for each replicate, treatment and time point.

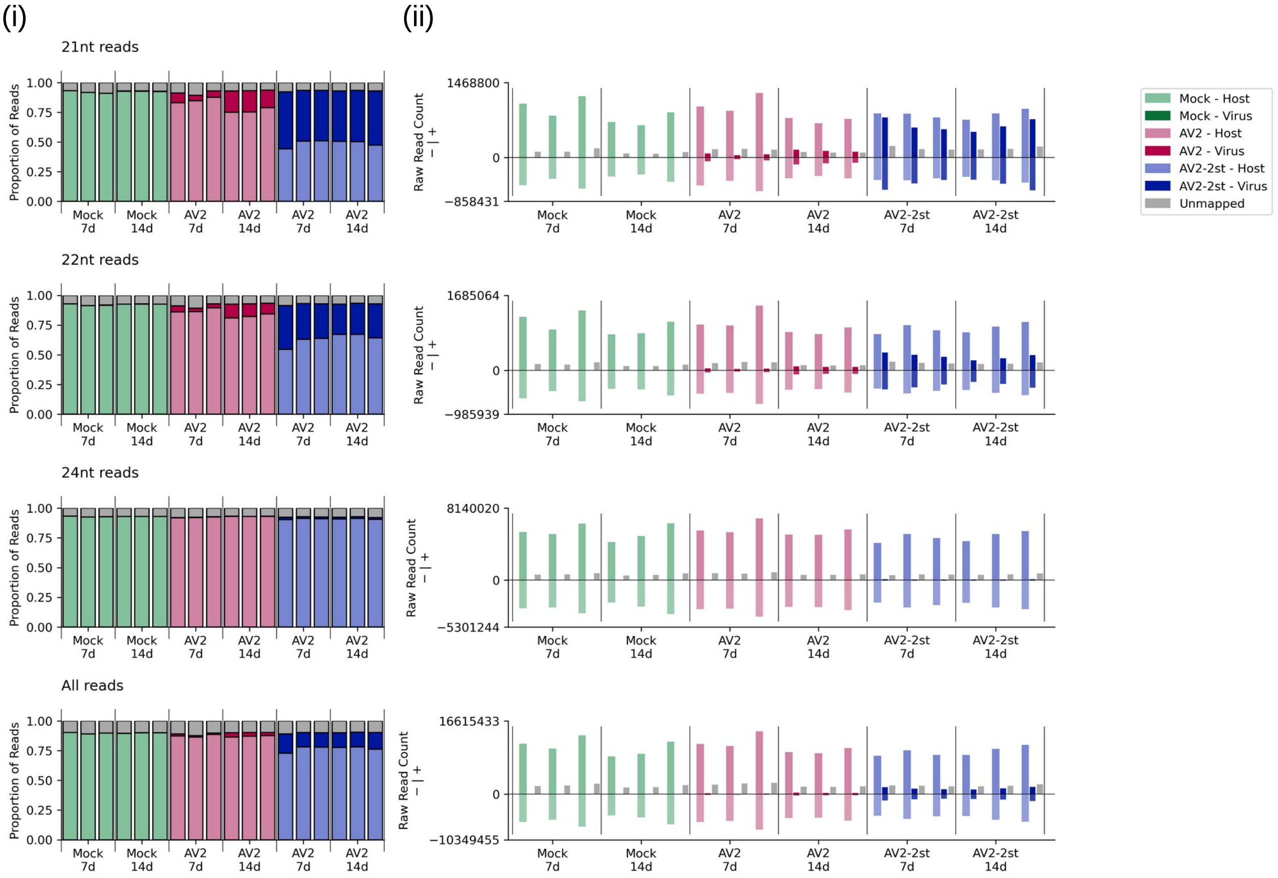

Supplementary Figure Q. The number of virus mapped 21 nt, 22 nt, 24 nt and all reads, with each possible 5′-terminal nucleotide for AV2 and AV2-2st infected samples, for each timepoint, divided by the total number of virus mapped reads for the corresponding read length, sample and timepoint.

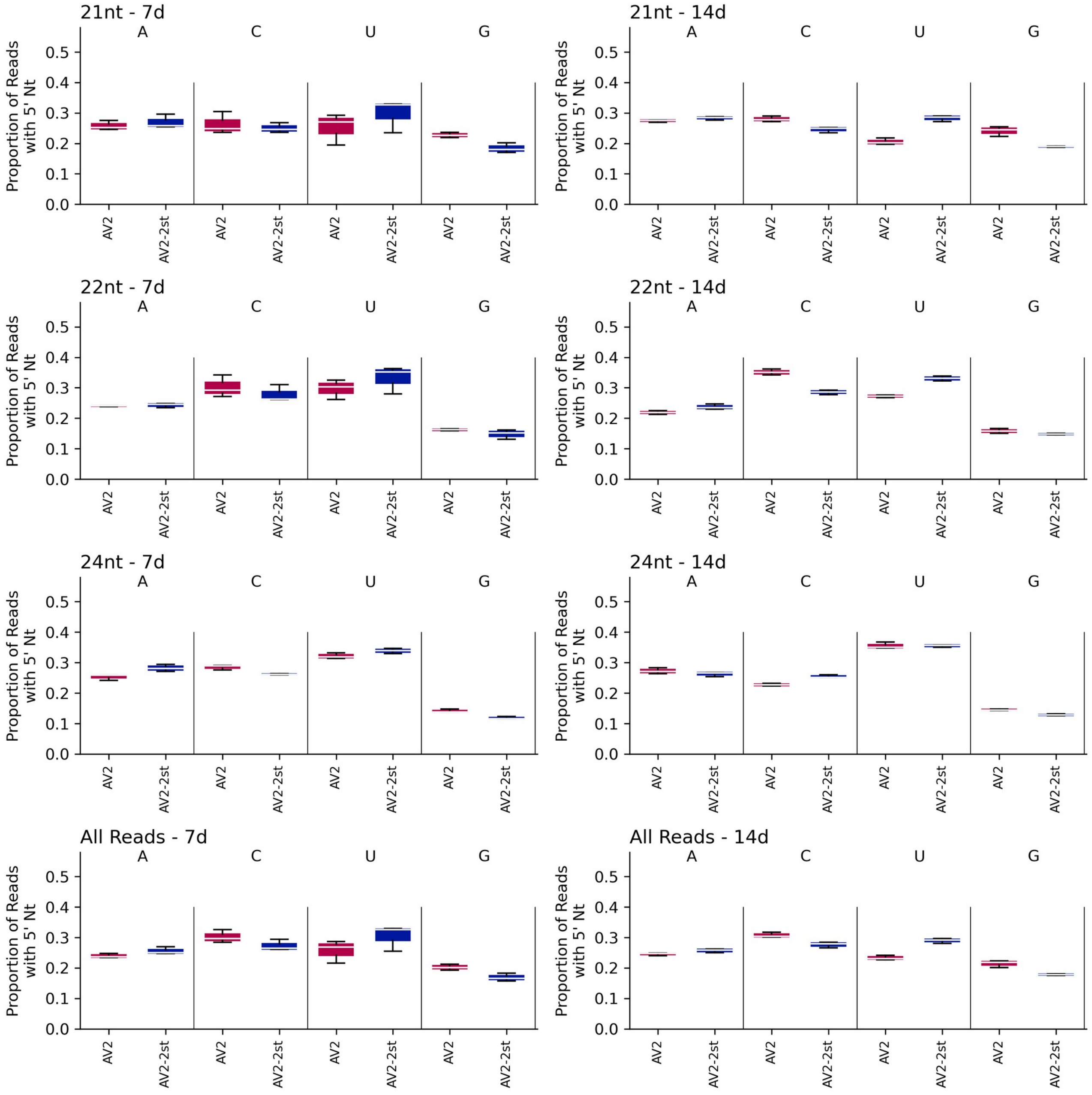

Supplementary Figure R. The normalised number of 22 nt, 24 nt and all reads (shown as one line per replicate, left *y* axis) and the proportion of all reads of this length mapping to this RNA (shaded and summed across replicates, right *y* axis) spanning each position in the viral genome across RNA1 (left), RNA2 (middle) and RNA3 (right). Both annotations are smoothed with a 100 nt sliding window. AV2 infected samples are shown in red and AV2-2st infected samples in blue. Positive-sense reads are shown above the axis and negative-sense reads are shown below the axis. Note, reads that map to the conserved 3′ regions may be assigned at random to one of the three segments, potentially leading to an apparent over-abundance in RNAs 1 and 2 due to mismapping of reads that actually derive from the more abundant RNA3. **(i)** 22 nt reads, 7 dpi. **(ii)** 22 nt reads, 14 dpi. **(iii)** 24 nt reads, 7 dpi. **(iv)** 24 nt reads, 14 dpi. **(v)** All reads, 7 dpi. **(vi)** All reads, 14 dpi.

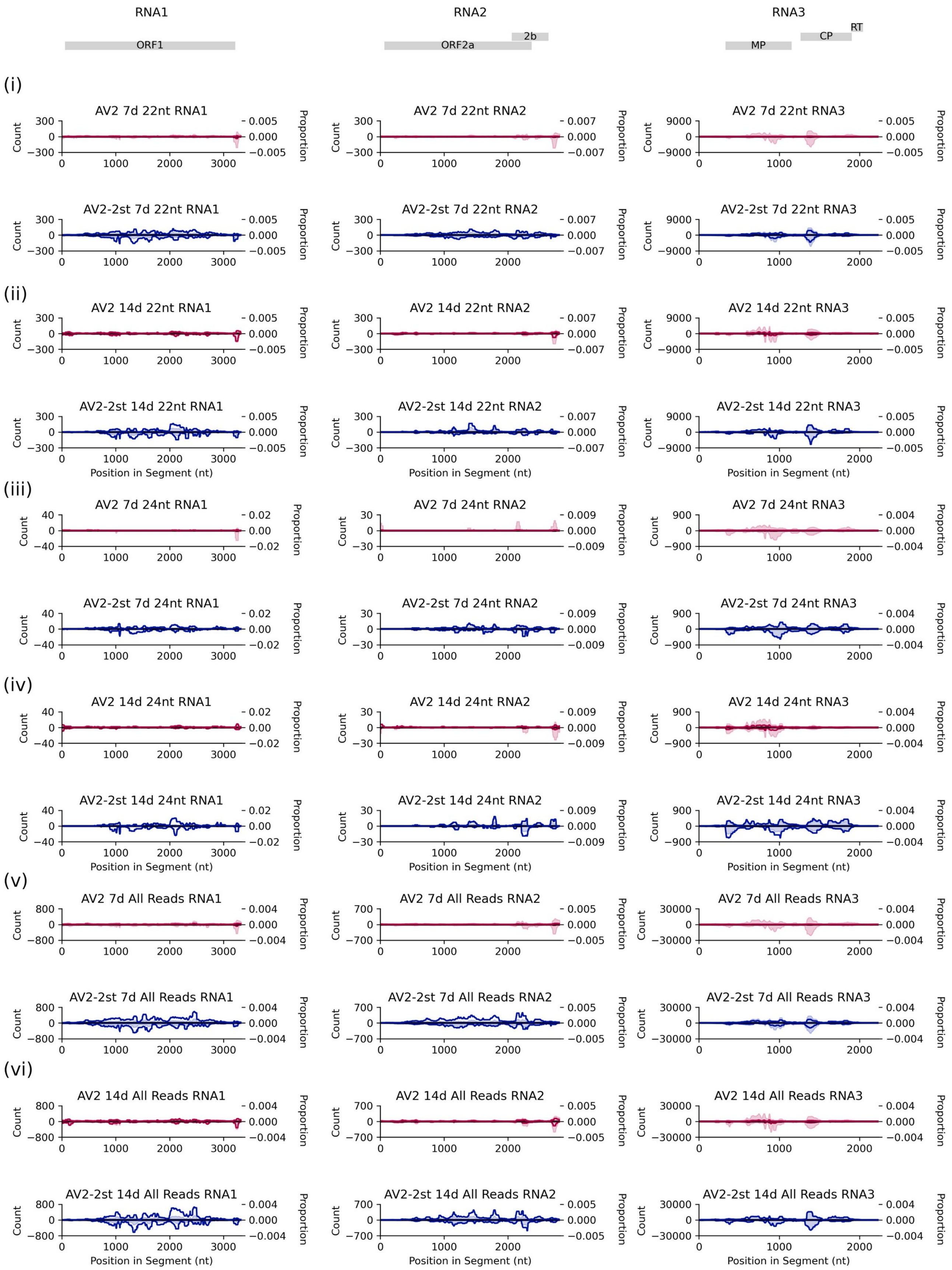

RNA1
ORF1
RNA2
2b
ORF2a
RNA3
RT
CP
MP
(i)
AV2 7d 22nt RNA1
AV2 7d 22nt RNA2
AV2 7d 22nt RNA3
AV2-2st 7d 22nt RNA1
AV2-2st 7d 22nt RNA2
AV2-2st 7d 22nt RNA3
(ii)
AV2 14d 22nt RNA1
AV2 14d 22nt RNA2
AV2 14d 22nt RNA3
AV2-2st 14d 22nt RNA1
AV2-2st 14d 22nt RNA2
AV2-2st 14d 22nt RNA3
(iii)
AV2 7d 24nt RNA1
AV2 7d 24nt RNA2
AV2 7d 24nt RNA3
AV2-2st 7d 24nt RNA1
AV2-2st 7d 24nt RNA2
AV2-2st 7d 24nt RNA3
(iv)
AV2 14d 24nt RNA1
AV2 14d 24nt RNA2
AV2 14d 24nt RNA3
AV2-2st 14d 24nt RNA1
AV2-2st 14d 24nt RNA2
AV2-2st 14d 24nt RNA3
(v)
AV2 7d All Reads RNA1
AV2 7d All Reads RNA2
AV2 7d All Reads RNA3
AV2-2st 7d All Reads RNA1
AV2-2st 7d All Reads RNA2
AV2-2st 7d All Reads RNA3
(vi)
AV2 14d All Reads RNA1
AV2 14d All Reads RNA2
AV2 14d All Reads RNA3
AV2-2st 14d All Reads RNA1
AV2-2st 14d All Reads RNA2
AV2-2st 14d All Reads RNA3
Count
Proportion
Position in Segment (nt)

Supplementary Figure S. The number of 21 nt reads mapping to miRNAs in our “curated” miRNA set, normalised as reads per million host mapped reads, for mock, AV2 and AV2-2st infected samples, for each treatment, time point and replicate.

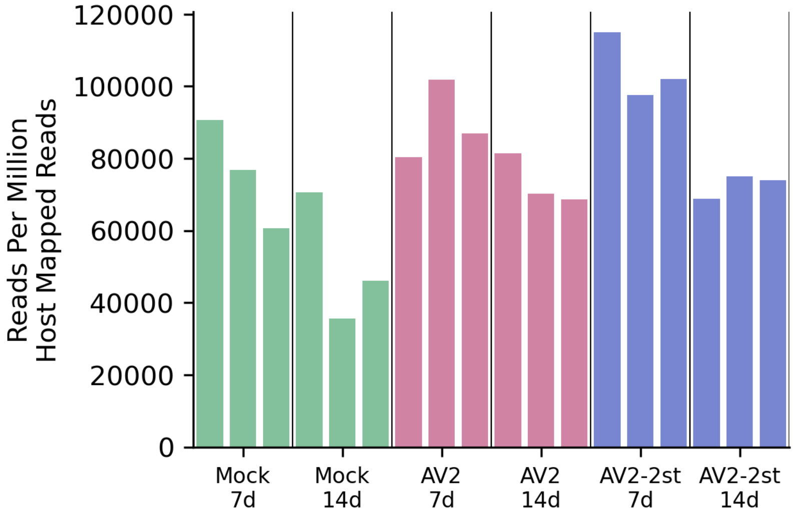

Supplementary Data A

Delineation of terminal regions conserved between segments in asparagus virus 2.

- AUG — Start codons of ORFs 1 and 2a.
- UNN — Stop codons of ORFs 1, 2b and CP-RT.
- **AUGC** — Conserved AUGC repeats in the 3′UTR (see Ref. [6]).
- (( )) — Potential RNA stem-loop structures. Note that the AUGC elements tend to be positioned in predicted single-stranded regions between consecutive stem-loops.
- *** — Conserved residues.
- U — Note that the terminal U in the RNA3 3′UTR is present in our AV2 infectious clone but is lost during infection so is likely not present in natural isolates.
- GUGU — Start of the 3′ regions largely conserved between RNAs 1, 2 and 3.

5'UTR:

```
RNA1            GUAUUGUGCAGUAUUAUUUUACGCACGACCCGGAUUAAUCAGUUUUCUGAGUAUAUACCC
RNA2            GUAUUGUGCAGUAUUAUUUUACGCACGACCCGGAUUAAUCAGUUUUCUGAGUAUAUACCC
                ************************************************************

RNA1            GAUAGAAACUCAAUGGAUAC
RNA2            GAUAGAAACUCAAUGGAUGC
                ****************** *
```

3'UTR:

```
RNA2            UGGUGACAGAAUUUCUGAUCGAGUACCAUCUCCAUG-GUAUCUCG---------------
RNA1            UGGU--CUAAAACUUUAGACGAACAAGAGCUGUCCGCGUAUAGGGCA-------------
RNA3            UGUGAAUGUUAUGACAAUGCUUGCAACACC-UUGUG-GUGUUCUGCAAAUGUCUUUUGUU
                **        *        *    *  * *     * ** *   *

                                        ORF2b                  ((((  (((
RNA2            -------------------GUCAAUGACGUGUUUCUUUCAUGAAACCUAGUGAAGGGUCC
RNA1            -------------------GCUAAU--AGUGUUUUUCCAUGAAACCUAGUGAAGGGUCC
RNA3            UCCACGAACCUUAAUGGUUAGUGAUGAAGUGUUUCUUUCAUGAAACCUAGUGAAGGGUCC
                                       **   ****** ** **********************
                           CP-RT
                                              ORF1
                        ((((   ))))    )))  ))))           (((((
RNA2            UCCGGGAUGUGCUCAGCACCUAGCCUAAGCUAAGUCCAUAUGCCCAUCUUCGCUGCUCCG
RNA1            UCCGGGAUGUGCUCAGCACCUAGCCUAAGCUAAGUCCAUAUGCCCAUCUUUGCUGCUCCG
RNA3            UCCGGGAUGUGCUCAGCACCUAGCCUAAGCUAAGUCCAUAUGCCCAUCUUUGCUGCUCCG
                ************************************************** *********

                )))))                       (((((((      )))))))    (((( (((
RNA2            GAUGGAUGUUUAUACCCGCUAUGGAUGCCUAUUACUGAAAUGUAAUAGAUGCCUAAUACU
RNA1            GAUGGAUGUUUAUACCCGCUAUGGAUGCCUAUUACUGAAAUGUAAUAGAUGCCUAAUACU
RNA3            GAUGGAUGUUUAUACCCGCUAUGGAUGCCUAUUACUGGAAUGUAAUAGAUGCCUAAUACU
                ************************************* **********************

                (((((     ))))))))))))    ((((   ))))
RNA2            CUCUCUCAGGGAGAGAGUUUAGAUGCCUCCAAAGGAGAUGC-
RNA1            CUCUCUCAUGGAGAGAGUUUAGAUGCCUCCAAAGGAGAUGC-
RNA3            CUCUCUCAUGGAGAGAGUUUAGAUGCCUCCAAAGGAGAUGCU
                ******** ********************************
```

Supplementary Data B

Sequence alignments of asparagus virus 2 with closely related reference sequences, showing the intergenic and 3′UTR regions of RNA3. The following sequences were chosen as comprising the immediate clade containing the asparagus virus 2 reference sequence (see Supplementary Figure B): OL472059, NC_003546, NC_003835, NC_039076, NC_003810, NC_011807, NC_009536 and NC_003570. Full-length RNA3 sequences were aligned with MUSCLE [7] with default parameters and regions encompassing the IGR and RT domain plus 3′UTR were excised; very minor manual alignment adjustments were made to align predicted base-pairings in the annotated potential 3′ stem-loops.

AUG	CP start codon.
UNN	MP, CP, RT stop codons.
**AUGC**	Conserved AUGC repeats in the 3′UTR (see Ref. [6]).
(( ))	Potential RNA stem-loop structures in the 3′UTR. Constituent nucleotides are highlighted in yellow (N), with additional colours tan (N) and pink (N) to highlight compensatory substitutions, and cyan (N) to mark consistent substitutions involving a G:U base-pairing.
***	Conserved residues.
GUGU	Start of the 3′ regions largely conserved between RNAs 1, 2 and 3, based on the AV2 sequences (see Supplementary Data A).
G	5′ end of the AV2 sgRNA4 as determined by RACE-PCR.

Intergenic and flanking regions

```
                                         MP stop
OL472059        CGCAA--ACAUAAUACCCCGAAUCCAUGAUGUAU-AAGUGUCGUGGGAUGGUGUUCCCUA
NC_003546       GGCGUGGACAGAAUACCCCGACUCCGUGAUGUAU-AUGCGUUGUGGGAUGGAUACCCUUA
NC_003835       GGCAGGGACAGAAUACCCCGACUCCGUGAUUUAU-GUGU-UCGUGGGAUGGAUACCCUUA
NC_039076       GGCAGGGGCAGAAUACCCCGAAUCCGUGAUUUAUACAGUGUUGUGGGAUGGAUACCCUUA
NC_009536       CGCAAUCUCACAAUACUCCGAAGUGAUAAGUUAUUAAGUUUCGUGGGAUGGAUACCCUUA
NC_003810       CGCAACCUCAAAACGCUCCAUAGUGAUGAGUUAUAAAGUUACGUGGGAUGGAUACCCUGA
NC_003570       CGCAAUCUCAUAACACUCCAUAGUGAUGAGUUAU-UAGUUUCGUGGGAUGGAUACCCUUA
NC_011807       CGCAGUCUCGAAAUACUCCGUAGUGGUGAGUUAUAAAGUCUCGUGGGAUGGAUACCCUUA
                 **     *  **  * **       * *  ***    *    *********    **  *

OL472059        UCAAGGACCCCCCAGAUACUCAUCG--UGUGGGGGUUUGCGUAUUUGAGAAGUGUUUUCC
NC_003546       -UUUGGGUCCCCCAGACGCGCGUUG--CGUGGGGGUUCAUGUCUUG-AGAAGCCUUUUCC
NC_003835       -UUUGGGUCCCCCGGUCGCAGGUUG--CGUGGGGGUCUUUGUCUUGAAGAAGCCUUUUCC
NC_039076       UUUUGGGUCCCCCGGACGCGGGUCG--CGUGGGGGUUCUUGUCUUG-AGAAGCCUUUUCC
NC_009536       -UUUGGGUCCCCCCGAGACUGCUUGUCCGGGAGGAGAUUUGUCUUG---AAGUCUCCUUC
NC_003810       -UUUGGGUCCCCCCGUGACUGCUUGUCCGGGAGGAGGU-UGUCUUG--GGAGUCUCCUUC
NC_003570       -UUUGGGUCCCCCCGGAGCUGCUCGUCCAGGAGGAGAUUUGUCUUG---AAGUUUCCUUC
NC_011807       -UUUGGGUCCCCCCGGGACUGCUUGUCCGGGAGGAGAUUUGUCUUG---AAGUUUCCUUC
                    **  ***** *   *   * *     * **      ** **     **  *  * *

                                                            CP start
OL472059        UCAGUGAUUAUUU--GCGUCCC--AUUCUACCAC--------UAUGGCUACUAAUAACAA
NC_003546       UC------UACUA-AGCGUUCC-----AUCCAACCACUGAUCCAUGGCAAAUAGAAGCAA
NC_003835       UG------UACUU-UCCGUCCCAUUUUC-------AGCACAACAUGUCUAAUUUUAGUAA
NC_039076       UC------UACUU-UCCGUCCC--UUUUUCACAC----ACAACAUGUCUAAUACUAGCAA
NC_009536       UC------CACUUUUACGUCCC--UUUCUCCCAUAACAACAAAAUGUC------UGGUAA
NC_003810       UU------UACCGAAACGUUAUCUAUAAUUCUUCCUAACAACAAUGUC------UGGCAA
NC_003570       UC------CACCU-UGCGUCAUCUUUUCUCCUA--ACAACAACAUGUC------UGGUAA
NC_011807       UC------CACUU-UCCGUCACCUAUUCUUCCACUACAACAACAUGUC------UGGUAA
                *        *      ***                        *** *          **

OL472059        CUGUCCUGUUACUCUGAAUGGUCGAACCUACGUUCCCGCUGG---UAACAAUGCCCCUGC
NC_003546       ---UGCUAUCGAAGUCAACGGUGUGUGGUACAACCGAGC------CGAUAAUGCCCCCGU
NC_003835       ---UGCUAUCGAGGUGAAUGGUAAAUGGUAUUCACCUGU---------UAAUGCCCCGCC
NC_039076       ---UGCUAUUGAGGUGAAUGGUAAAUGGUACUCACCUAU------GAAUAAUGCCCCCGC
NC_009536       ---UGCUAUUGAAAUUAAUGGUCGCUGGUAUCAACCGGCACC---CAAUAGUGCCCCGAC
NC_003810       ---UGCUAUUGAAGUUAAUGGCCAGUGGUAUUCACCGGUUACUAACAAUAAUGCCCCGUC
NC_003570       ---UGCUAUUGAAGUUAAUGGUCGUUGGUAUCAACCAGCACC---CAAUAAUGCCCCGAC
NC_011807       ---UGCUAUUGAAGUUAAUGGUCGUUGGUAUACUCCAGCACC---CAAUAAUGCCCCGAC
                   * ** *     * ** **       **    *              * ******
```

Read through domain and 3'UTR

```
                                                                    CP stop
OL472059        UGCCUGGGUUGAAACCACCACCGAAUUUCUUAGUAUGCGAG---------GAAUGACCAC
NC_003546       UACCUGGUAUUAAGCCUCCGCCUAAUUUCCUGGUAUGUGAGAUGGAUGAUAAAUGACCAC
NC_003835       UUCCUGGUGUGAAGCCUCCAGCAAACUUUUUAGUUUGUGAG---------GAGUGACUAC
NC_039076       UACCUGGUGUGAAGCCUCCCUCAAACUUUUUGGUUUGUGAG---------GAGUGACCAC
NC_009536       UUCCUGGUGUGAAGCCACCAAGCAAUUUCUUGGUUGUUGAA---------GAAUGACUAC
NC_003810       UACCGGGAGUGAAGCCACCAAGUAAUUUCUUGGUGUGUGAG---------GAUUGACUAC
NC_003570       UUCCAGGCGUGAAGCCACCAGCAAAUUUUCUGGUUGUUGAG---------GAAUGACUAC
NC_011807       UACCCGGUGUGAAGCCACCAAGUAAUUUCUUGGUUGUUGAG---------GAAUGACCAC
                * ** **  * ** ** **    ** **  * **    **           * **** **

OL472059        GCUCGGGG-------------------------UGUGAAUCGUGUGGUUAUGUACCAU
NC_003546       A---AGAU-------------------------UGUGAAUCUUGUGGUUAUAUUCCAU
NC_003835       ACUCGGGCUUU----------------------UGUCCCGUGUGUGGUUAUGUACCAC
NC_039076       GCUCGGGCUUA----------------------UGCCAGAGUUGUGGAUUUAUUCCAU
NC_009536       ACUCGGACGAU----------------------UGUCCGAGUCAAAGUGUCAUCCCUU
NC_003810       ACUCGGACGAU----------------------UGUUCGAGUAGAAAUGUCAUUCCUU
NC_003570       ACUCGGGUGAU------UACCUCCAUUCUGUUGAAUGUGAGAGGAAGUGUGUGAUUCCAU
NC_011807       GCUCGGGUGAGUGUGUAUACCUCCAUUCUAAUGAAUGUGAGAAGAAAAAUGUGAUUCCAU
                     *                             **                 * **

OL472059        AUUGCCAUUGUAAAUGCUACAAUGAUGCCUGCUCAGUGGUUGGUUGUGAGCAGAAUAUCU
NC_003546       ACUGCCACUGUGAAUGUUGGCCGUACAGAUGCUACACCAUUGGGUGUAAUGCUAAUGAUC
NC_003835       UUUGUCAUUGUAUGUGCUACAAUUACUCAUGUGAUGUCAUUGGUUGUGAUGCCAAUGAUA
NC_039076       AUUGUCAUUGUUAUUGUUGCGAUGACGUAUGUGAUGUCAUUGGAUGUUUAGCCAAUGAUA
NC_009536       UCUGUCAUUGCGAAUGUUAUGACAAUGCUUGCGGUACACCUUGGUGUCCUGCUAAUGCCA
NC_003810       UUUGCCAUUGCUCAUGUUGCCCUUUUCACUGUGAUGUGAUAGGAUGUGUGUGCAAUGAUG
NC_003570       UCUGUCAUUGCGUGUGCUAUGACGAUGCUUGCGAUGUUCUUUGGUGUUCCCCAAAUGAUU
NC_011807       UCUGUCAUUGUGAAUGUUAUGACAAUGCUUGCAACACCUUGUGGUGUCCUGCAAAUGUCU
                  ** ** **    ** *           **           * ***      ***

                                             RT stop
                                                                       ((((
OL472059        GGUUUAUACCAGAUG-------------------AAGUGUUUCCAUA--GGAACCUUAUC
NC_003546       UAUGGUUUAUAGAUUCUUA---------------AAGUCUCACUUUCGUG--AGCUGAUG
NC_003835       CCUUCUGUAUAGAAGAUGAUC-------------AAGUGUAACAUUCAUGUUACCUAGCG
NC_039076       CCGUCUAUGACG----------------------AAGUGUAACAUUCAUGUUACCUAGUG
NC_009536       AUUGUUUCCAUGAAUUUCAUGAAAAUUAUUGAUGACGUGUU-CUUUCAUAGAAUCUAGCA
NC_003810       CCUUCUUUUACGAAGAU-----------------UAGUGUU-CCUUCAUGGAACCUGGUA
NC_003570       UAUGUUACCACGAGUCUAAUGACUGGUGAUGU--UAGUGUUUCUUUCAUGAAACCUAGUG
NC_011807       UUUGUUUCCACGAACCUUA--AUGGUUAGUGAUGAAGUGUUUCUUUCAUGAAACCUAGUG
                           *                        ** *  *  *      * **

                 (((            ((((   ))))    )))  ))))           (((((
OL472059        AGACGUCUUCAGGGAUGUACUAAGUACCUCGGUCUGAUGAAAUCUAUAUGCCUAGU--CG
NC_003546       AAGGGCCCUCCGGGGUGUGCUCAGCACUUAACCUAAGUCAAGUUCAUAUGCCCACCUUUG
NC_003835       AAGGGUCCUCCGGGAUGUGCUCAGCACUCAGCCUUAGCUAAGUUCAUAUGCCCACCUUUG
NC_039076       AAGGGUCCUCCGGGAUGUGCUCAGCACCUAGCCUAAGCUAAGUCCAUAUGCCCACCUUUG
NC_009536       AGGAGCCCUCAGGGGUGCACUCAGUGCCUAGUCUACGCUAAGUCUAUAUGCCCACCUUUG
NC_003810       AGAGGUCCUCCGGGAUGUGCUCAGCACCUAGCUUAAGCCAAGUCCAUAUGCCCACCUUCG
NC_003570       AAGGAUCCUCCGGGAUGUGCUCAGCACCUAGCCUAAACUAAGUCCAUAUGCCCAUCUUUG
NC_011807       AAGGGU-CUCCGGGAUK-GCUCAGCACCUAGCCUAAGCUAAGUCCAUAUGCCCAUCUUUG
                *       ** *** *   ** **  *            ** *  ******* *   * *

                         )))))                           (((((((      ))))))
OL472059        CCU-CUACAACUAGAGUCCU----------C-UAUAGAUGCCCCAUACUGUAAUGUUUGG
NC_003546       CUG-CUCCGGGUGGAUGCUUCGUGCAAAUGC-UAUGAAUGCCUACGUA--UAUGUGCGUA
NC_003835       CUGUCUCCGGGUGGAUGCCU---CAUGGUGC-UAUGGAUGC-------------------
NC_039076       CUA-CUCCGGGUGGAAGCCU-AUACAAAUGC-UAUGGAUGCCUAUAUUUGAAAUAAUAUA
NC_009536       CUA-CUCCGGGUGGAUGUUC----UAAGUGUAUAUAGAUGCCUAUAUUUGAAAUAAUAUA
NC_003810       CUG-CUCCGGGUGGAUGCUU---AUACAUGC-UAUGGAUGCCUACAUUUGAAAUAAUGUA
NC_003570       CUG-CUCCGGAUGGAUGUUA---AUACCCCC-UAUGGAUGCCUAUUAUUGAAAAAUAAUA
NC_011807       CUG-CUCCGGAUGGAUGUUU---AUACCCGC-UAUGGAUGCCUAUUACUGAAAUGUAAUA
                *   ** *   * **                 ***  ****

                )    (( ((((((((((     )))))))))) ))    ((((       ))))
OL472059        GAUGCCA-AAUCUCUCUCUCUU-GAGAGAGAUU-UGAUGCCUACCUUUGGUAGAGGCGAC
NC_003546       GAUGCCU-AUAUUUUCUCUCCU-GAGAAAAUAU-AGAUGCCUCC----AAAGGAGAUGC-
NC_003835       ------------------------------------------------------------
NC_039076       GAUGCCUAAAUUUUUCUCUCAUGGAGAAAAAUUUAGAUGCCUCC----AAAGGAGAUGC-
NC_009536       GAUGCCCAA-ACUCUCUCUCAUGGAGAGAGAAU-GGAUGCCUCC----GAAGGAGAUGC-
NC_003810       GAUGCCUAAUACUCUCUCUCAGGGAGAGAGUUU-AGAUGCCUCC----AAAGGAGAUGC-
NC_003570       GAUGCCUAAUUCUCUCUCUCAGGGAGAGAGAUU-AGAUGCCUCC-----AAGGAGAUGC-
NC_011807       GAUGCCUAAUACUCUCUCUCAGGGAGAGAGUUU-AGAUGCCUCC----AAAGGAGAUGCU
```

Supplementary Data C

Identification and annotation of putative readthrough (RT) domains in different ilarvirus clades. Ilarvirus clusters were defined by applying BLASTCLUST [8] to CP amino acid sequences with an 80% identity threshold, as described in Methods. Clusters are ordered by the number of component sequences, from most to fewest. Subsequently, clusters 8 and 17 were each split into two subclusters (8a, 8b, 17a, 17b) due to the presence of two NCBI RefSeqs in each of the initial clusters. Cluster 6 was split into two subclusters (6a, 6b) because sequences in this cluster appeared to divide into two groups – those with or without an insert in the RNA3 3′UTR. Cluster 5 was split into two subclusters because the single Fragaria chiloensis latent virus sequence (cluster 5a) contained a putative RT domain with a predicted zinc-finger motif, whereas the potato yellowing virus sequences (cluster 5b) had a shorter putative RT domain without a predicted zinc-finger motif. For each cluster or subcluster, the following pages present: (a) an intersegment alignment of the 3′UTR sequences of reference RNA 1, 2 and 3 sequences (where RNA1/RNA2 3′UTR sequences are available); and (b) for all available RNA3 sequences in the cluster with 3′UTR coverage (see Methods), the context of the CP stop codon, and the amino acid translation of the ORF in-frame with and immediately 3′ of the CP stop codon whether or not readthrough is proposed to occur in that cluster.

Alignments of the RNA 1, 2 and 3 3′UTRs were performed with MUSCLE [7] with default parameters. Note that the 3′UTRs are often 3′-incomplete. Where the 3′ truncation point differs between segments, this sometimes leads to poor quality/misleading alignments. In this study, the presence of a lengthy insert (or inserts) in RNA3 when the 3′-of-CP region was compared with the 3′UTRs of RNAs 1 and 2, was taken as a proxy for the putative presence of a RT domain in that cluster.

A few sequences in clusters proposed to encode a RT domain can be observed to have atypical (truncated or extended) RT ORFs. These may be due to sequencing errors or sequencing of defective viral genomes. Whereas insertion/deletion sequencing errors that occur in known coding ORFs are generally corrected before submission of sequences to NCBI, insertion/deletion errors that occur outside of known ORFs often escape notice (examples in Refs [9] and [10]).

Note that shortened RT peptides can result from early termination codons or from sequences with incomplete 3′UTR coverage. In the peptide sequences presented, an "*" indicates a termination codon. Shorter-than-normal RT peptides that do not end in an "*" are shortened because the RT ORF runs off the end of the available RNA3 nucleotide sequence.

UNN CP stop codon in RNA3

UNN Next in-frame stop codon in RNA3

CLUSTER 1

Alignment of 3'UTRs from the 3 segments of a representative genome

```
RNA3            UGACUAGAUGGUCACCUCGGUGAGACCGAGAUGCCGCACAUAGAUGGUAAUUCCUGUGUG
RNA1            U----------------------------GAUACAAUAUGUUUAAUGUACUCCCU-----
RNA2            U----------------------------GAUUUAG----------GUAUUUCCU-----
                *                            ***              *** * ***

RNA3            CGAUGUGGAUUCACACCUCGACGUAUUGUAGAUCAAUACAUUUGGAAGUGUGACAUCUGU
RNA1            -------------------------CUAUAGA------------GAGUUGCCGCAGAUUU
RNA2            ----------------------CUCUUAUAGA------------GAGAUGCCGCUU----
                                          * ****            **  **   *

RNA3            UCCUAUGAACAGUAUGCAUAGCUGCCGCGCAUCCGGGCGUGAGACUAUGAUCCAUGUGAG
RNA1            UC---UGCGAAGU-UUCGGAACUGUAUUUUGCCGGUGGAUAGG-----------------
RNA2            -----UGCGAAGA----------------UGUCAGUAGGUGGG-----------------
                     **   **                    * *    *  *

RNA3            CAUUCCGAAUCGGACGAUUUCCAUGGGAAACCAUGAGACCUUUGGGUCGAUGCUUACGUU
RNA1            -----CGGGUGUAGUGCUUCU---------CCAACACACUUCCA---------------C
RNA2            -----CGAAACAUACGCUUCU---------UUAUGUUUUCUACUGAU-------------
                     **        * **             *       *

RNA3            UGGUGCCAGUAGUAUAUAAU-AUACUACUGAUGCCUCCUUUAUAGGAGAUGC
RNA1            UGAUGCUGUUUAUAUCUAAUGAUAUAAACAAUGCCUCCUUUAAAGGAGAUGC
RNA2            --AUUCCAGUUAUAUCUAAUGAUAUAACUGAUGCCUCC--AAAUGGAGAUGC
                   * *   *  *** **** ***  *   ********   *  ********
```

RNA1 NC_003844 172 nt
RNA2 NC_003842 142 nt
RNA3 NC_003845 291 nt

Note: Unlike for many ilarvirus genomes (see other clusters below), here the 3'UTRs are not so highly conserved between RNAs 1, 2 and 3 and so the insert of the RT region is less clear in the alignment. Nonetheless, the difference in length between the RNA3 and the RNA1 and RNA2 sequences shown (291 − 0.5 x (172 + 142) = 134 nt) is close to the length of the RT sequence (138 nt), consistent with Cluster 1 sequences encoding a RT domain in their RNA3s.

All sequences in the cluster

```
    104 Tobacco streak virus
      5 Sunflower necrosis virus (JF340380 JF340381 JF340382 JF340383 JF340384)
```

Accession number / CP stop and 3'-adjacent 6 nt / CP-frame translation of sequence from immediately after CP stop codon to next in-frame stop codon (*), if present, or to end of available sequence

```
KP256520  UGA CUGAUG LMVTSARAEMPHIRGNSCVRCGFRPPRVVDQRVWGCNFCSYEQYA*
MT893737  UGA CUAGAU LDGQPDDVELPYIDGNACVRCGYAPKYIVDQCVWSCTLCSYEQKA*
MT596820  UGA CUAGAU LDGHLGRTEMPHIRGNSCVRCGYTPPRIVDQYVWKCVVCSYEQHA*
MT596823  UGA CUAGAU LDGHLGRTEMPHIRGNSCVRCGYTPPRIVDQYVWKCVVCSYEQHA*
MT669379  UGA CUAGAU LDGHLGRTEMPHIRGNSCVRCGYTPPRIVDQYVWKCVVCSYEQHA*
FJ403377  UGA CUAGAU LDGHLGRTEMPRIRGNSCARCGYTPPRIVDQYVWKCVVCSYEQHA*
JX073658  UGA CUAGAU LDGHLGRTEMPRIRGNSCARCGYTPPRIVDQYVWKCVVCSYEQHA*
KT445969  UGA CUAGAU LDGHLGRTEMPRIRGNSCARCGYTPPRIVDQYVWKCVVCSYEQHA*
JX463339  UGA CUAGAU LDGHLGRTELPHIRRNSCVRCGFTPPRIVDQYVWSCNMCSYEQHA*
MH184523  UGA CUAGAU LDGHLGRTELPHIRRNSCVRCGFTPPRIVDQYVWSCNMCSYEQHA*
JX463349  UGA CUAGAU LDGHLGRTELPHIRRNSCVRCGFTPPRIVDQYVX
NC_003845 UGA CUAGAU LDGHLGETEMPHIDGNSCVRCGFTPRRIVDQYIWKCDICSYEQYA*
JX463347  UGA CUAGAU LDGHLGGTEMPHIDGNSCVRCGFTPRRIVDQYIWSCDVCSYEQYA*
KR017710  UGA CUAGAU LDGHLGETEMPHIDGNSCVRCGFTPRCIVDQYIWACDICSYEQYA*
MT602530  UGA CUAGAU LDGHLGRTEMPHIDGNTCVRCGFTPPRIVDQYVWKCNICSYEQYA*
MT669385  UGA CUAGAU LDGHLGRTEMPHIDGNTCVRCGFTPPRIVDQYVWKCNICSYEQYA*
MT602533  UGA CUAGAU LDGHLGRTEMPHIDGNTCVRCGFTPPRIVDQYVWKCNICSYEQYA*
MT602536  UGA CUAGAU LDGHLGRTEMPHIDGNTCVRCGFTPPRIVDQYVWKCNICSYEQYA*
MT602539  UGA CUAGAU LDGHLGRTEMPHIDGNTCVRCGFTPPRIVDQYVWKCNICSYEQYA*
```

```
MT602545  UGA CUAGAU LDGHLGRTEMPHIDGNTCVRCGFTPPRIVDQYVWKCNICSYEQYA*
MT602542  UGA CUAGAU LDGHLGRTEMPHIDGNTCVRCGFTPPRIVDQYVWKCNICSYEQYA*
MH299981  UGA CUAGAU LDGPPR*
KY547804  UGA CUAGAU LDGHLGRTELPYIRGNSCVRCGFTPPRIVDQYVWSCNFCSYEQHA*
KY176876  UGA CUAGAU LDGHLGRTELPYIRGNSCVRCGFTPPRIVDQYVWSCNFCSYEQHA*
MF555193  UGA CUAGAU LDGHLGRTESPYIRGNSSFRCGYTPPCS
MH299986  UGA CUAGAU LDGHLGRTELPYIRGNSCVRCGFTPPRIVDQYVWSCSFCSYEQHA*
KX452727  UGA CUAGAU LDGHLGRTELPYIRGNSCVRCGFTPPRIVDQYVWSCNFCSYEQHA*
KX881767  UGA CUAGAU LDGHLGRTELPYIRGNSCVRCGFTPPRIVDQYVWSCNFCSYEQHA*
MF555190  UGA CUAGAU LDGHLGRTESPYIRGNPCLRCGVX
MH299982  UGA CUAGAU LDGHLGRTELPYIRGNSCVRCGFTPPRIVDQYVWSCNFX
JF340383  UGA CUAGAU LDGHLGRTELPYIRCNSCVRCGFTPPRIVDQYVWSCNFCSYEQHA*
JF340384  UGA CUAUAU LYGHLGRTELPYIRGNSCVRSGFTPPRIVDQYVWSCNFCSYEQQA*
EF159704  UGA CUAGAU LDGHLGRTELPYIRGNSCVRCGFTPPRIVDRYVWSCNFCSYEQHA*
FJ561301  UGA CUAGAU LDGHLGRTELPYIRGNSCVRCGFTPPRIVDQYVWSCNFCSYEQHA*
MH299979  UGA CUAGAU LDGHLGRTELPYIRGNSCVRCGFTPPRIVDQYVWSCNFCSYERHA*
JF340381  UGA CUAGAU LDGHLGRTELPYIRGNSCVRCGFTPPRIVDQYVWSCYFCSYEQHA*
KF264469  UGA CUAGAU LDGHLGRTELPYIRGNSCVRCGFTPPRIVDQYVWSCNFCSYEQHA*
KY547795  UGA CUAGAU LDGHLGRTELPYIRGNSCVRCGFTPPRIVDQYVWSCNFCSYEQHA*
KY176873  UGA CUAGAU LDGHLGRTELPYIRGNSCVRCGFTPPRIVDQYVWSCNFCSYEQHA*
FJ655171  UGA CUAGAU LDGHLGRTELPYIRGNSCVRCGFTPPRIVDQYVWSCNFCSYEQHA*
DQ067449  UGA CUAGAU LDGHLGRTELPYIRGNSCVRCGFTPPRIVDQYVWSCNFCSYEQHA*
FJ608537  UGA CUAGAU LDGHLGRTELPYIRGNSCVRYGFTPPRIVDQYVWSCNFCSYEQHA*
JF340382  UGA CUAGAU LDGHLGRTELPYIRGNSCVRCGFTPPRIVEQYVWSYNFCSYEQYT*
MH299985  UGA CUAGAU LDGHLGRTELPYIRGNSCVRCGFTPPRIVDQYVWSCNFCSYERHA*
MH299980  UGA CUAGAU LDGHLGRTELPYIRGNSCVRCGFTPPX
KX523132  UGA CUAGAU LDGHLGRTELPYIRGNSCVRCGFTPPRIVDQYVWSCNFCSYEQHA*
KM504248  UGA CUAGAU LDGHLGRTELPYIRGNSCVRCGFTPPRIVDQYVWSCNFCSYEQHA*
KU509214  UGA CUAGAU LDGHLGRTELPYIRGNSCVRCGFTPPRIVDQYVWSCNFCSYEQHA*
KX394691  UGA CUAGAU LDGHLGRTELPYIRGNSCVRCGFTPPRIVDQYVWSCNFCSYEQHA*
KU242583  UGA CUAGAU LDGHLGRTELPYIRGNSCVRCGFTPPRIVDQYVWSCNFCSYEQHA*
KU496882  UGA CUAGAU LDGHLGRTELPYIRGNSCVRCGFTPPRIVDQYVWSCNFCSYERHA*
KX405022  UGA CUAGAU LDGHLGRTELPYIRGNSCVRCGFTPPRIVDQYVWSCNFCSYERHA*
KX838948  UGA CUAGAU LDGHLGRTELPYIRGNSCVRCGFTPPRIVDQYVWSCNFCSYEQHA*
MG523398  UGA CUAGAU LDGHLGRTELPYIRGNSCVRCGFTPPRIVDQYVWSCNFCSYEQHA*
MG523400  UGA CUAGAU LDGHLGRTELPYIRGNSCVRCGFTPPRIVDQYVWSCNFCSYEQHA*
MH299983  UGA CUAGAU LDGHLGRTELPYI*
MH299984  UGA CUAGAU LDGHLGRTELPYIIGYSCVRCGFTPPRIGNQYVW
MH299978  UGA CUAGAU LDGHLGRTELPYIRGNSCVRCGFTPPRIVD
FJ655173  UGA CUAGAU LDGHLGRTELPYIRGNSCVRCGFTPPRIVDQYVWSCNFCSYEQHA*
MH744487  UGA CUAGAU LDGHLGRTELPYIRGNSCVRCGFTPPRIVDQYVWSCNFCSYEQHA*
JF340380  UAA CUAGAU LDSHLGRTELPYIRGNSCV*
AY501478  UGA CUAGAU LDGHLGRTELPYIRGNSCVRCGFTPPRIVDQYVWSCNFCSYEQHA*
AY501477  UGA CUAGAU LDGHLGRTELPYIRGNSCVRCGFTPPRIVDQYVWSCNFCSYEQHA*
AY501483  UGA CUAGAU LDGHLGRTELPYIRGNSCVRCGFTPPRIVDQYVWSCNFCSYEQHA*
AY501484  UGA CUAGAU LDGHLGRTELPYIRGNSCVRCGFTPPRIVDQYVWSCNFCSYEQHA*
EF159703  UGA CUAGAU LDGHLGRTELPYIRGNSCVRCGFTPPRIVDQYVWSCNFCSYEQHA*
KU242587  UGA CUAGAU LDGHLGRTELPYIRGNSCVRCGFTPPRIVDQYVWSCNFCSYEQHA*
KU496881  UGA CUAGAU LDGHLGRTELPYIRGNSCVRCGFTPPRIVDQYVWSCNFCSYEQHA*
KX394690  UGA CUAGAU LDGHLGRTELPYIRGNSCVRCGFTPPRIVDQYVWSCNFCSYEQHA*
KX838947  UGA CUAGAU LDGHLGRTELPYIRGNSCVRCGFTPPRIVDQYVWSCNFCSYEQHA*
MG523397  UGA CUAGAU LDGHLGRTELPYIRGNSCVRCGFTPPRIVDQYVWSCNFCSYEQHA*
KY547793  UGA CUAGAU LDGHLGRTELPYIRGNSCVRCGFTPPRIVDQYVWSCNFCSYEQHA*
KY547794  UGA CUAGAU LDGHLGRTELPYIRGNSCVRCGFTPPRIVDQYVWSCNFCSYEQHA*
MN631093  UGA CUAGAU LDGHLGRTELPYIRGNSCVRCGFTPPRIVDQYVWSCNFCSYEQHA*
KU378234  UGA CUAGAU LDGHLGRTELPYIRGNSCVRCGFTPPRIVDQYVWSCNFCSYEQHA*
KX523133  UGA CUAGAU LDGHLGRTELPYIRGNSCVRCGFTPPRIVDQYVWVV*
MN631094  UGA CUAGAU LDGHLGRTELPYIRGNSCVRCGFTPPRIVDQYVWSCNFCSYEQHA*
FJ655169  UGA CUAGAU LDGHLGRTELPYIRGNSCVRCGFTPPRIVDQYVWSCNFCSYEQHA*
KF264468  UGA CUAGAU LDGHLGRTELPYIRGNSCVRCGFTPPRIVDQYVWSCNFCSYEQHA*
FJ561304  UGA CUAGAU LDGHLGRTELPYIRGNSCVRCGFTPPRIVDQYVWSCNFCSYEQHA*
FJ655172  UGA CUAGAU LDGHLGRTELPYIRGNSCVRCGFTPPRIVDQYVWSCNLCSYEQHA*
FJ417082  UGA CUAGAU LDGHLGRTELPYIRGNSCVRCGFTPPRIVDQYVWSCNFCSYEQHA*
FJ417083  UGA CUAGAU LDGHLGRTELPYIRGNSCVRCGFTPPRIVDQYVWSCNFCSYEQHA*
AY501476  UGA CUAGAU LDGHLGRTELPYIRGNSCVRCGFTPPRIVDQYVWSCNFCSYEQHA*
AY501480  UGA CUAGAU LDGHLGRTELPYIRGNSCVRCGFTPPRIVDQYVWSCNFCSYEQHA*
AY501482  UGA CUAGAU LDGHLGRTELPYIRGNSCVRCGFTPPRIVDQYVWSCNFCSYEQHA*
EF159702  UGA CUAGAU LDGHLGRTELPYIRGNSCVRCGFTPPRIVDQYVWSCNFCSYEQHA*
FJ655170  UGA CUAGAU LDGHLGRTELPYIRGNSCVRCGFTPPRIVDQYVWSCNFCSYEQHA*
KF264467  UGA CUAGAU LDGHLGRTELPYIRGNSCVRCGFTPPRIVDQYVWSCNFCSYEQHA*
KF264470  UGA CUAGAU LDGHLGRTELPYIRGNSCVRCGFTPPRIVDQYVWSCNFCSYEQHA*
```

```
KU242582  UGA CUAGAU LDGHLGRTELPYIRGNSCVRCGFTPPRIVDQYVWSCNFCSYEQHA*
KU242584  UGA CUAGAU LDGHLGRTELPYIRGNSCVRCGFTPPRIVDQYVWSCNFCSYEQHA*
KU242585  UGA CUAGAU LDGHLGRTELPYIRGNSCVRCGFTPPRIVDQYVWSCNFCSYEQHA*
KU242586  UGA CUAGAU LDGHLGRTKLPYIISYSRVRCEFTPPHKYIEKRQDMVKYNTLVQHYIQYSYHILCKIYSKNVYKIIVX
KX778723  UGA CUAGAU LDGHLGRTELPYIRGNSCVRCGFTPPRIVDQYVWSCNFCSYEQHA*
KY132090  UGA CUAGAU LDGHLGRTELPYIRGNSCVRCGFTPPRIVDQYVWSCNFCSYEQHA*
KY176870  UGA CUAGAU LDGHLGRTELPYIRGNSCVRCGFTPPRIVDQYVWSCNFCSYEQHA*
KY547796  UGA CUAGAU LDGHLGRTELPYIRGNSCVRCGFTPPRIVDQYVWSCNFCSYEQHA*
KY547805  UGA CUAGAU LDGHLGRTELPYIRGNSCVRCGFTPPRIVDQYVWSCNFCSYEQHA*
MG523399  UGA CUAGAU LDGHLGRTELPYIRGNSCVRCGFTPPRIVDQYVWSCNFCSYEQHA*
MH299987  UGA CUAGAU LDGHLGRTELPYIRGNSCVRCGFTPPPIRLVV*
MT682308  UGA CUAGAU LDGHLGRTELPYIRGNSCVRCGFTPPRIVDQYVWSCNFCSYEQHA*
KY547799  UGA CUAGAU LDGHLGRTELPYIRGNSCVRCGFTPPRX
KJ825822  UGA CUAGAU LDGHLGRTELPYIRGNSCVRCGFTPPRIVDQYVWSCNFCSYEQHA*
GQ370526  UGA CUAUCU LSSHLGNTEMPYINGNSCVRCGFTPRRIVDQYVWDCTFCSYEQRA*
MK732934  UGA CUAUUA LLSHPDDVGLPHIHGNSCVRCGFAPRHVVDQRVWDCTFCSYEQYA*
AY354406  UGA CUAUCU LSSHLGDTELPHINGNTCVRCGFTPRRVVDQHVWDCTFCSYEQYA*
JX463336  UGA CUAUCU LSSHLGDTELPHINGNTCVRCGFTPRRVVDQHVWDCTFCSYEQYA*
MT360269  UGA CUAUCU LSSHLGDTELPHINGNTCVRCGFTPRRVVDQHVWDCTFCSYEQYA*
```

CLUSTER 2

Alignment of 3'UTRs from the 3 segments of a representative genome

```
RNA2            UAGAACGAGUUGAACGCGUUCGAAUUUCCUCGAAUAGAGAAGAAUCGUACCACCG--UGU
RNA3            UAGAGUGAGAUAAGCACACUCGAAUUUCUCCGA--AUGGAAAGUUCGCACCACCGAUAGU
RNA1            UAGAGUGAGAUGAACGCACUCGA--UUCCCAGAUUAUGGAAGAACCGUACCACCG--UGU
                ****  *** * * * *  ****  ***   **  *  ***    ** *******   **

RNA2            GGGU-UUGC-GAUUAGUUCCUAGAGAGUUGCUUCCGGCUCUCGAGUUCAUAGGUACUUAC
RNA3            GGAUAUUGCGAAAUAGAUUUCUGAAAGUCGCUUCCCGGCUUUCAUGCUUGGAAAUCUUAC
RNA1            GGAU-UUAUCGGUUAUCUCUUUUGAAGUUGUUUCCUUACUUCAGUGUUCUGAGAGAUUAC
                ** * **      **  *       *** * ****     *               ****

RNA2            CUGCUUU-GCAGAUGCCUACGACGUGAAGUCGUAGAUGCCCCGAUAGGGAAGC
RNA3            CUGCGUUAGCAGAUGCCCACAACGUGAAGUUGUGGAUGCCCCGUUAGGGAAGC
RNA1            CUGCUUACGCAGAUGCCUACAACGAGAAGUUGUAGAUGCCCCGUUAGGGAAGC
                **** *  ********* **  *** ***** ** ********* *********
```

```
RNA1 NC_004362 168 nt
RNA2 NC_004363 168 nt
RNA3 NC_004364 171 nt
```

Note: No insert in RNA3, so no evidence for RT domain.

All sequences in the cluster

```
    101 Prunus necrotic ringspot virus
      1 Apple mosaic virus (U03857*)
```

* tblastn analysis indicates this is a mislabelled prunus necrotic ringspot virus sequence: 100% coverage x 96% a.a. identity to closest prunus necrotic ringspot virus sequence, but 100% coverage x 51% a.a. identity to closest apple mosaic virus sequence

Accession number / CP stop and 3'-adjacent 6 nt / CP-frame translation of sequence from immediately after CP stop codon to next in-frame stop codon (*), if present, or to end of available sequence

```
MZ451050  UAG GGCGAG GEISTPEFPP*
MZ451054  UAG GGCGAG GEISAPEFPP*
MZ451055  UAG GGCGAG GEISAPEFPP*
L38823    UAG AGUGAG SEISTLEFLR*
AJ133207  UAG AGUGAG SEISTLEFLR*
AJ133210  UAG AGUGAG SEISTLEFLR*
FJ546090  UAG AGUGAG SEISTLEILR*
FJ546091  UAG AGUGAG SEISTLEILR*
AJ133203  UAG AGUGAG SEISTLEFSPNGKFAPPIVDTAN*
U03857    UAG AGUGAG SEISTLEFLRMESSHHR*
AJ133201  UAG AGUGAG SEISTLEFLRMESSHHR*
JQ005047  UAG AGUGAG SEISTLEFSPNGKFAPPIVDTAN*
AJ133209  UAG AGUGAG SEISTLEFSPNGKFAPPLVDIAN*
AJ133202  UAG AGUGAG SEISTLEFSPNGKFAPPIVDIAN*
KY883322  UAG AGUGAG SEISTLEFSPNGKFAPPIVDIAN*
AJ133204  UAG AGUGAG SEISTLEFSPNGKFAPPIVDIAN*
AJ133199  UAG AGUGAG SEISTLEFSPNGKFAPPIVDIAN*
MF069046  UAG AGUGAG SEISTLEFSPNGKFAPPIVDIAN*
KY883335  UAG AGUGAG SEISTLEIFSEWKVRTTDSGYCELDF*
S78312    UAG AGUGAG SEISTLEFSPNGKFAPPIVDIAN*
AJ133212  UAG AGUGAG SEISTLEFSPNGKFAPPIVDIAN*
MZ451049  UAG AGUGAG SEISTLDFSPNGKFAPPIVDIAN*
MZ451048  UAG AGUGAG SEISTLDFSPNGKFAPPIVDIAN*
AF013286  UAG AGUGAG SEISTLEFLQMESSHTRISP
AJ133208  UAG AGUGAG SEISTLEFSPNGKFAPPIVDIAN*
JQ005040  UAG AGUGAG SEISTLEFSPNGKFAPPIVDIAN*
JQ005031  UAG AGUGAG SEISTLEFSPNGKFAPPIVDIAN*
AJ133200  UAG AGUGAG SEISTLEFSPNGKFAPPIVDIAN*
JQ005039  UAG AGUGAG SEISTLEFSPNGKFAPPVVDIAN*
```

JQ005046 UAG AGUGAG SEISTLEFSPNGKFAPPIVDIAN*
JQ005050 UAG AGUGAG SEISTLEFSPNGKFAPPIVDIAN*
JQ005052 UAG AGUGAG SEISTLEFSPNGKFAPPIVDIAN*
JQ005053 UAG AGUGAG SEISTLEFSPNGKFAPPIVDIAN*
JQ005054 UAG AGUGAG SEISTLEFSPNGKFAPPIVDIAN*
JQ005055 UAG AGUGAG SEISTLEFSPNGKFAPPIVDIAN*
JQ005056 UAG AGUGAG SEISTLEFSPNGKFAPPIVDIAN*
JQ005057 UAG AGUGAG SEISTLEFSPNGKFAPPIVDIAN*
JQ005058 UAG AGUGAG SEISTLEFSPNGKFAPPIVDIAN*
MZ451047 UAG AGUGAG SEISTLEFSPNGKFAPPIVDIAN*
MZ451060 UAG AGUGAG SEISTLEFSPNGKFAPPIVDIAN*
MZ451061 UAG AGUGAG SEISTLEFSPNGKFAPPIVDIAN*
MZ451062 UAG AGUGAG SEISTLEFSPNGKFAPPIVDIAN*
MZ451063 UAG AGUGAG SEISTLEFSPNGKFAPPIVDIAN*
AJ133205 UAG AGUGAG SEISTLEFSPNGKFAPPIVDIAN*
JN416773 UAG AGUGAG SEISTLDFSPNGKFAPPIVDIAN*
JQ005030 UAG AGUGAG SEISTLEFSPNGKFAPPIVDIAN*
JQ005032 UAG AGUGAG SEISTLEFSPNGKFAPPIVDIAN*
JQ005033 UAG AGUGAG SEISTLEFSPNGKFAPPIVDIAN*
JQ005034 UAG AGUGAG SEISTLEFSPNGKFAPPIVDIAN*
JQ005035 UAG AGUGAG SEISTLEFSPNGKFAPPIVDIAN*
JQ005036 UAG AGUGAG SEISTLEFSPNGKFAPPIVDIAN*
JQ005037 UAG AGUGAG SEISTLEFSPNGKFAPPIVDIAN*
JQ005038 UAG AGUGAG SEISTLEFSPNGKFAPPIVDIAN*
JQ005041 UAG AGUGAG SEISTLEFSPNGKFAPPIVDIAN*
JQ005042 UAG AGUGAG SEISTLEFSPNGKFAPPIVDIAN*
JQ005043 UAG AGUGAG SEISTLEFPPNGKFAPPIVDIAN*
JQ005044 UAG AGUGAG SEISTLEFSPNGKFAPPIVDIAN*
JQ005045 UAG AGUGAG SEISTLEFSPNGKFAPPIVDIAN*
KU977374 UAG AGUGAG SEISTLEFSPNGKFAPPIVDIAN*
KU977375 UAG AGUGAG SEISTLEFSPNGKFAPPIVDIAN*
KU977376 UAG AGUGAG SEISTLEFSPNGKFAPPIVDIAN*
KU977377 UAG AGUGAG SEISTLEFSPNGKFAPPIVDIAN*
KU977378 UAG AGUGAG SEISTLEFSPNGKFAPPIVDIAN*
KY883325 UAG AGUGAG SEISTLEFSPNGKFAPPIVDIAN*
MF198443 UAG AGUGAG SEISTLEFSPNGKFAPPIVDIAN*
MH730938 UAG AGUGAG SEISTLEFSPNGKFAPPIVDIAN*
MH730939 UAG AGUGAG SEISTLEFSPNGKFAPPIVDIAN*
ON304061 UAG AGUGAG SEISTLEFSPNGKFAPPIVDIAN*
MZ451052 UAG AGUGAG SEISTLEVSPNGKFAPPIVDIAN*
MZ451056 UAG AGUGAG SEISTLEFLRMESSHHR*
AJ133213 UAG AGUGAG SEISTLEFLRMESSHHR*
KT444703 UAG AGUGAG SEISTLEFLRMESSHHR*
Y07568 UAG AGUGAG SEISTLEFLRMESSHHR*
MF069040 UAG AGUGAG SEISTLEFLRMESSHHR*
MZ451051 UAG AGUGAG SEISTLEFLRMESSHHR*
MZ451057 UAG AGUGAG SEISTLEFLRMESSHHR*
AJ133206 UAG AGUGAG SEISTLEFLRMESSYHR*
LC382468 UAG AGUGAG SEISTLEFLRMESSYHR*
MZ451059 UAG AGUGAG SEISTLEVSPNGKFAPPIVDIAN*
AJ133211 UAG AGUGAG SEISTLEFLRMESSHHR*
AM408910 UAG CCCGGG PGGNH*
AF013287 UAG AGUGAG SEISTLEFLRMESSHTRISP
MZ451053 UAG AGUGAG SEISTLEFSPNGKFAPPLVDIAN*
KX650619 UAG AGUGAG SEISTLEFLRMESSHHR*
MH730940 UAG AGUGAG SEISTLEFLRMESSHHR*
MH730941 UAG AGUGAG SEISTLEFLRMESSHHR*
MH730942 UAG AGUGAG SEISTLEFLRMESSHHR*
FJ546092 UAG AGUGAG SEISTLEFLRMESSHHR*
MN656197 UAG AGUGAG SEISTLEFLRMESSHHR*
JQ005048 UAG AGUGAG SEISTLEFLRMESSHHR*
JQ005051 UAG AGUGAG SEISTLEFLRMESSHHR*
JQ005049 UAG AGUGAG SEISTLEFLRMESSHHR*
MZ451065 UAG AGUGAG SEISTLEFLRMESSHHR*
AJ969095 UAG AGUGAG SEISTLEFLRMESSHTRISP
MZ451058 UAG AGUGAG SEISTLEFLRMESSHHR*
MZ451064 UAG AGUGAG SEISTLEFLRMESSHHR*
NC_004364 UAG AGUGAG SEISTLEFLRMESSHHR*
FJ610344 UAG AGUGAG SEISTLEFFSEWKVRTTDSGYCELDF*
JN416776 UAG AGUGAG SEISTLECLRMESSYHR*
MN656194 UAG AGUGAG SEISTLEFLRMESSHHR*

```
AY948440  UAG AGUGAG SEISTLEFLRMESSHHR*
AY948441  UAG AGUGAG SEISTLEFLRMESSHHR*
```

CLUSTER 3

Alignment of 3'UTRs from the 3 segments of a representative genome

```
RNA1            UG----------------------------------------------------------
RNA2            UGACCAUACUAUCUAUUUGU----------------------------------------
RNA3            UGACUAUAUGAUCCAUCAUUUGAUUGUGCUUCCACUAUGAGUAUUCCUAGGAAUAUUCGU
                **

RNA1            -------------------------------ACCGUUCAGAUUUUGUCACUGGACGUAAA
RNA2            -----------------------UAGAAUCCACCAUUCAGAUUUUGUCACUGAAUGU--C
RNA3            AGUUGGAAAUGCUGCUUUUGCAACAGAAUCCACCAUUCAGAGUUUGUCACUGAAUGU-UA
                                               *** ****** ********** * **

RNA1            AAUCCUUUUGGUUAACUCGUACUGCGUACUUUUUGAGUUAAGAUAAAAAUGCCCAUUGUA
RNA2            AAUCCUUUUGGUUAACCUGCACUAAGUGCGUAAAAGGUUAAGAUAAAAAUGCCCAUUGUA
RNA3            AAUCCUUUUGGUUAACCUGCACUAAGUGCGUAAAAGGUUAAGAUGAAAAUGCCCAUUGUA
                ****************  * ***  ** * *     ******** ***************

RNA1            UCCUGAAUGGAUGACACUUUUUAUUGCCUACAAA-UUUGUAGAUGCCCUCACCGUAAGGU
RNA2            UCCUGAAUGGAUGACACUUUUUAUUGCCUACAGA-UUUGUAGAUGCCCUCACCGUAAGGU
RNA3            UCCUGAAUGGAUGACACUUUUCAUUGCCUACAAAUUUUGUACAUGCCCUCACCGUAAGGU
                ********************* ********** * ****** ******************

RNA1            GAGGAUGCCCCUU--AAGGAUGC
RNA2            GAGGAUGCCCCUUUAAGGGAUGC
RNA3            GAGGAUGCCCCUUUAAGGGAUGC
                *************  * ******
```

RNA1 NC_008039 171 nt
RNA2 NC_008037 198 nt
RNA3 NC_008038 262 nt

All sequences in the cluster

39 Prune dwarf virus

Accession number / CP stop and 3'-adjacent 6 nt / CP-frame translation of sequence from immediately after CP stop codon to next in-frame stop codon (*), if present, or to end of available sequence

```
KU215404  UGA CUACAU LHDPSFDCTSTMSIPKNIRSWKCCFCNRIHHSDFVTEC*
MF078480  UGA CUAUAU LYDPSFDCTSTMSIPRNIRSWKCCFCNRIHHSDFVTEC*
MZ221000  UGA CUAUAU LYDPLFDCTSTMSIPRNIRSWRCCFCNRIHHSDFVTEC*
MK560342  UGA CUAUAU LYDPLFDCTSTMSIPRNIRSWRCCFCNRIHHSDFVTEC*
MZ221002  UGA CUAUAU LYDPLFDCTSTMSIPRNIRSWRCCFCNRIHHSDFVTEC*
MZ221003  UGA CUAUAU LYDPLFDCTSTMSIPRNIRSWRCCFCNRIHHSDFVTEC*
MZ220997  UGA CUAUAU LYDPLFDCTSTMSIPRNIRSWRCCFCNRIHHSDFVTEC*
KY883330  UGA CUAUAU LYDPSFDCASTMSIPRNIRSWKCCFCNGIHHSDFVTEC*
MK834276  UGA CUAUAU LYDPLFDCTSTMSIPRNIRSWRCCFCNRIHHSDFVTEC*
MZ221009  UGA CUAUAU LYDPSFDCASTMSIPRNIRSWKCCFCNGIHHSDFVTE*
MZ220992  UGA CUAUAU LYDPSFDCTSTMSIPRNIHSWKCCFCNGIHHSDFVTEC*
KY883331  UGA CUAUAU LYDLSFDCASTMSIPRNIRSWKCCFCNRIHHSEFVTEC*
MK392156  UGA CUAUAU LYDPLFDCTSTMSVPRNIRSWKCCFLN
MZ221005  UGA CUAUAU LYDPSFDCASTMSIPRNIRSWKCCFCNGIHHSDFVTEC*
MZ221010  UGA CUAUAU LYDPSFDCASIMNIPRNIRSWKCCFCNGIHHSDFVTEC*
MZ220998  UGA CUAUAU LYDSSFDCASTMSIPRNIHSWKCCFCNGIHHSDFVTE*
KU215407  UGA CUAUAU LYDPSFDCASTMSIPRNIRSWKCCFCNRIHHSEFVTE*
MK369931  UGA CUAUAU LYDPSFDCASTMSIPRNIRSWKCCFCNRIHHSEFVTE*
MZ220994  UGA CUAUAU LYDSSFDCASTMSIPRNIRSWKCCFCNGIHHSDFVTE*
MZ221012  UGA CUAUAU LYDPSFDCASTMSIPRNIRSWKCCFCNRIHHSEFVTEC*
MT013235  UGA CUAUAU LYDLSFDCASTMSIPRNIRSWKCCFSNRIHHSEFVTEC*
MZ221014  UGA CUAUAU LYDPSFDCASTMSIPKNIRSWKCCFCNRIHHSEFVTEC*
MZ221008  UGA CUAUAU LYDPSFDCASTMSIPKNIRSWKCCFCNRIHHSEFVTEC*
MZ221006  UGA CUAUAU LYDPSFDCASTMSIPKNIRSWKCCFCNRIHHSEFVTEC*
MZ221007  UGA CUAUAU LYDPSFDCASTMSIPKNIRSWKCCFCNRIHHSEFVTEC*
MW334945  UGA CUAUAU LYDPSFDCASTMSIPKNIRSWKCCFCNRIHHSEFVTEC*
```

```
MZ220993  UGA CUAUAU LYDPSFDCASTMSIPKNIRSWKCCFCNRIHHSEFVTEC*
MZ220999  UGA CUAUAU LYDPSFDCTSTMSIPRNIRSWKCCFCNRIHHSEFVTEC*
MZ221001  UGA CUAUAU LYDPSFDCTSTMSIPRNIRSWKCCFCNRIHHSEFVTEC*
MZ221013  UGA CUAUAU LYDPSFDCTSTMSIPRNIRSWKCCFCNRIHHSEFVTEC*
MZ291946  UGA CUAUAU LYDPSFDCTSTMSIPRNIRSWKCCFCNRIHHSEFVTEC*
MZ291949  UGA CUAUAU LYDPSFDCTSTMSIPRNIRSWKCCFCNRIHHSEFVTEC*
NC_008038 UGA CUAUAU LYDPSFDCASTMSIPRNIRSWKCCFCNRIHHSEFVTEC*
MZ291943  UGA CUAUAU LYDPSFVCASTMSIPRNIRSWKCCFCNRIHHSEFVTEC*
MZ221004  UGA CUAUAU LYDPSFDCASTMSIPRNIRSWKCCFCNRIHHSEFVTEC*
MK392155  UGA CUAUAU LYDPSFDCASTMSIPKNIRSWKCCFLDX
MZ220996  UGA CUAUAU LYDPSFDCASTMSIPRNIRSWKCCFCNRIHHSEFVTEC*
MZ220995  UGA CUAUAU LYDPSFDCTSTMSIPKNIRSWKCCFCNGIHHSDFVTEC*
MZ221011  UGA CUAUAU LYDPSFDCASTMSIPKNIRSWKCCFCNGIHHSDFVTEC*
```

CLUSTER 4

Alignment of 3'UTRs from the 3 segments of a representative genome

```
RNA3            UGACUGGUUAAUUACCUCGAAGAAAACUUCGAGGUGCCUCACAUUUUACCGACUAUUGUU
RNA1            UGAUUG--UCAUUAUUU---------------------------------GACAUUUGUU
RNA2            ------------------------------------------------------------


RNA3            GGUUCAUGUGUGAGAUGCGGAUUCCGCCCACGGAGAGUGCCAGACGCACGCUAUUUGUGG
RNA1            UAUUCCCAGUUUACUGGAUGCCGUCGUUGACGAUGAUUUAUAUAUAC----------UAG
RNA2            ---------------------------------------------------------UAA
                                                                         *

RNA3            UGUGGCAUGUGCAAAUAUUUGCACUAUGCAUAGUUACCUCGCAUCCGGGUAUAAAUCUAU
RNA1            GUUGGCAUU--------------UUGAGCGCAUCUUACUCAAAUCCUAGU----------
RNA2            CAUGGCAG---------------CUGAGCGCAUCUAUCUCAGCGGUUAAU----------
                  *****                 *  **  *  *  ***         *

RNA3            GAAUUUCGCUAGAACAUUCCGAAUCGGACGAUUUCCACCUGAAAAGGUGAGACCUUUGGG
RNA1            ------------------------------AUUUCCA-----------------------
RNA2            -----------------------------GGUUGCUA-----------------------
                                               ** * *

RNA3            UCGAUGUUCUAAUGGUUCCGUUAAUAAUAUUGAUUAUUAACGAUGCCUCC-AAUUAAAGA
RNA1            UUAAUA-UCUAAUGA-------------------UAUUAAUGAUGCCUCUUAAUAUAAGA
RNA2            UUAAUA-UCUAAUGA-------------------UAUUAAUAAUGCCUCCAAAUU--GGA
                *  **  *******                    ******  *******  ***    **

RNA3            GAUGC
RNA1            GAUGC
RNA2            GAUGC
                *****


RNA1 NC_011553 163 nt
RNA2 NC_011554  89 nt
RNA3 NC_011555 304 nt
```

All sequences in the cluster

```
     38 Blackberry chlorotic ringspot virus
```

Accession number / CP stop and 3'-adjacent 6 nt / CP-frame translation of sequence from immediately after CP stop codon to next in-frame stop codon (*), if present, or to end of available sequence

```
NC_011555 UGA CUGGUU LVNYLEENFEVPHILPTIVGSCVRCGFRPRRVPDARYLWCGMCKYLHYA*
JX429894  UGA CUGGUG LVNYLEGDFEMPHDLPEIAGSCVRCGFRPRRVPNALYWWCDMCNYLHYA*
JX429865  UGA CUGGUG LVNYLEGDFEMPHDLPEIAGSCVRCGFRPRRVPNALYWWCDMCNYLHYA*
JX429891  UGA CAGGUG QVKYLEGDFEMPHDLPEIAGSCVRCGFRPRRVPDALYWWCDMCNYLHYA*
JX429881  UGA CAGGUG QVKYLEDNFEMPHDLPEIAGSCVRCGFRPRRVPDALYWWCDMCNYLHYA*
JX429883  UGA CAGGUG QVKYLEDNFEMPHDLPEIAGSCVRCGFRPRRVPDALYWWCDMCNYLHYA*
JX429888  UGA CUGGUG LVNHLEGNFEMPHDLPEVAGSCVRCGFRPRRVPDALYWWCDMCNYLHYA*
OK338426  UGA CUGGUG LVNCLEGDFEMPHNLPEIAGSCVRCGFRPRRVPDALYWWCDMCNYLHYA*
KX834012  UGA CUGGUG LVNYLEGDFEMPHNLPEIAGSCVRCGFRPRRVPDALYWWCDMCNYLHYA*
ON557618  UGA CUGGUG LVNYLEGDFEMPHNLPEIAGSCVRCGFRPRRVPDALYWWCDMCNYLHYA*
LC592046  UGA CUGGUG LVNYLEGDFEMPHNLPEIAGSCVRCGFRPRRVPDALYWWCDMCNYLHYX
JX429889  UGA CUGGUG LVNHLEGNFEMPHDLPEVAGSCVRCGFRPRRVPDALYWWCDMCNYLHYA*
JX429864  UGA CUGGUG LVNHLEGNFEMPHDLPEVAGSCVRCGFRPRRVPDALYWWCDMCNYLHYA*
JX429869  UGA CUGGUG LVNHLEGNFEMPHDLPEVAGSCVRCGFRPRRVPDALYWWCDMCNYLHYA*
JX429885  UGA CUGGUG LVNYLEGDFEMPHDLPEVAGSCVRCGFRPRRVPDALYWWCDMCNYLHYA*
JX429874  UGA CUGGUG LVNRLEGNFEMPHDLPEVAGSCVRCGFRPRRVPDALYWWCDMCNYLHYA*
JX429872  UGA CUGGUG LVNHLESNFEMPHDLPEVAGSCVRCGFRPRRVPDALYWWCDMCNYLHYA*
JX429871  UGA CUGGUG LVNHLEGNFEMPHDLPEVAGSCVRCGFRPRRVPDALYWWCDMCNYLHYA*
JX429879  UGA CUGGUG LVNHLEGNFEMPHDLPEVAGSCVRCGFRPRRVPDALYWWCDMCNYLHYA*
JX429877  UGA CUGGUG LVNHLEGNFEMPHDLPEAAGSCVRCGFRPRRVPDALYWWCDMCNYLHYA*
JX429882  UGA CUGGUG LVNHLEGNFEMPHDLPEVAGSCVRCGFRPRRVPDALYWWCDMCNYLHYA*
```

```
JX429884  UGA CUGGUG LVNHLEGNFEMPHDLPEVAGSCVRCGFRPRRVPDALYWWCDMCNYLHYA*
JX429895  UGA CUGGUG LVNHLEGNFEMPHDLPEVAGSCVRCGFRPRRVPDALYWRCDMCNYLHYA*
JX429873  UGA CUGGUG LVNHLEGNFEMPHDLPEVAGSCVRCGFRPRRVPDALYWWCDMCNYLHYA*
JX429868  UGA CUGGUG LVNHLEGNFEMPHDLPEVAGSCVRCGFRPRRVPDALYWWCDMCNYLHYA*
JX429870  UGA CUGGUG LVNHLEGNFEMPHDLPEVAGSCVRCGFRPRRVPDALYWWCDMCNYLHYA*
JX429878  UGA CUGGUG LVNHLEGNFEMPHDLPEVAGSCVRCGFRPRRVPDALYWGCDMCNYLHYA*
JX429887  UGA CUGGUG LVNHLEGNFEMPHDLPEVAGSCVRCGFRPRRVPDALYWWCDMCNYLHYA*
GQ325716  UGA CUGGUG LVNHLEGNFEMPHDLPEVAGSCVRCGFRPRRVPX
JX429866  UGA CUGGUG LVNHLEGNFEMPHDLPEVAGSCVRCGFRPRRVPDALYWGCDMCNYLHYA*
JX429867  UGA CUGGUG LVNHLEGNFEMPHDLPEVAGSCVRCGFRPRRVPDALYWWCDMCNYLHYA*
JX429875  UGA CUGGUG LVNHLEGNFEMPHDLPEVAGSCVRCGFRPRRVPDALYWWCDMCNYLHYA*
JX429876  UGA CUGGUG LVNHLEGNFEMPHDLPEVAGSCVRCGFRPRRVPDALYWWCDMCNYLHYA*
JX429890  UGA CUGGUG LVNHLEGNFEMPHDLPEVAGSCVRCGFRPRRVPDALYWWCDMCNYLHYA*
JX429893  UGA CUGGUG LVNHLEGNFEMPHDLPEVAGSCVRCGFRPRRVPDALYWWCDMCNYLHYA*
JX429892  UGA CUGGUG LVKHLEGDFEMPHDLPEIAGSCVRCGFRPRRVPDALYWWCDMCNYLHYA*
JX429886  UGA CUGGUG LVNYLEGDFEMPHDLPEIAGSCVRCGFRPRRVPDALYWWCDMCNYLHYA*
JX429880  UGA CUGGUG LVNYLEGDFEMPHDLPEIAGSCVRCGFRPRRVPDALYWWCDMCNYLHYA*
```

CLUSTER 5a

Alignment of 3'UTRs from the 3 segments of a representative genome

```
RNA1            UAG---------------------------------------------------------
RNA2            UAA---------------------------------------------------------
RNA3            UAGCAAUUAGGUCGGGAUAUUUCCCGACUUCGGGAUGUAUCCCGACGUGCUAAAGUUCCU
                **

RNA1            ------------------------------------------------------------
RNA2            ------------------------------------------------------------
RNA3            AACGAUUUUAUUGUUGGAUGCGAACCGGCUUACGACAUUGAUAUGUGUGAGCAGGCUCAU


RNA1            ------------------------------------------------------------
RNA2            ------------------------------------------------------------
RNA3            GAUGGUUAUCCUGAGGUUAAUCCUGCGGUCUUGAGACCGAGGUUUUUCUCAGUGCCGAGG


RNA1            ------------------------------------------------------------
RNA2            ------------------------------------------------------------
RNA3            ACGGUUAGACGUUCUCGAAGGCUAAAUCCACGUUUAGUGGAUGAAGCGAAUUUACCAUAC


RNA1            ------------------------------------------------------------
RNA2            ------------------------------------------------------------
RNA3            UUCACAGGUGAGUGCGUGAGGUGUGGUUAUACUCCUAAGUCUCUCAUUGAUCAGAGAGAA


RNA1            ---------------------------------------------GUAAA------AUCU
RNA2            ---------------UUUCGUUUGUCGUUUAUA------------CUAAAACA---AUCU
RNA3            UGGGAAUGUUCAAGCUGUUAUAUGUUGUAUGCAGCUUGAAGAUUUCUAAAAUGAUUAUCU
                                                              ****      ****

RNA1            U-------UAUGAGUUGUACCGGACUAGGUCCGAAGUUGUACAAGUAGUCUCCUCUCAUC
RNA2            UGUUUUAAUGCAAGUUAAGUUGGACAAGGUCCAGUGUAACUUAGGUAAGUUUCUCCUAUC
RNA3            UGUUUUAAUGCAAGUUAAGUUGGACAAGGUCCAGUGUAACUUAAGUAAGUUUCUCCUAUC
                *       *   ****     **** ******   **     * ***   * ***  ***

RNA1            GCCAUCUGGGUGGGGUUUUAGGAGUCUAUGCCAAACUCUUUGAGUUUGAUGCCAAUUCAG
RNA2            GCCAUCUGGAUGGGAUUUAAGAGACUUAUGUCAAACUCUUUGAGUUUGAUGCCAAUUCAG
RNA3            GCCAUCUGGAUGGGAUUUAAGAGACUUAUGCCAAACUCUUUGAGUUUGAUGCCAAUUCAG
                ********* **** *** **     **** *****************************

RNA1            UUUUCGUCUGAAUUGAUGCCCGAAAGGAUGGC
RNA2            UUUUCGUCUGAAUUGAUGCCCGAAAGGAUGGC
RNA3            UUUUCGUCUGAAUUGAUGCCCGAAAGGAUGGC
                ********************************
```

RNA1 NC_006566 157 nt
RNA2 NC_006567 185 nt
RNA3 NC_006568 512 nt

All sequences in the cluster

1 Fragaria chiloensis latent virus

Accession number / CP stop and 3'-adjacent 6 nt / CP-frame translation of sequence from immediately after CP stop codon to next in-frame stop codon (*), if present, or to end of available sequence

NC_006568 UAG CAAUUA
QLGRDISRLRDVSRRAKVPNDFIVGCEPAYDIDMCEQAHDGYPEVNPAVLRPRFFSVPRTVRRSRRLNPRLVDEANLPYFTGECVRCGY
TPKSLIDQREWECSSCYMLYAA*

CLUSTER 5b

Alignment of 3'UTRs from the 3 segments of a representative genome

```
RNA1            ------UGAGACCGCGUUUA----------------------------------------
RNA2            ------------------------------------------------------------
RNA3            UAGCAGUUAGGUCGGGUUAAUACCCAGUUUCGGGUUUUAGCCCGACGCGUUGAGAUUCCU


RNA1            ------------------------------------------------------------
RNA2            ------------------------------------------------------------
RNA3            GGGAUUGAUCCCAGAUGCGAGUUUGAUGCUCAAGGAUCGUUGACCUUUCGGUCAAGAUCA


RNA1            ------------------------------------------------------------
RNA2            ------------------------------------------------------------
RNA3            GUUCCGAGGGUUUUUAAUAGACCUCGAAGACUCAAUCCUAGAUUAUCUAAGGAGGAAGAA


RNA1            ------------------------------------------------------------
RNA2            ------------------------------------------------------------
RNA3            GAAGCCCUUGAUCAUUUCGAUCAAGUGGACAAUUCUGACGGUGCAGAUUAUCUGGAUGAU


RNA1            -------------------------GUGGUCGUGUGGUUUUAUUACCGAAAAUCUUGUUU
RNA2            ------------------UAGACGUGCGGUCGCGUGGUUUUAUUACCGAAAAUCUUGUUU
RNA3            UGGCGAUCGUACGUUGAUUAGACGUGCGGUCGCGUGGUUUUAUUACCGAAAAUCUUGUUU
                                         * ***** ***************************

RNA1            CGAUGCAAGUUAAGUUGGACAAGGUCCGCUUAUAACUUAAGUAAGUUUCACCUAUCGCCA
RNA2            CGAUGCAAGUUAAGUUGGACAAGGUCCGCUUAUAACUUAAGUAAGUUUCACCUAUCGCCA
RNA3            CGAUGCAAGUUAAGUUGGACAAGGUCCGCUUAUAACUUAAGUAAGUUUCACCUAUCGCCA
                ************************************************************

RNA1            UCUGGAUGGGAUAUUGGAACUUAUGCCAAGCUCUUUAGAGUUUGAUGCUAAUUCAGUUUU
RNA2            UCUGGAUGGGAUAUUGGAACUUAUGCCAAGCUCUUUAGAGUUUGAUGCUAAUUCAGUUUU
RNA3            UCUGGAUGGGAUAUUGGAACUUAUGCCAAGCUCUUUAGAGUUUGAUGCUAAUUCAGUUUU
                ************************************************************

RNA1            CGUCUGAAUUAAUGCCCGAAAGGAUGGC
RNA2            CGUCUGAAUUAAUGCCCGAAAGGAUGGC
RNA3            CGUCUGAAUUAAUGCCCGAAAGGAUGGC
                ****************************


RNA1 MN527502 197 nt
RNA2 MN527503 190 nt
RNA3 MN527504 448 nt
```

All sequences in the cluster

    32 Potato yellowing virus

Accession number / CP stop and 3'-adjacent 6 nt / CP-frame translation of sequence from immediately after CP stop codon to next in-frame stop codon (*), if present, or to end of available sequence

```
MN527501  UAG CAGUUA
QLGRVEAQSRVLTRRVEIPRNISRCGFDTQGSLTLRSRSIPRVVRPRRLNPRLSKKEEEALDYFDQVDNSDGADYLSDWRSYND*
MN527514  UAG CAAUCA
QSSRVSSRFRVLARRVEVPGIIPRCEFDAQGSLTSRSRSVPRVVKPRRLNPRLSKKEEEALDHFDQVDDSDGADYLNDWRSYVY*
MN527498  UAG CAGUUA
QLGRVNTQFRVLARRVEIPGIDPRCEFDAQGSLTLRSRSVPRVFRPRRLNPRLSKEEEEALDHFDQVDNSDGADYLDDWRSYVD*
MN527504  UAG CAGUUA
QLGRVNTQFRVLARRVEIPGIDPRCEFDAQGSLTFRSRSVPRVFNRPRRLNPRLSKEEEEALDHFDQVDNSDGADYLDDWRSYVD*
MN418002  UAG CAGUUA
QLGRVNTQFRVLARRVEIPGIDPRCEFDAQGSLTLRSRSXXXXXRPRRLNPRLSKEEEEALDHFDQVDNSDGADYLDDWRSYVD*
```

MN548139 UAG CAAUUA
QLGRVNTQFRVLARRVEIPGIDPRCEFDAQGSLTLRSRSVPRVFNRPRRLNPRLSKKEEEALDHFDQVDNSDGADYLDDWRSYVD*
MH937420 UAG CAAUUA
QLGRVNTQSRVLARRVEIPGIDPRCEFDAQGSLTLRSRSVPRVIRPRRLNPRLSKEEEEALDHFDQVDNSDGADYLDDWRSYVD*
MN544551 UAG CAAUUA
QLGRVNTQSRVLARRVEIPGIDPRCEFDAQGSLTLRSRSVPRVFRPRRLNPRLSKEEEEALDHFDQVDNSDGADYLDDWRSYVD*
MN527519 UAG CAAUCA
QSSRVDSRFRVLARRVEVPGIIPRCEFDAQGSSTLRSRSVPRVVKPRRLNPRLSKKEEEALDYFDQVNNSDGADYLNDWRSYVY*
MN527511 UAG CAAUCA
QSSRVNSRFRVLARRVEVPGIIPRCEFDAQGSLTLRSRSVPRVVKPRRLNPRLSKKEEEALDHFDQVDNSDGADYLSDWRSYVY*
MN527513 UAG CAAUCA
QSSRVNSRFRVLARRVEVPGIIPRCEFDAQGSLTSRSRSVPRVVKPRRLNPRLSKKEEEALDHFDQVDNSDGADYLNDWRSYVY*
MN527474 UAG CAAUCA
QSSRVNSRFRVLARRVEVPGIIPRCEFDAQGSLTSRSRSVPRVVKPRRLNPRLSKKEEEALDHFDQVDNSDGADYLNDWRSYVY*
MN527480 UAG CAAUCA
QSSRVNSRFRVLARRVEVPGIIPRCEFDAQGSSTLRSRSVPRVVKPRRLNPRLSKKEEEALDHFDQVDNSDGADYLSDWRSYVY*
MN527492 UAG CAAUCA
QSSRVNSRFRVLARRVEVPGNIPRCEFDAQGSSTLRSRSVPRVVKPRRLNPRLSKKEEEALDHFDQVDNSDGADYLNDWRSYVY*
MN527495 UAG CAAUCA
QSSRVNSRFRVFARRVEVPGIIPRCEFDAQGSLTLRSRSVPRVVKPRRLNPRLSKKEEEALDHFDQVDNSDGADYLNDWRSYVY*
MN527505 UAG CAAUCA
QSSRVKSRFRVLARRVEVPGIIPRCEFDAQGSLTLRSRSVPRVVKPRRLNPRLSKKEEEALDHFDQVDNSDGADYLSDWRSYVY*
MN527507 UAG CAAUCA
QSSRVNSRFRVLARRVEVPGNIPRCEFDAQGSSTLRSRSVPRVVKPRRLNPRLSKKEEEALDHFDQVDNSDGADYLNDWRSYVY*
MN527512 UAG CAAUUA
QLSRVNSRFRVLARRVEVPGIIPRCEFDAQGSLTSRSRSVPRVVKPRRLNPRLSKKEEEALDHFDQVDNSDGADYLSDWRSYVY*
MN527516 UAG CAAUCA
QSSRVNSRFRVLARRVEVPGNIPRCEFDAQGSSTLRSRSVPRVVKPRRLNPRLSKKEEEALDHFDQVDNSDGADYLNDWRSYVY*
MN527518 UAG CAAUCA
QSSRVNSRFRVFARRVEVPGIIPRCEFDAQGSLTSWSRSVPRVVKPRRLNPRLSKKEEEALDHFDQVDNSDGADYLNDWRSYVY*
MN527508 UAG CAACUA
QLSRVNSRFRVLARRVEVPGIIPRCEFDAQGSSTLRSRSVPRVVKPRRLNPRLSKKEEEALDHFDQVDNSDGADYLSDWRSYVY*
MG672022 UAG CAAUCA
QSSRVNSRFRVLARRVEVPGIIPRCEFDAQGSLTSRSRSVPRVVKPRRLNPRLSKKEEEALDHFDQVDNSDGADYLNDWRSYVY*
MN527471 UAG CAAUCA
QSSRVNSRFRVLARRVEVPGIIPRCEFDAQGSLTFRSRSVPRVVKPRRLNPRLSKKEEEALDHFDQVDNSDGADYLSDWRSYVY*
MN527483 UAG CAAUCA
QSSRVNSRFRVLARRVEVPGIIPRCEFDAQGSLTLRSRSVPRVVKPRRLNPRLSKKEEEALDHFDQVDNSDGADYLSDWRSYVY*
MN527486 UAG CAAUCA
QSSRVNSRFRVLARRVEVPGIIPRCEFDAQGSLTSRSRSVPRVVKPRRLNPRLSKKEEEALDHFDQVDNSDGADYLNDWRSYVY*
MN527489 UAG CAAUCA
QSSRVNSRFRVLARRVEVPGIIPRCEFDAQGSSTLRSRSVPRVVKPRRLNPRLSKKEEEALDHFDQVDNSDGADYLNDWRSYVY*
MN527506 UAG CAAUCA
QSGRVNSRFRVLARRVEVPGIIPRCEFDAQGSLTLRSRSVPRVVKPRRLNPRLSKKEEEALDHFDQVDNSDGADYLSDWRSYVY*
MN527509 UAG CAAUUA
QLGRVNSRFRVLARRVEVPGIIPRCEFDAQGSLTLRSRSVPRVVKPRRLNPRLSKKEEEALDHFDQVDNSDGADYLNDWRSYVY*
MN527510 UAG CAAUUA
QLGRVNSRFRVLARRVEVPGIVPRCEFDAQGSLTLRSRSVPRVVKPRRLNPRLSKKEEEALDHFDQVDNSDGADYLNDWRSYVY*
MN527515 UAG CAAUCA
QSSRVNSRFRVLARRVEVPGIIPRCEFDAQGSLTSRSRSVPRVVKPRRLNPRLSKKEEEALDHFDQVDNSDGADYLNDWRSYVY*
MN527517 UAG CAAUUA
QLSRVNSRFRVLARRVEVPGIIPRCEFDAQGSLTSRSRSVPRVVKPRRLNPRLSKKEEEALDHFDQVDNSDGADYLNDWRSYVY*
MN527477 UAG CAAUCA
QSSRVNSRFRVLARRVEVPGIIPRCEFDAQGSLTLRSRSVPRVVKPRRLNPRLSKKEEEALDHFDQVDNSDGADYLSDWRSYVY*

CLUSTER 6a

Alignment of 3'UTRs from the 3 segments of a representative genome

```
RNA2                UAAAUUGAAAGUUAGAAAGAGCGGGAGGCUCGAAGUUCUAAUUUGAGGAGCGUGGAGCUC
RNA3                UGAUGGAGCGAUUAGCUC---CGUCUCGCGUGAAA---------GAGGACUGUGAAGCUC
RNA1                UGAGCGGGAGGCUCGAUA----GUUUUAUGUGG----------GAGGAGCGUGAAGCUC
                    * *         * *       *        *            *****  *** *****

RNA2                GAAGUUCCGAGAGGGAACUGAAGCAUAUCGUGAGGAUAUGCGUUGUAGGG--GAGAUGGA
RNA3                GAAGUUCCGAGAGGGAACUAAAGCAUAUCGCGAGGAUAUGCGAUGUAGGGGUGAGAUGGA
RNA1                GAAGUUCCGAGAGGGAAGUGAAGCAUGUCGUGAGGAUAUGCAGUGUAGGG--GAGAUGGA
                    ***************** * ****** *** **********  *******  ********

RNA2                CCCCAAGACUCGAUUAGAGUCAGACCGUACCACCGCUAGUGGAUUUGCGGUCAGUUUUCC
RNA3                CCCCGAGACUCGAUUAGAGUCAGACCGUACCACCGAUAGUGGGUUUGCGGUCAGUUUUCC
RNA1                CCCCGAGACUCGAUGAGAGUCAGACCGUACCACCGAUUGUGGGUUGGCGGUCAGUUUGCC
                    **** ********* ******************** * **** ** *********** **

RNA2                GAGCGUCGCCUCCUUAGGCGUGUCCUAGGAAAACAACAACUCAGUGUGGAGUUGAUUCCA
RNA3                GAGCGUCGCCUCCUUAGGCGUGUCCUAGGAAAACAACAACUCGAUGUAGAGUUGAUUCCA
RNA1                GAGCGUCGCCGCGUUAGGCGUGUCCUAGGAAGACAACAACUCGACGUAGAGUUGAUGCCA
                    ********** * ****************** **********   ** ******** ***

RNA2                AUUUCUGUGAAAGAAAUUGAUGCCCCGAUUAGGAGGC
RNA3                AUUUCUGUGAAAGAAAUUGAUGCCCCGAUUAGGAGGC
RNA1                AUGUCUGUGAAAGAAAUUGAUGCCCCGAUUAGGAGGC
                    ** **********************************
```

RNA1 NC_003464 260 nt
RNA2 NC_003465 275 nt
RNA3 NC_003480 265 nt

Note: No insert in RNA3, so no evidence for RT domain.

All sequences in the cluster

    12 Apple mosaic virus

Accession number / CP stop and 3'-adjacent 6 nt / CP-frame translation of sequence from immediately after CP stop codon to next in-frame stop codon (*), if present, or to end of available sequence

```
L03726    UGA UGGAGC WSD*
KY971021  UGA UGGAGC WSG*
MT303163  UGA UGGAGC WSD*
HG328281  UGA UGGAGC WSD*
HG328280  UGA UGGAGC WSD*
KY971019  UGA UGGAGC WSD*
MN822139  UGA UGGAGC WSD*
KY883320  UGA UGGAGC WSD*
KY488189  UGA UGGUGC WCQLVPLARKWTVKLDVPRGNLSX
HE574164  UGA UGGAGC WSD*
NC_003480 UGA UGGAGC WSD*
KY971020  UAG UGUGGG CGMSVKLEVPRGN*
```

CLUSTER 6b

A subgroup of apple mosaic virus sequences (see below) have a longer downstream ORF and a UAG CAA UCA or UAG CAA UUA (consistent with a UAG CAR YYA readthrough motif). In the absence of RNA1/RNA2 sequences for the corresponding genomes, below is an alignment between a representative Cluster 6b sequence (HG328285) and a representative Cluster 6b sequence (NC_003480). Note the clear insert immediately following the CP stop codon, indicating a RT domain in Cluster 6b sequences. However, this appears to have evolved independently of the RT Zn-finger domain in other ilarviruses.

```
HG328285        CCACCUGUGCAAAUACCGUCGAAUGUUCUACUUAUCGAUGAAGAUCUGUUAGAUUUAUAG
NC_003480       CCACCGGUGCAAAUACCGACGAAUGUUCUACUUAUCGAUGAAGAUUUGUUAGAAUUA---
                ***** ************ ************************** ******* ***

HG328285        CAAUCAGGAAGUCAGAUUUCAAAUAUCGUGAGGAUAUUUGAUAAUCUAGACGAUUUGAUG
NC_003480       -------------------------------------------------------UGAUG
                                                                       *****

HG328285        GAGAGAUUUGCUCCGU-----AUGCAGAAGGAGCGUGAAGCUCGAAGUUCCGAGAAGGAA
NC_003480       GAGCGAUUAGCUCCGUCUCGCGUGAAAGAGGACUGUGAAGCUCGAAGUUCCGAGAGGGAA
                *** **** *******      ** *  ****  ********************* ****

HG328285        CUGAAAUAUAUCGAGAGGAUAUAUGUUGUAGGG--GAAAAGGACCCCGAGACUCGAUUAG
NC_003480       CUAAAGCAUAUCGCGAGGAUAUGCGAUGUAGGGGUGAGAUGGACCCCGAGACUCGAUUAG
                ** **  ****** ********  * *******  ** * ********************

HG328285        AGUCAGACCGUACCACCAUUAGUGGGUUUGCGGUCAGUUUUCUGAGCGUCGUCUCCUUAG
NC_003480       AGUCAGACCGUACCACCGAUAGUGGGUUUGCGGUCAGUUUUCCGAGCGUCGCCUCCUUAG
                *****************  *********************** ******** ********

HG328285        ACGUGUCCUAAGAAAACAACAACUCGAUAUAGAG--------------------------
NC_003480       GCGUGUCCUAGGAAAACAACAACUCGAUGUAGAGUUGAUUCCAAUUUCUGUGAAAGAAAU
                 ********* ***************** *****

HG328285        --------------------
NC_003480       UGAUGCCCCGAUUAGGAGGC
```

All sequences in the cluster

```
     8 Apple mosaic virus
```

Accession number / CP stop and 3'-adjacent 6 nt / CP-frame translation of sequence from immediately after CP stop codon to next in-frame stop codon (*), if present, or to end of available sequence

```
AM490197  UAG CAAUCA QSRGQGPNIVKIFDNLDELMERLAPYAEGA*
HG328285  UAG CAAUCA QSGSQISNIVRIFDNLDDLMERFAPYAEGA*
HG328282  UAG CAAUCA QSRGQNSNIVKIFDNLDDLMERFAPYAEGA*
HG328283  UAG CAAUCA QSGGQISNIVRIFDNLDVLMERFAPYAEGA*
HG328284  UAG CAAUCA QSGGQISNIVRIFDNLDVLMERFAPYAEGA*
HG328276  UAG CAAUCA QSRGQNSNIVKIFDNLDELMERX
HG328279  UAG CAAUUA QLRGQNSNIVKIFDNLDELMERX
HG328277  UAG CAAUUA QLRGQTSNIVKIFDNLDELMERX
```

CLUSTER 7

Alignment of 3'UTRs from the 3 segments of a representative genome

```
RNA1            UAGGUU-CGAUG--CAGAACUGAACCCCGAUUAGGGUAGAACCGGACCACCGUGUGGGUU
RNA2            UAGGUUGAGAUGAACACAACCGAACCCCGAUAAGGGUAGAACCGGACCACCGUGUGGGUU
RNA3            UAGGUUGAGAUGAACACAACCGAACCCCGAUAAGGGUAGAACCGGACCACCAUGUGGGUU
                ******  ****  ** *** ********** ******************* ********

RNA1            UU---CCGGUUAUCUCCGAGAGUCGCUUUCCAGACUCAUGCUA-AGGAGAUUACCUACGA
RNA2            UUCCACCGGUUGUCUCCGAGAGUCGCUUUCCAGACUCGUGGUUUAGGAGAUUACCUACGA
RNA3            UU---CCGGUUAUCUCAGAGAGUCGCUUUCCAGACUCGUGUUCUAAGAGAUUACCUACGA
                **   ****** **** ******************** ** *  * **************

RNA1            AAGUAGAUGCCCGAUCCGUGAAGGAUCGGAUGCCCCGUUAGGGAAGC-
RNA2            AAGUAGAUGCCCAAUCCGUGAAGGAUUGGAUGCCCCGUUAGGGAAACC
RNA3            AAGUAGAUGCCCAAUCCGUGAAGGAUUGGAUGCCCCGUUAGGGAACC-
                ************ ************* ****************** *
```

RNA1 NC_040469 160 nt
RNA2 NC_040471 168 nt
RNA3 NC_040470 164 nt

Note: No insert in RNA3, so no evidence for RT domain.

All sequences in the cluster

```
     15 Apple necrotic mosaic virus
```

Accession number / CP stop and 3'-adjacent 6 nt / CP-frame translation of sequence from immediately after CP stop codon to next in-frame stop codon (*), if present, or to end of available sequence

```
MN832845  UGA GCACAA AQPNPDKGRTGPPCGFSGYLRESLLRLKLRRLPTKVDAQSVKDWMPR*
MN911185  UAG GUUGAG VEMNTTEPR*
KY808384  UAG GUUGAG VEMNTTEPR*
MN911186  UAG GUUGAG VEMNTTEPR*
KY808390  UAG GUUGAG VEMNTTEPR*
MG924901  UAG GUUGAG VEMNTTEPR*
KY808378  UAG GUUGAG VEMNTTEPR*
KY808381  UAG GUUGAG VEMNTTEPR*
NC_040470 UAG GUUGAG VEMNTTEPR*
MN656200  UAG GUUGAG VEMNTTEPR*
KY808387  UAG GUUGAG VEMNATEPR*
MN023212  UAG GUUGAG VEMNTTEPR*
KY808393  UAG GUUGAG VEMNTTEPR*
MG924900  UAG GUUGAG VEMNTTEPR*
MG924902  UAG GUUGAG VEMNTTEPR*
```

Note that the UGA (instead of UAG) CP stop codon and extended downstream peptide of sequence MN832845 are the result of a 4-nt frame-changing deletion at the end of the CP ORF (see alignment below). This could be a real mutation or could be a sequencing error. There is no large insertion and therefore no evidence for a RT domain in this sequence.

UAG CP stop codon in NC_040470
UGA Apparent CP stop codon in MN832845

```
MN832845        CGAAUCGUGAUCCCGAGUGACAUUCUAAUGGUCGAUGAAGACCUUUUGGAUGU----GUU
NC_040470       CGAAUCGUGAUCCCGAAUGAUAUCCUUAUGGUCGAUGAAGACCUUUUGGAUGUCUAGGUU
                **************** *** ** ** **************************    ***

MN832845        GAUAUGAGCACAACCGAACCCCGAUAAGGGUAGAACCGGACCACCUUGUGGGUUUUCCGG
NC_040470       GAGAUGAACACAACCGAACCCCGAUAAGGGUAGAACCGGACCACCAUGUGGGUUUUCCGG
                ** **** ************************************* **************

MN832845        UUAUCUCCGAGAGUCGCUUCUCAGACUCAAGCU-UAGGAGAUUACCUACGAAAGUAGAUG
```

```
NC_040470       UUAUCUCAGAGAGUCGCUUUCCAGACUCGUGUUCUAAGAGAUUACCUACGAAAGUAGAUG
                ******* ***********  *******  * * ** ***********************

MN832845        CCCAAUCCGUGAAGGAUUGGAUGCCCCGUUAGGGAUG-
NC_040470       CCCAAUCCGUGAAGGAUUGGAUGCCCCGUUAGGGAACC
                ***********************************
```

CLUSTER 8a

Alignment of 3'UTRs from the 3 segments of a representative genome

```
RNA1            ------------------------------------------------------------
RNA2            ------------------------------------------------------------
RNA3            UGACUACACUCGGACGAUUGUCCGAGUCAAAGUGUCAUCCCUUUCUGUCAUUGCGAAUGU

RNA1            ------------------------------------------------------------
RNA2            ------------------------------------------------------------
RNA3            UAUGACAAUGCUUGCGGUACACCUUGGUGUCCUGCUAAUGCCAAUUGUUUCCAUGAAUUU

RNA1            ------------------------------------------------------------
RNA2            ---------------UGAUGUGUUCUUUCAUGGAAUCUAGCAAGGAGCCCUCAGGGGUGC
RNA3            CAUGAAAAUUAUUGAUGACGUGUUCUUUCAUAGAAUCUAGCAAGGAGCCCUCAGGGGUGC

RNA1            --------------------UAAGUCCAUAUGCCCAUCUCUGCUACUCCGGAUGGAUGUC
RNA2            ACUCAGUGCCUAGUCCACGCUAAGUCUAUAUGCCCACCUUUGCUACUCCGGGUGGAUGCU
RNA3            ACUCAGUGCCUAGUCUACGCUAAGUCUAUAUGCCCACCUUUGCUACUCCGGGUGGAUGUU
                                    ****** ********* ** *********** ******

RNA1            UAUAUGCCCGCUAUGGAUGCCUAUUAUUGAAAUAUAAUAGAUGCCUAAUACUCUCUCUCA
RNA2            U-UAAGUGCA-UGUAGAUGCCUAUAUUUGAAAUAAUAUAGAUGCCCAG-ACUCUCUCUCU
RNA3            C-UAAGUGUA-UAUAGAUGCCUAUAUUUGAAAUAAUAUAGAUGCCCAA-ACUCUCUCUCA
                  ** *     * * *********  ********  ********* *  **********

RNA1            GGGAGAGAGUUUAGAUGCCUCCGAAGGAGAUGC
RNA2            UGGAGAGAGAAUGGAUGCCUCCGAAGGAGAUGC
RNA3            UGGAGAGAGAAUGGAUGCCUCCGAAGGAGAUGC
                 ********  * ********************
```

RNA1 NC_009537 133 nt
RNA2 NC_009538 195 nt
RNA3 NC_009536 330 nt

All sequences in the cluster

4 Citrus variegation virus

Accession number / CP stop and 3'-adjacent 6 nt / CP-frame translation of sequence from immediately after CP stop codon to next in-frame stop codon (*), if present, or to end of available sequence

```
NC_009536 UGA CUACAC LHSDDCPSQSVIPFCHCECYDNACGTPWCPANANCFHEFHENY*
MZ330111  UGA CUACAC LHSDDCPRQCVISFCHCECYDNACSTPWCPANTNCFHEFHEDY*
MZ330094  UGA CUACAC LHSDDCPRQSVISFCHCECYDNACSTPWCPANANYFHEFHEDY*
MZ330098  UGA CUACAC LHSDDCPRQCVISFCHCECYDNACSTPWCPANANYFHEFHEDY*
```

CLUSTER 8b

Alignment of 3'UTRs from the 3 segments of a representative genome

```
RNA1            ------------------------------------------------------------
RNA2            ------------------------------------------------------------
RNA3            UGACUACACUCGGGUGAUUACCUCCAUUCUGUUGAAUGUGAGAGGAAGUGUGUGAUUCCA

RNA1            ------------------------------------------------------------
RNA2            ------------------------------------------------------------
RNA3            UUCUGUCAUUGCGUGUGCUAUGACGAUGCUUGCGAUGUUCUUUGGUGUUCCCCAAAUGAU

RNA1            ---------------------------------UAGUGUUUCUUUCAUGAAACCUAGUGA
RNA2            ---------------------------------UAGUGUUUCUUUCAUGAAACCUAGUGA
RNA3            UUAUGUUACCACGAGUCUAAUGACUGGUGAUGUUAGUGUUUCUUUCAUGAAACCUAGUGA
                                                 ***************************

RNA1            AGGGUCCUCCGGGAUGUGCUCAGCACCUAGCCUAAGCUAAGUCCAUAUGCCCAUCUUUGC
RNA2            AGGGUCCUCCGGGAUGUGCUCAGCACCUAGCCUAAACUAAGUCCAUAUGCCCACCUUUGC
RNA3            AGGAUCCUCCGGGAUGUGCUCAGCACCUAGCCUAAACUAAGUCCAUAUGCCCAUCUUUGC
                *** ******************************* ***************** ******

RNA1            UGCUCCGGAUGGAUGUUUAUACCCGCUAUGGAUGCCUAUUAUUG-AAUAUAAUAGAUGCC
RNA2            UGCUCCGGAUGGAUGUUAAUACCCGCUAUGGAUGCCUAUUAUUGAAAUAUAAUAGAUGCC
RNA3            UGCUCCGGAUGGAUGUUAAUACCCCCUAUGGAUGCCUAUUAUUGAAAAAUAAUAGAUGCC
                ***************** ****** ******************* ** ************

RNA1            UAAUUCUCUCUCUCAGGGAGAGAGAUUAGAUGCCUCCCAGGAGAUGC
RNA2            UAAUUCUCUCUCUCAGGGAGAGAGAUUAGAUGCCUCCAAGGAGAUGC
RNA3            UAAUUCUCUCUCUCAGGGAGAGAGAUUAGAUGCCUCCAAGGAGAUGC
                ************************************* *********
```

RNA1 NC_003569 193 nt
RNA2 NC_003568 194 nt
RNA3 NC_003570 347 nt

All sequences in the cluster

```
      4 Elm mottle virus
      1 Hydrangea mosaic virus (AF172965)
```

Accession number / CP stop and 3'-adjacent 6 nt / CP-frame translation of sequence from immediately after CP stop codon to next in-frame stop codon (*), if present, or to end of available sequence

```
LT898352   UGA CUACGC LRSGDYLHSIECERKSVIPFCHCICYDNACDVLWCSANDLCYHESNDW*
OL584365   UGA CUACGC LRSGDYLHSIECERKSVIPFCHCICYDNACDVLWCSANDLCYHESNDW*
AF172965   UGA CUACAC LHSGDYLHSVECERKCVIPFCHCVCYDDACDVLWCSANDLCYHESNDW*
NC_003570  UGA CUACAC LHSGDYLHSVECERKCVIPFCHCVCYDDACDVLWCSPNDLCYHESNDW*
U57048     UGA CUACAC LHSGDYLHSVECERKCVIPFCHCVCYDDACDVLWCSANDLCYHESNDW*
```

CLUSTER 9

Alignment of 3'UTRs from the 3 segments of a representative genome

```
RNA1          UGAUAGAAGUGAUUUGAUCACACUUCGAAUAGGGUUU-GUAACCCGAAUACCGGUCCUCC
RNA2          ---------UGAUUUCAUCACACUUCG--UAGGGCCU---AGGCCCAUUACCGGUCCUCC
RNA3          ---UAGAAGUGAUUUGAUCACACUUCGAUUAGAACCGUGAAGGUUCGAUACCGGUCCUCC
                       ****** ***********  ***        *       ************

RNA1          UUGAGGAUUUCCCGGUAGAGUCUAUAAGAUUUCGCCUUCCGAAAUCGACGAUCUAUAGAU
RNA2          GUGAGGAUUUCCCGGUAGAAUCUAAUAGAUCUUGCCUCUCAAGAUCUGUGAUGGUUAGAU
RNA3          GUGAGGAUUUACCGGUAGAAUCUAUUGUAUCUCGCCUC-CGAGAUACAUGAUCGAUAGAU
               ********* ******** ****    ** * ****  * * **    ***   *****

RNA1          UCAACAACCGAUAUGGUUGAUUCCUAAUUCUGUGAAAGAAUUAGAUGCCCCGAUAGGGA
RNA2          UCAACAAUCGUUAUGAUUGAUUCCUAAUUCCGUGAAGGAAUUAGAUGCCCCGAUAGGGA
RNA3          UCAACAACCGAUAUGGUUGAUUCCUAAUUCCGUGAAGGAAUUAGAUGCCCCGACAGGGA
              ******* ** **** ************** ***** **************** *****
```

RNA1 NC_022250 178 nt
RNA2 NC_022251 165 nt
RNA3 NC_022252 175 nt

Note: No insert in RNA3, so no evidence for RT domain.

All sequences in the cluster

      7 Blueberry shock virus

Accession number / CP stop and 3'-adjacent 6 nt / CP-frame translation of sequence from immediately after CP stop codon to next in-frame stop codon (*), if present, or to end of available sequence

```
KY927866  UAG AAGUGA K*
KF031042  UAG AAGUGA K*
NC_022252 UAG AAGUGA K*
KY927865  UAG AAGUGA K*
KY927862  UAG AAGUGA K*
KY927864  UAG AAGUGA K*
KY927863  UAG AAGUGA K*
```

CLUSTER 10

Alignment of 3'UTRs from the 3 segments of a representative genome

```
RNA1            UGAU------UAAUUUCAAGUCCACAUAGUGAU---------------------------
RNA2            UAAUGGCAACCGAUC---------------------------------------------
RNA3            UGACGGUCACAAGUCCCGAGUCCAAGACUCGAGUUGCCGUACUUCACUGAAAGUGUGUA
                * *          *

RNA1            ------------GCCCCAU---------UUGGGAAGAGAACUCUGGAAGG----------
RNA2            ------------GCAUC-----------UUAAUCGGUGUAUU------------------
RNA3            CGAUGCGGGUUUGCACCAAAGUAUACUGUAGAUCAGUAUAUUUGGAAGUGUCACGUGUGU
                            **  *           *      *   * *

RNA1            ------------------CACUUCUCACGCUUCUAAGAGAAGCCCA-GUGUUCUAAUACC
RNA2            ---------------------------------------GAGUUUA---AUUCUAC----
RNA3            UCUUAUGAACACUACGCAUAGUUGUCACGCACCGGGCGUAAAUCUAUGAAUUCCGCUAUU
                                                        *    *    ***

RNA1            AA----------------------------------------------------------
RNA2            ------------------------------------------------------------
RNA3            AAGUGGUCCGUAUUGGACUAUACCUUGAAUCGUAAGAUUCAUGAGGCCUUUGGGCCGCCA


RNA1            --------------UACUACCUAAUGGUAGUAUUGAUGCCUCCAAAAGGAGAAG
RNA2            --------------UAGUAUCUAAUGAUACUAGUAAUGCCUCCAAAAGGAGAUG
RNA3            CUUAAGAACGAUGCUACUAGUAUAUAAUACUAGUAAUGCCUCCAAAAGGAGAUG
                              ** **    **  ** ** * ***************** *
```

```
RNA1 OL472060 139 nt
RNA2 OL472061  88 nt
RNA3 OL472062 294 nt
```

All sequences in the cluster

```
      4 Solanum nigrum ilarvirus 1
      1 Surrounding legume associated ilarvirus (MN412727)
      1 Grapevine associated ilarvirus (MN520744)
      1 Erysiphe necator associated ilar-like virus 1 (MN630188)
```

Accession number / CP stop and 3'-adjacent 6 nt / CP-frame translation of sequence from immediately after CP stop codon to next in-frame stop codon (*), if present, or to end of available sequence

```
MN520744  UGA CGGUCA RSQVPSPRLELPYFTEKCVRCGFAPKYTVDQYIWKCHVCSYEHYA*
MN412727  UGA CGGUCA RSQVPSPRLELPYFTEKCVRCGFAPKYTVDQYIWKCHVCSYEHYA*
OL472062  UGA CGGUCA RSQVPSPRLELPYFTEKCVRCGFAPKYTVDQYIWKCHVCSYEHYA*
MN216376  UGA CGGUCA RSQVPSPRLDLPYFTEKCVRCGFAPKYTVDQYIWKCHVCSYEHYA*
MN216377  UGA CGGUCA RSQVPSPRLELPYFTEKCVRCGFAPKYTVDQYIWKCHVCSYEHYA*
MN216378  UGA CGGUCA RSQVPSPRLELPYFTEKCVRCGFAPKYTVDQYIWKCHVCSYEHYA*
MN630188  UGA CGGUCA RSQVPSPRLELPYFTEKCVRCGFAPKYTVDQYIWKCHVCSYEHYA*
```

CLUSTER 11

Alignment of 3'UTRs from the 3 segments of a representative genome

```
RNA3            UGACUGUCUCCAUCCUCGAGUUUGAUCUCGAGUUGCCUCACAUUAAGAAUAAUUCUUGUG
RNA1            --------------------------------------------------------UGAG
RNA2            ------------------------------------------------------------


RNA3            UGAGAUGCGGAUAUCACCCUAUGUAUGUUGUAGAUCAACAUAUAUGGAAGUGUGAUGUGU
RNA1            CGCAAUGCG-------------CUAUUUAUUAUUCCAACAAGAAG---------------
RNA2            -----------------------------------UAACAUG------------------
                                                    ****

RNA3            GUCAUCAAAGACACUAUGCAUAGUUACCACGCAUCCGGGUGUAAAUCUAUGUUAUCCGUU
RNA1            ------------------------------GCGUCUGAGCGC--AUCUUUCUCAGAUCUU
RNA2            ------------------------------GCAGCUGAGCGC--AUCUAUCUCAGUGGUU
                                              **  * * * *   **** * * *    **

RNA3            UAGAACAUUCCGAAUCGGACGAUUUCCACUCUAUGAGUGAGACCUUUGGGUCGAUGUUCU
RNA1            GUUGAUGUUUCCAAU-----AAUAUCAA--------------------------------
RNA2            AAUGA--UUGCUAUU-----AAUAUCUA--------------------------------
                    *  ** * * *      ** ** *

RNA3            AUGUUGCCAUUAAUAAUUUAAAAUUAUUAAUGAUGCCUCCCUAAGGAGAUGC
RNA1            -----------------UUGAUAUUAUUGAUGCCUCUAAUUUAUAGAGAUGC
RNA2            -----------------AUGAUAUUAAUAAUGCCUCCAAUUU--GGAGAUGC
                                  * * **** * ***   *     *   *******
```

```
RNA1 NC_008708 122 nt
RNA2 NC_008707  89 nt
RNA3 NC_008706 292 nt
```

All sequences in the cluster

```
      5 Strawberry necrotic shock virus
```

Accession number / CP stop and 3'-adjacent 6 nt / CP-frame translation of sequence from immediately after CP stop codon to next in-frame stop codon (*), if present, or to end of available sequence

```
NC_008706 UGA CUGUCU LSPSSSLISSCLTLRIILV*
KU053514  UGA CUAUCU LSPILESNLELPHIKGNSCVRCGYHPMYVVDQHIWTCDVCRQRQYA*
JF781586  UGA CUAUCU LSPILESNLELPHIKGNSCVRCGYHPMYVVDQHIWTCDVCRQRQYA*
JF781587  UGA CUAUCU LSPILESNLELPHIKGNSCVRCGYHPMYVVDQHIWTCDVCRQRQX
JF781588  UGA CUAUCU LSPILESNLELPHIKGNSCVRCGYHPMYVVDQHIWTCDVCRQRQX
```

The truncated downstream peptide in NC_008706 occurs as a result of a single nucleotide deletion 9 nt downstream of the stop codon:

```
NC_008706       UGACUGUCUCC-AUCCUCGAGUUUGAUCUCGAGUUGCCUCACAUUAAGAAUAAUUCUUGU
KU053514        UGACUAUCUCCAAUCCUCGAGAGUAAUCUCGAGUUGCCUCACAUUAAAGGUAAUUCUUGU
JF781586        UGACUAUCUCCAAUCCUCGAGAGUAAUCUCGAGUUGCCUCACAUUAAAGGUAAUUCUUGU
JF781587        UGACUAUCUCCAAUCCUCGAGAGUAAUCUCGAGUUGCCUCACAUUAAAGGUAAUUCUUGU
JF781588        UGACUAUCUCCAAUCCUCGAGAGUAAUCUCGAGUUGCCUCACAUUAAAGGUAAUUCUUGU
                ***** ***** *********  * **********************   **********

NC_008706       GUGAGAUGCGGAUAUCACCCUAUGUAUGUUGUAGAUCAACAUAUAUGGAAGUGUGAUGUG
KU053514        GUGAGAUGCGGAUAUCACCCUAUGUAUGUUGUAGAUCAACAUAUAUGGACGUGUGACGUA
JF781586        GUGAGAUGCGGAUAUCACCCUAUGUAUGUUGUAGAUCAACAUAUAUGGACGUGUGACGUA
JF781587        GUGAGAUGCGGAUAUCACCCUAUGUAUGUUGUAGAUCAACAUAUAUGGACGUGUGACGUA
JF781588        GUGAGAUGCGGAUAUCACCCUAUGUAUGUUGUAGAUCAACAUAUAUGGACGUGUGACGUA
                ************************************************* ****** **
```

If the deletion in NC_008706 is replaced with an "A", the RT peptide is restored:

```
NC_008706 LSPILEFDLELPHIKNNSCVRCGYHPMYVVDQHIWKCDVCHQRHYA*
```

```
KU053514  LSPILESNLELPHIKGNSCVRCGYHPMYVVDQHIWTCDVCRQRQYA*
JF781586  LSPILESNLELPHIKGNSCVRCGYHPMYVVDQHIWTCDVCRQRQYA*
JF781587  LSPILESNLELPHIKGNSCVRCGYHPMYVVDQHIWTCDVCRQRQX
JF781588  LSPILESNLELPHIKGNSCVRCGYHPMYVVDQHIWTCDVCRQRQX
```

CLUSTER 12

Alignment of 3'UTRs from the 3 segments of a representative genome

```
RNA1            UAACCAUUUCGUAU-----------------------CGUGAGAUACGAUACCUCCUUA
RNA2            ------------------------------------------------------------
RNA3            UGAAUGGUUGUUACCGACGUUUCAGGCGUCGAUGCCAACGUAAAGAGUGAUGACUCCUAU


RNA1            AGGGAGAUGUUUUUCCACAAUGA-------------------------------------
RNA2            ------------------------------------------------------------
RNA3            CAAGACGGAGAUUAUUAUUAUGAUACCGACAUGUUCCAGUAUGAUUGUACUGACGAUGAU


RNA1            -----------------------------------------------------------G
RNA2            ----------------------------------------------------------UA
RNA3            GACGGUACAGCCUACAUGCAAGAUUUCUUCUCGUGUGUAGCUGCCAUGAAUUUCUAAUUG


RNA1            AGUUGUGAUGCCCCAAUAGUGGGAAGAACGUUCGGGUGGCUACUUAAGCGCUUCUAUACU
RNA2            GGUUGUGAUGCCUCCAU-GUGAGAAGGUCAUUCGGGUGGCUACUUAAGCGCUUCUAUACU
RNA3            AUUCAUGAUGCCUCCUU-GUGAGAAGGAUGUUCGGGUGGCUGCUUAAGCGCUUCUAUACU
                  *  ******* *  * *** ****    *********** ******************

RNA1            UAAGUACCGAACGCUGUUCCGUUAUUAUCUAGUGACAAUAACGAUGCCUAUUUCUAUGAA
RNA2            UAAGUACCGAAUGUUAUACCGUCAUUAUCUAGUGAUAAUAACGAUGCCUAUUUCUAUGAA
RNA3            UAAGUACCGAAUGUUAUACCGUUAUUAUCUAGUGAUAAUAACGAUGCCUAUUUCUAUGAA
                *********** * * * **** ************ ************************

RNA1            AUAGAAGC
RNA2            AUAGAAGC
RNA3            AUAGAAGC
                ********
```

RNA1 NC_005848 188 nt
RNA2 NC_005849 129 nt
RNA3 NC_005854 307 nt

RNA3 has an insert consistent with the length of the downstream peptide, suggesting that RT occurs. On the other hand, the sequences lack a canonical RT motif (UGA A... instead of UGA C...) and the RT peptide lacks a Zn-finger.

All sequences in the cluster

4 Parietaria mottle virus
1 Tomato necrotic spot virus (MH780156)

Accession number / CP stop and 3'-adjacent 6 nt / CP-frame translation of sequence from immediately after CP stop codon to next in-frame stop codon (*), if present, or to end of available sequence

```
MH780156  UGA AUGGCU MAASDVSGVDATLSDDSDQDGDCYDAIFSQCDDTDDDGTAYMQDFFSCVDAMSF*
FJ858204  UGA AUGGUU MVVTDGSGVDATL*
KT005245  UGA AUGAUU MIVTDVSGVDANVKSDDSYQDGDYYYDTDMFQYDYTDDDDGTAYMQDFFSCVAAMNF*
MZ405648  UGA AUGGUU MVVTDVSGVDANVKSDDSYQDGDYYYDTDMFQYDCTDDDDGTAYMQDFFSCVAAMNF*
NC_005854 UGA AUGGUU MVVTDVSGVDANVKSDDSYQDGDYYYDTDMFQYDCTDDDDGTAYMQDFFSCVAAMNF*
```

The truncated downstream peptide in FJ858204 is due to an A to U substitution not an indel:

```
MH780156        UGAAUGGCUGCUUCCGACGUUUCAGGCGUCGAUGCCACUUUA---AGUGAUGACUCCGAU
FJ858204        UGAAUGGUUGUUACCGACGGUUCAGGCGUCGAUGCCACCCUAUAGAGUGAUGACUCCUGU
KU005245        UGAAUGAUUGUUACCGACGUUUCAGGCGUCGAUGCCAACGUAAAGAGUGAUGACUCCUAU
MZ405648        UGAAUGGUUGUUACCGACGUUUCAGGCGUCGAUGCCAACGUAAAGAGUGAUGACUCCUAU
NC_005854       UGAAUGGUUGUUACCGACGUUUCAGGCGUCGAUGCCAACGUAAAGAGUGAUGACUCCUAU
                ******  ** * ****** *****************   **   ************  *
```

CLUSTER 13

Alignment of 3'UTRs from the 3 segments of a representative genome

```
RNA1            ------------------------------------------------------------
RNA2            ------------------------------------------------------------
RNA3            UGACUACACUCGGACGAUUGUUCGAGUAGAAAUGUCAUUCCUUUUUGCCAUUGCUCAUGU


RNA1            --------------UAGUG-----------------------------------------
RNA2            --------------UGAUGUG---------------------------------------
RNA3            UGCCCUUUUCACUGUGAUGUGAUAGGAUGUGUGUGCAAUGAUGCCUUCUUUUACGAAGAU
                              *  **

RNA1            ----UUUUAUUUAUAAAACCUGGUUAAGGGUCCUCCGGGAUGUGCUCAGCACCUAGCCUA
RNA2            ----UUCCUUUCAUGGAACCUAGUGAGGGGCCCUCCGGGGUGUGCUCAGCACCUAGCCUA
RNA3            UAGUGUUCCUUCAUGGAACCUGGUAAGAGGUCCUCCGGGAUGUGCUCAGCACCUAGCUUA
                     *   ** **  ***** ** *  ** ******** ***************** **

RNA1            AGCCAAGUCCAUAUGCCCACCUAUGCUGCUCCGGGUGGAUGCUUAUACAUGCUAUGGAUG
RNA2            AGCUAAGUCCAUAUGCCCACCUACGCUGCUCCGGGUGGAUGCUUAUACAUGCUAUGGAUG
RNA3            AGCCAAGUCCAUAUGCCCACCUUCGCUGCUCCGGGUGGAUGCUUAUACAUGCUAUGGAUG
                *** ******************  ************************************

RNA1            CCUACAUUUGAAAUAAUGUAGAUGCCUAAUACUCUCUCUCAGGGAGAGAGUUUAGAUGCC
RNA2            CCUACAUUUGAAAUAAUGUAGAUGCCUAAUACUCUCUCUCAGGGAGAGAGUUUAGAUGCC
RNA3            CCUACAUUUGAAAUAAUGUAGAUGCCUAAUACUCUCUCUCAGGGAGAGAGUUUAGAUGCC
                ************************************************************

RNA1            UCCAAAGGAGAUGC
RNA2            UCCAAAGGAGAUGC
RNA3            UCCAAAGGAGAUGC
                **************


RNA1 NC_003808 195 nt
RNA2 NC_003809 197 nt
RNA3 NC_003810 314 nt
```

All sequences in the cluster

```
      4 Spinach latent virus
```

Accession number / CP stop and 3'-adjacent 6 nt / CP-frame translation of sequence from immediately after CP stop codon to next in-frame stop codon (*), if present, or to end of available sequence

```
KY695014  UGA CUACAC LHSDDCSSRNVIPFCHCSCCPFHCDVIGCVCNDAFFYED*
MT269815  UGA CUACAC LHSDDCSSRNVIPFCHCSCCPFHCDVIGCVCNDAFFYED*
OK044131  UGA CUACAC LHSDDCSSRNVIPFCHCSCCPFHCDVIGCVCNDAFFYED*
NC_003810 UGA CUACAC LHSDDCSSRNVIPFCHCSCCPFHCDVIGCVCNDAFFYED*
```

CLUSTER 14

Alignment of 3'UTRs from the 3 segments of a representative genome

```
RNA2            ------------------------------------------------------------
RNA1            ------------------------------------------------------------
RNA3            UGACCACGCUCGGGUGAGUGUGUAUACCUCCAUUCUAAUGAAUGUGAGAAGAAAAAUGUG


RNA2            ------------------------------------------------------------
RNA1            ------------------------------------------------------------
RNA3            AUUCCAUUCUGUCAUUGUGAAUGUUAUGACAAUGCUUGCAACACCUUGUGGUGUCCUGCA


RNA2            -------------------------------------UGACGUGUUUCUUUCAUGAAACC
RNA1            ------------------------------------------------------------
RNA3            AAUGUCUUUUGUUUCCACGAACCUUAAUGGUUAGUGAUGAAGUGUUUCUUUCAUGAAACC


RNA2            UAGUGAAGGGUCCUCCGGGAUGUGCUCAGCACCUAGCCUAAGCUAAGUCCAUAUGCCCAU
RNA1            -------------------------------------------UAAGUCCAUAUGCCCAU
RNA3            UAGUGAAGGGU-CUCCGGGAUK-GCUCAGCACCUAGCCUAAGCUAAGUCCAUAUGCCCAU
                                                           *****************

RNA2            CUUCGCUGCUCCGGAUGGAUGUUUAUACCCGCUAUGGAUGCCUAUUACUGAAAUGUAAUA
RNA1            CUUUGCUGCUCCGGAUGGAUGUUUAUACCCGCUGUGGAUGCCUAUUACUGAAAUGUAAUA
RNA3            CUUUGCUGCUCCGGAUGGAUGUUUAUACCCGCUAUGGAUGCCUAUUACUGAAAUGUAAUA
                *** **************************** ***************************

RNA2            GAUGCCUAAUACUCUCUCUCAGGGAGAGAGUUUAGAUGCCUCCAAAGGAGAUGC-
RNA1            GAUGCCUAAUACUCUCUCUCAUGGAGAGAGUUUAGAUGCCUCCAAAGGAGAUGC-
RNA3            GAUGCCUAAUACUCUCUCUCAGGGAGAGAGUUUAGAUGCCUCCAAAGGAGAUGCU
                ********************* *******************************
```

RNA1 NC_011808 131 nt
RNA2 NC_011809 197 nt
RNA3 NC_011807 353 nt

All sequences in the cluster

```
2 Asparagus virus 2 (NC_011807 OK058519)
1 Surrounding non-legume associated ilarvirus (MN412730)
1 Hydrangea vein-banding virus (OK666837)
```

Accession number / CP stop and 3'-adjacent 6 nt / CP-frame translation of sequence from immediately after CP stop codon to next in-frame stop codon (*), if present, or to end of available sequence

```
NC_011807 UGA CCACGC PRSGECVYLHSNECEKKNVIPFCHCECYDNACNTLWCPANVFCFHEP*
OK058519  UGA CCACGC PRSGECVYLHSNECEKKNVIPFCHCECYDNACNTLWCPANVFCFHEP*
MN412730  UGA CUACGC LRSDERKYLHSDECERKDVIPFCHCECYDYACNTMWCPANESCYHEPNG*
OK666837  UGA CUACGC LRSVERKYLHSDECERKDVIPFCHCECYDYACSTMWCPANESCYHEPNG*
```

CLUSTER 15

Alignment of 3'UTRs from the 3 segments of a representative genome

```
RNA3            UGACUAUCUGACAUGCCUCAAGUUAAUGAGUUGCCUUACAUAACAAACUGUUGUGUAAGA
RNA1            UAAUUAUUUGAAA-----------GAUGUUUCCUCUUUCUUGAUGCCCUCUUUUAGAGGA
RNA2            UAAUU-------------------AGUGAUGCCUCUUUUGAGAUGACC------------
                * * *                     **      ***     *    *

RNA3            UGCGGAUUUAAACCUCGACGAGUUGUAGAUCAACUAGUCUGGAGUUGUACCUUCUGUUUG
RNA1            U---GACACAACUUUAGGCACGUUA------------------------------CUUUG
RNA2            ---------ACAGUUUGGCGUCAUG------------------------------GUUCG
                         *    * * *    *                                ** *

RNA3            UAUAAACAGCGGGCUUAGUUUUCACGCUCUCUCGAUGUAAAUCUAAGAAUCCGUAUGUAA
RNA1            C-------------------------UUCUGGUAACGUUAGUUUG---------------
RNA2            C-------------------------UUCUGCC-AUGUU--UCUG---------------
                                           ***    * **   * *

RNA3            ACCUCCGAAUCGGACGAUUUCCACGUGCAACGUGAGGCCUUUGGGCCGGGUUUACAAUGG
RNA1            ----------------------------------------------UGGUUUUCCAGUAU
RNA2            ----------------------------------------------UGUGUUUCUAGUAA
                                                               *  ***  * *

RNA3            UUACCAAUAGUGUUUUCACACUAUUGAUGCCUCUUUUUAAAGAGAAG
RNA1            UAUCUAAUGAUAAU-----ACUGAUGCCUUCUAUAUAAAGAAGAUGC
RNA2            UAUCUAAUGAUAUU-----ACUAAUGCCUUC--UUUAAAGAAGAUGC
                *  * ***  *  *     ***  **    *  * *  * *
```

RNA1 NC_040393 152 nt
RNA2 NC_040392 121 nt
RNA3 NC_040394 287 nt

All sequences in the cluster

3 Cape gooseberry ilarvirus 1

Accession number / CP stop and 3'-adjacent 6 nt / CP-frame translation of sequence from immediately after CP stop codon to next in-frame stop codon (*), if present, or to end of available sequence

```
MZ357177  UGA CUAUCU LSDMPQVNELPYITNCCVRCGFKPRRVVDQLVWSCTFCLYKQRA*
MZ357180  UGA CUAUCU LSDMPQVNELPYITNCCVRCGFKPRRVVDQLVWSCTFCLYKQRA*
NC_040394 UGA CUAUCU LSDMPQVNELPYITNCCVRCGFKPRRVVDQLVWSCTFCLYKQRA*
```

CLUSTER 16

Alignment of 3'UTRs from the 3 segments of a representative genome

```
RNA3            UGACUACUUUCUGACCUCGACAAUGUCGAGUUGCCUCACAUGCGAUCUUCGUGUGUGAGA
RNA1            UGAC-------------------------------------GCGAGGCGCAUUUGCGCGU
RNA2            UGAU--------------------------------------------------------
                ***

RNA3            UGCGGAUUCUGUCCCAAAUAUGCUGUAGAUCAGCUUAUUUGGAGAUGUCAUUUGUGUGGA
RNA1            UCCGACGAUUUCGUCGAAGACGUACUAAACAUGGUUGUUUAGUG-------CUGUAUGUA
RNA2            ---------------GAGUUUGCAGCGGAU-------------GAUGGCGCUCGUACGUA
                                *    *      *              *         **  * *

RNA3            UAUCCACACAGUGCAUAGUUGCCUCGCAUCCGGGCGUGAAUCUAUGGUUUUCGUUUGAAC
RNA1            AUGUGAUUUGG--------------GCAUCCGUACGUUUCUCUAACGGACUUCAC-----
RNA2            UCUCUACACGA--------------GUUUCCGCG--------------------------
                     *                   *  ****

RNA3            GGUUUUCCACUUGCAAAGUGGGGCCUUUAGGCCAACGUUCAUAAAUGCCGUUAAUAAUAU
RNA1            -AUUUUCCAUU----------------------AAUAUCU---AAUGAUAUUAAUGAUGC
RNA2            -UUAUGCCACU----------------------AAUAUCU---AAUGAUAUUAGUGAUGC
                  * * *** *                      **  *     ****   *** * **

RNA3            AUAUAUAUAUUAUUAACGAUGCCUCCAAAGGAGAUGC
RNA1            CUCUAUAUAU-----------------AUAGAGAAGC
RNA2            CUCCUUA--------------------AUGGAGAUGC
                 *   **                    *  **** **
```

RNA1 OL584348 171 nt
RNA2 OL584349 107 nt
RNA3 OL584350 277 nt

All sequences in the cluster

3 Prunus virus I

Accession number / CP stop and 3'-adjacent 6 nt / CP-frame translation of sequence from immediately after CP stop codon to next in-frame stop codon (*), if present, or to end of available sequence

```
OL472065  UGA CUACUU LLSDLDDVELPHMRSSCVRCGFCPKYAVDQLIWRCHLCEYPHSA*
OL584350  UGA CUACUU LLSDLDNVELPHMRSSCVRCGFCPKYAVDQLIWRCHLCGYPHSA*
MW579755  UGA CUACUU LLTDLDNVELPHMRLSCVRCGFSPKYAVDQCIWRCHLCGYPHSA*
```

CLUSTER 17a

Alignment of 3'UTRs from the 3 segments of a representative genome

```
RNA1            ------------------------------------------------------------
RNA3            UGACUACACUCGGGCUUUUGUCCCGUGUGUGGUUAUGUACCACUUUGUCAUUGUAUGUGC
RNA2            ------------------------------------------------------------


RNA1            ----------------------------------------------------UAGAUCAA
RNA3            UACAAUUACUCAUGUGAUGUCAUUGGUUGUGAUGCCAAUGAUACCUUCUGUAUAGAA-GA
RNA2            ----------------------------------------------------UAAUU-UA
                                                                    **     *

RNA1            UGUUUUCGUGUAACAUGCAUGUUACCUAGUGAAGGGUCCUCCGGGAUGUGCUCAGCACCU
RNA3            UGAUCAAGUGUAACAUUCAUGUUACCUAGCGAAGGGUCCUCCGGGAUGUGCUCAGCACUC
RNA2            UUAACAAGUGUAACUUUCAUGUUACCUAGUGAAGGGUCCUCCGGGAUGUGCUCAGCACCU
                *      ******* * ************ ****************************

RNA1            AGCCUUAGCCAAGUUCAUAUGCCCACCUUUGCUUGUCUCCGGGUGGAUGCCUCAUGGUGC
RNA3            AGCCUUAGCUAAGUUCAUAUGCCCACCUUUGC-UGUCUCCGGGUGGAUGCCUCAUGGUGC
RNA2            AGCCUAAGCCAAGUUCAUAUGCCCACCUUUGCUUGUCUCCGGGUGGAUGCCUCAUGGUGC
                ***** *** ********************** ***************************

RNA1            UAUGAAUGCCUAUAAUUGAAAUAUUAUAGAUGCCUAAUUUUCCUCUCUUGAGGAAAAUUA
RNA3            UAUGGAUGC---------------------------------------------------
RNA2            UAUGGAUGCCUAUAACUGAAAUGUUAUAGAUGCCUAAUUUCUCUCUCUUGAGAGAAAUUA
                **** ****

RNA1            GAUGCCUCC-AAGGGAGAAGC
RNA3            ---------------------
RNA2            GAUGCCUCCAAAGGGAGAAGC
```

RNA1 NC_003833 208 nt
RNA2 NC_003834 208 nt
RNA3 NC_003835 247 nt (note, RNA3 sequence is 3'-truncated)

All sequences in the cluster

1 Tulare apple mosaic virus (NC_003835)
1 Rosa ilarvirus-1 (MT017863)

Accession number / CP stop and 3'-adjacent 6 nt / CP-frame translation of sequence from immediately after CP stop codon to next in-frame stop codon (*), if present, or to end of available sequence

```
MT017863  UGA CUACAC LHSGYCSSCGYVPYCHCQCFDGTCDVFGCVANDTVFYDEV*
NC_003835 UGA CUACAC LHSGFCPVCGYVPLCHCMCYNYSCDVIGCDANDTFCIEDDQV*
```

CLUSTER 17b

Alignment of 3'UTRs from the 3 segments of a representative genome

```
RNA1                U-----------------------------------------------------------
RNA2                UGA---------------------------------------------------------
RNA3                UGACCACGCUCGGGCUUAUGCCAGAGUUGUGGAUUUAUUCCAUAUUGUCAUUGUUAUUGU
                    *

RNA1                -------------------------------------------------------AAGUG
RNA2                -------------------------------------------------------AAGUG
RNA3                UGCGAUGACGUAUGUGAUGUCAUUGGAUGUUUAGCCAAUGAUACCGUCUAUGACGAAGUG
                                                                           *****

RNA1                UAAC-UUUCAUGUUACCUAGUGAAGGGUCCUCCGGGAUGUGCUCAGCACCUAGCCUAAGC
RNA2                UAACAUUUCAUGUUACCUAGUGAAGGAUCCUCCGGGAUGUGCUCAGCACCUAGCCUAAGC
RNA3                UAAC-AUUCAUGUUACCUAGUGAAGGGUCCUCCGGGAUGUGCUCAGCACCUAGCCUAAGC
                    ****  ******************** *********************************

RNA1                UAAGUCCAUAUGCCCACCUUUGCUUACUCCGGGUGGAAGCCUAUACAAAUGCUAUGGAUG
RNA2                UAAGUCCAUAUGCCCACCUUUGC-UACUCCGGGUGGAAGCCUAUACAAAUGCUAUGGAUG
RNA3                UAAGUCCAUAUGCCCACCUUUGC-UACUCCGGGUGGAAGCCUAUACAAAUGCUAUGGAUG
                    *********************** ************************************

RNA1                CCUAUAUUUGAAAUAAUAUAGAUGCCUAAA-UUUUCUCUCAUUGAG-AAAAUUUAGAUGC
RNA2                CCUAUAUUUGAAAUAAUAUAGAUGCCUAAA-UUUUCUCUCAUGGAG-AAAAUUUAGAUGC
RNA3                CCUAUAUUUGAAAUAAUAUAGAUGCCUAAAUUUUUCUCUCAUGGAGAAAAAUUUAGAUGC
                    ****************************** *********** *** *************

RNA1                CUCCCACGGAGAUGCAAUCGAAUUC
RNA2                CUCCAAAGGAGAUGC----------
RNA3                CUCCAAAGGAGAUGC----------
                    **** * ********
```

RNA1 NC_039075 208 nt
RNA2 NC_039074 200 nt
RNA3 NC_039076 313 nt

All sequences in the cluster

1 Tomato necrotic streak virus

Accession number / CP stop and 3'-adjacent 6 nt / CP-frame translation of sequence from immediately after CP stop codon to next in-frame stop codon (*), if present, or to end of available sequence

NC_039076 UGA CCACGC PRSGLCQSCGFIPYCHCYCCDDVCDVIGCLANDTVYDEV*

CLUSTER 18

Alignment of 3'UTRs from the 3 segments of a representative genome

```
RNA1            ------------------------------------------------------------
RNA2            U-----------------------------------------------------------
RNA3            UGACCACAAGAUUGUGAAUCUUGUGGUUAUAUUCCAUACUGCCACUGUGAAUGUUGGCCG

RNA1            ------------------------------------------------------------
RNA2            ---------------------------------------------------------AGU
RNA3            UACAGAUGCUACACCAUUGGGUGUAAUGCUAAUGAUCUAUGGUUUAUAGAUUCUUAAAGU

RNA1            ----------UGAGCUGAUGAAGGGUCCUCCGGGAUGUGCUCAGCACUUAACCUAAGUCA
RNA2            CUCACUUUCGUGAGCUGAUGAAGGGCCCUCCGGGGUGUGCUCAGCACUUAACCUAAGUCA
RNA3            CUCACUUUCGUGAGCUGAUGAAGGGCCCUCCGGGGUGUGCUCAGCACUUAACCUAAGUCA
                          *************** ******** *************************

RNA1            AGUUCAUAUGCCCACCUUUGCUGCUCCGGGUGGAUGCUUCGUGCAAAUGCUAUGAAUGCC
RNA2            AGUUCAUAUGCCCACCUUUGCUGCUCCGGGUGGAUGCCUCGUGCAAAUGCUAUGAAUGCC
RNA3            AGUUCAUAUGCCCACCUUUGCUGCUCCGGGUGGAUGCUUCGUGCAAAUGCUAUGAAUGCC
                ************************************* **********************

RNA1            UACGUGUACAAACGUAGAUGCCUAUAUUUUCUCUCCUGAGAAAAUAUAGAUGCCUCCCAA
RNA2            UACGUGUAUAUGCGUAGAUGCCUAUAUUUUCUCUCUUGAGAAAAUAUAGAUGCCUCUAAA
RNA3            UACGUAUAUGUGCGUAGAUGCCUAUAUUUUCUCUCCUGAGAAAAUAUAGAUGCCUCCAAA
                ***** **    *********************** ********************  **

RNA1            GGAGAUGC
RNA2            GGAGAUGC
RNA3            GGAGAUGC
                ********
```

RNA1 NC_003548 178 nt
RNA2 NC_003547 192 nt
RNA3 NC_003546 308 nt

All sequences in the cluster

3 Citrus leaf rugose virus

Accession number / CP stop and 3'-adjacent 6 nt / CP-frame translation of sequence from immediately after CP stop codon to next in-frame stop codon (*), if present, or to end of available sequence

```
NC_003546 UGA CCACAA PQDCESCGYIPYCHCECWPYRCYTIGCNANDLWFIDS*
JX256249  UGA CCACAA PQECESCGYIPYCHCECWPYRCYTIGCNANDLWFIDS*
MZ330102  UGA CCACAA PQDCESCGYIPYCHCECWPYRCYTIGCNANDLWFIDS*
```

CLUSTER 19

Alignment of 3'UTRs from the 3 segments of a representative genome

```
RNA2            UAGUGACCCCGGUUUACUAGUCCAUUGGACUAAUGGAAAUCCC-AUUGGGUCGUUGACUU
RNA1            UGAUG--------UUACUAGUCGUUUAGACUAAUGAAAAUCCCAAUUGGGUCAUUGACUU
RNA3            ------------------------------UAAUGAAAAUCCU-AUUGGGUCGUUGACGU
                                              ***** ******   ******** ***** *

RNA2            UGUCAACGAGUUAACUACGAUGUCGGCCCUAGGACAUCUAUCGAGCUAACGACGAUCGCU
RNA1            UGUCAAUGAGUUAACUACGAUGUCGGCCCUAGGACAUCUAUCGAGUUAACGACGAUCGCU
RNA3            UGUCAACGAGUUAACUACGAUGUCGGCCCUAGGACAUCUAUCGAGUUAACGACGAUCGCU
                ****** ************************************** **************

RNA2            UUGCGAUUAUGCUUCAUUUUUCCUUUCGGGAAAAUGAAAUGACCCUUAGGGGCC
RNA1            UUGCGAUUAUGCUUCAUUUUUCCUUUCGGAAAAAAUGAAAUGACCCUUAGGGGCC
RNA3            UUGCGAUUAUGCUUCAUUUUUUCUUUCGAAAAAAAUGAAAUGACCCUUAGGGGCC
                ********************* ******  ************************
```

RNA1 NC_003451 166 nt
RNA2 NC_003452 173 nt
RNA3 NC_003453 143 nt

Note: No insert in RNA3, so no evidence for RT domain.

All sequences in the cluster

3 American plum line pattern virus

Accession number / CP stop and 3'-adjacent 6 nt / CP-frame translation of sequence from immediately after CP stop codon to next in-frame stop codon (*), if present, or to end of available sequence

```
KY883317  UAA UGAAAA *
NC_003453 UAA UGAAAA *
LC496471  UAA UGAAAA *
```

CLUSTER 20

Alignment of 3'UTRs from the 3 segments of a representative genome

```
RNA1            U-----------------------------------------------------------
RNA2            U-----------------------------------------------------------
RNA3            UGAAGGUCCCAAUCCUCACAUGAUGAGAUGCCGCACGUCAAGAACAAUUCUUGUGUGCGA
                *

RNA1            -------------------------------------AAUUGUGUUC-------------
RNA2            ------------------------------------------------------------
RNA3            UGUGGAUUCAGACCACCGCAGAUUAUAGAUCAGUAUGUAUGGCGUUGCCGUAGAUGCUAU


RNA1            -------------------------------------------------AGUUACUUUGU
RNA2            -------------------------------------------------AAUUUAUUGA-
RNA3            CUAGAACAUGCCGCCUGAACCAUUGUUUCAAGGUUUCGCACUUGAUACAAAUUUGUUUAA
                                                                 * **  **

RNA1            AACUAUGCCUCCUUUGGAGAUGACACGUU-CACAUGGCGCUCACUCGCAUCUUCGGUGAG
RNA2            ----AUGCC-------------AUUGAUUAUGAAGGCCACUCAGUCGCAUUUCAGCUGAG
RNA3            AUUUAUGCCUCCAUUGGAGAUGAUUGAUCAUGUAGGCCGUUUGGUUGCAUUCUUACCAAG
                    *****             *    *     * * *  *   * ****        **

RNA1            UG-UGUUCGUGAUUCUAUCAAUACCUAAUUGGUAUUGAUAAUGCCUCUAUUUCUAGAGAU
RNA2            UGAUGAUCAUGUUACCAUUAAUAUCUAA-AGAUAUUAAUGAUGCCUCUAUUUCUAGAGAU
RNA3            CGUUGAUCAUGUUGCCAUUAAUAUCUAA-UGAUAUUAAUGAUGCCUCUAUUUCUAGAGAU
                 * ** ** ** * * ** **** ****  * **** ** ********************

RNA1            GC
RNA2            GC
RNA3            GC
                **


RNA1 OM323989 143 nt
RNA2 OM323990 115 nt
RNA3 OM323991 301 nt
```

All sequences in the cluster

```
      3 Bacopa chlorosis virus
```

Accession number / CP stop and 3'-adjacent 6 nt / CP-frame translation of sequence from immediately after CP stop codon to next in-frame stop codon (*), if present, or to end of available sequence

```
JQ015298  UGA AGGUCC RSQSSHDEMPHVKNNSCVRCGFRPPQIIDQYVWRCRRCYLEHAA*
OM323991  UGA AGGUCC RSQSSHDEMPHVKNNSCVRCGFRPPQIIDQYVWRCRRCYLEHAA*
OM323992  UGA AGGUCC RSQSSHDEMPHVKNNSCVRCGFRPPQIIDQYVWRCRRCYLEHAA*
```

Note the unusual 3' context for RT, but RNA3 does contain an insert relative to RNAs 2 and 3, and the downstream peptide contains a Zn-finger domain, suggesting that RT does occur.

CLUSTER 21

Alignment of 3'UTRs from the 3 segments of a representative genome

```
RNA1            UAGAUCGGAUUUGGUACCGAUCGAAAUGACCGGUCCUCCGUGAGGGUUUACCGGUCGCAU
RNA2            UAGGUUGGGUUUGGUACCAACCGGAAUGAUCGGUCCUCCGUGAGGAUUUACCGAUCGCAU
RNA3            UAGGUUGGGUUUGGUACCAACCGAAAUGAUCGGUCCUCCGUGAGGAUUUACCGAUCGCAU
                *** * ** ********* * ** ***** *************** ******* ******

RNA1            UCGAGAGUUCGCCUCCUAGGAAUCGACCUAAGAAUGCAAGUUACCUAAUAAGGUAAUAUU
RNA2            UCGAGAGUUCGCCUCCUAGGACUCGACCUAAGAA--------------------------
RNA3            UCGAGAGUUCGCCUCCUAGGACUCGACCUAAGAAUGCAAGUUACCGAAUAAGGUAAUAUU
                ********************* ************

RNA1            CCAUGAAUCGUGAAGAUU--------------------
RNA2            --------------------------------------
RNA3            CCAUGAAUCGUGAAGAUUCAUGAUGCCCCGCAGAACAG
```

```
RNA1 MW962369 138 nt
RNA2 MW962372 94 nt
RNA3 MW962375 158 nt
```

Note: No insert in RNA3, so no evidence for RT domain.

All sequences in the cluster

```
      3 Green Sichuan pepper ilarvirus
```

Accession number / CP stop and 3'-adjacent 6 nt / CP-frame translation of sequence from immediately after CP stop codon to next in-frame stop codon (*), if present, or to end of available sequence

```
MW962377  UAG GUCGGG VGFGTDRIDRSSVRIYRLHSRVRLLGLDLRMQVIEQDDIP*
MW962376  UAG GUUGGG VGFGTNRNDRSSVRIYRSHSRVRLLGLDLRMQVTE*
MW962375  UAG GUUGGG VGFGTNRNDRSSVRIYRSHSRVRLLGLDLRMQVTE*
```

CLUSTER 22

Alignment of 3'UTRs from the 3 segments of a representative genome

```
RNA1            UAGUGACUCUACAUGAGUCCUAGUGAAGGAUCCUCCGGGAUGUGCUCAGCACCUAGCCUA
RNA3            ---UGACUCUACAUGAGUCCUAGUGAAGGAUCCUCCGGGAUGUGCUCAGCACCUAGCCUA
RNA2            -------------UGAGUCCUAGUGAAGGAUCCUCCGGGAUGUGCUCAGCACCUAGCCUA
                             ***********************************************

RNA1            AGCUAAGUUCAUAUGCCCACCUAUGCUGCUCCGGGUGGAUGCUUAUUUGUUUAUGAAUGC
RNA3            AGCUAAGUUCAUAUGCCCACCUAUGCUGCUCCGGGUGGAUGUUUAUUUGUUUAUGAAUGC
RNA2            AGCUAAGUUCAUAUGCCCACCUAUGCUGCUCCGGGUGGAUGUUUAUUUGUUUAUGAAUGC
                ***************************************** ******************

RNA1            CUAUGAUUGAAAUAUCAUAGAUGCCUAAAUUUUUCUCUCUUGAGAAAAAUUUAGAUGCCU
RNA3            CUAUGAUUGAAAUAUCAUAGAUGCCUAAAUUUUUCUCUCUUGAGAAAAAUUUAGAUGCCU
RNA2            CUAUGAUUGAAAUAUCAUAGAUGCCUAAAUUUUUCUCUCUUGAGAAAAAUUUAGAUGCCU
                ************************************************************

RNA1            CCUAGGAGACGC
RNA3            CCAAGGAGACGC
RNA2            CCUAGGAGACGC
                ** *********
```

RNA1 ON932434 192 nt
RNA2 ON932435 179 nt
RNA3 ON932436 189 nt

Note: No insert in RNA3, so no evidence for RT domain.

All sequences in the cluster

```
      1 Blacklegged tick associated ilarvirus (MG647776)
      1 Apple ilarvirus 2 (ON932436)
```

Accession number / CP stop and 3'-adjacent 6 nt / CP-frame translation of sequence from immediately after CP stop codon to next in-frame stop codon (*), if present, or to end of available sequence

```
MG647776  UGA CUCUAC LYMSPSEGSSGMCSAPSLS*
ON932436  UGA CUCUAC LYMSPSEGSSGMCSAPSLS*
```

Note the somewhat leaky stop codon context (UGA CU), but no insert in RNA3 relative to RNAs 1 and 2, and no Zn-finger in the downstream peptide.

CLUSTER 23

Alignment of 3'UTRs from the 3 segments of a representative genome

```
RNA1                UAGUU-------------------------------------------------------
RNA2                UGAG--------------------------------------------------------
RNA3                UGACUAUAUGAUCCAUUCUUUGAUUGUUCCGACAGUGCUAACUUUUCAUUUCUUCCACCG
                    *

RNA1                --------------------------------------------------UUGUGUCCAG
RNA2                --------------------------------------------------CUUUACCUAG
RNA3                GAAGAAAUGGAAUUUAAAGCCUGGCGAUGCUGUUAUUGCAAUGAAAUUCAUUAUGUCUAG
                                                                   * *  * **

RNA1                AUUUUGUCUCUGGAUGGUUUAAAUCCUUUUGGUUAACUUUGUACUAAGUACAUGAAAGUU
RNA2                AUUUUGUCUCUAGAUGA-AUAAAUCCUUUUGGUUAACUUCGUACUUUGUACAUGGAAGUU
RNA3                AUUUUGUCUCUAGAUGA-AUAAAUCCUUUUGGUUAACUUCGUACUUUGUACAUGGAAGUU
                    *********** ****   ******************** *****  ******* *****

RNA1                AAGAUAAGAAUGCCUACUGUUUCCUAAGUGGA----------------------------
RNA2                AAGAUAAAAAUGCCUACUGUUUCCUAAGUGGAUGACACUUUUUAUUACCUACAUUUGUAG
RNA3                AAGAUAAAAAUGCCUACUGUUUCCUAAGUGGAUGACACUUUUUAUUGCCUACAUUUGUAG
                    ******* ************************

RNA1                ----------------------------------------
RNA2                AUGCCCUUACCGUGAGGUAAGGAUGCCCCUUUUGGGAAGC
RNA3                AUGCCCUUACCGUGAGGUAAGGAUGCCCCUUUUGGGAAGC
```

RNA1 KX196166 107 nt
RNA2 KX196165 173 nt
RNA3 KX196164 279 nt

All sequences in the cluster

2 Viola white distortion associated virus

Accession number / CP stop and 3'-adjacent 6 nt / CP-frame translation of sequence from immediately after CP stop codon to next in-frame stop codon (*), if present, or to end of available sequence

```
GU168941  UGA CUAUAU LYDPFFDCSDSANISFLPPEEMEFKAWRCCYCNEIHYV*
KX196164  UGA CUAUAU LYDPFFDCSDSANFSFLPPEEMEFKAWRCCYCNEIHYV*
```

CLUSTER 24

Alignment of 3'UTRs from the 3 segments of a representative genome

```
RNA3            UAAAUCUAGCAUUGUUAUUCCAACCAUCUUGCUGGUUGAUGAAUAGUCCGGUCUUGCACC
RNA2            UGACUUGUGUC-UCUUGUCCCGGCU-----UUUAGCCGAUGACUGGUCCGGUUUUGCACC
RNA1            UGAAUUAAAUCAGCAUGUCCCAACCA----AUAGGUUGAUGACUAGUCCGGUCUUGCACC
                * * *          * * **  *          *  ***** * ******* *******

RNA3            CGGAUGAGAUUAAAUCCAUAUGGUUCGACUCUCUCAGAGGUUUAAGUCGAAGACAUAUAU
RNA2            CGGAUGAGAUUAAAUCCAUAUGGUUCGACUCUCUCAGAGGUUUAAGUCGAAGACAUCUAU
RNA1            CGGAUGAGAUUAAAUCCAUAUGGUUCGACUCUCUCAGAGGUUUAAGUCGAAGACAUCUAU
                ******************************************************** ***

RNA3            GCCUAUUGCCCCCUGUAGGAGUGCACUAGAUGUUGCGUGAAGAUAUUUCUUCAUUUGCCC
RNA2            GCCUAUUGCCCCCUGUAGGAGUGCACUAGAUGUUGCGUGAAGAUAUUUCUUCAUUUGCCC
RNA1            GCCUAUUGCCCCCUGUAGGAGUGCAAUAGAUGUUGCGUGAAGAUAUUUCUUCAUUUGCCC
                ************************* **********************************

RNA3            UACCAAAAGGUAGGAUGCCCCUUUAAGGGAGGC
RNA2            UACCAAAAGGUAGGAUGCCCCUUUAAGGGAGGC
RNA1            UACCAAAAGGUAGGAUGCCCCUUUAAGGGAGGC
                *********************************
```

```
RNA1 NC_006064 209 nt
RNA2 NC_006065 207 nt
RNA3 NC_006066 213 nt
```

Note: No insert in RNA3, so no evidence for RT domain.

All sequences in the cluster

```
      1 Humulus japonicus latent virus
```

Accession number / CP stop and 3'-adjacent 6 nt / CP-frame translation of sequence from immediately after CP stop codon to next in-frame stop codon (*), if present, or to end of available sequence

```
NC_006066 UAA AUCUAG I*
```

CLUSTER 25

Alignment of 3'UTRs from the 3 segments of a representative genome

```
RNA3            UGAAGGUCAACCUCCUCAGGGAGUGAGUUGCCGUGCAUUAUGAAUAAUUCAUGUGCACGA
RNA1            ------------------------------------------------------------
RNA2            ------------------------------------------------------------


RNA3            UGCGGUUUUAGGCCUCCGUAUGUUAUUGAUCAAUACAUAUGGUCGUGUGCUUUAUGCCAU
RNA1            -----------------------------UAAUUAUGUUUAGCUUUUGGCUAUUUUCCGU
RNA2            -----------------------------UAA-----UUUAGCUGUUG-----UUUCCGU
                                             * *     * * *   *        * ** *

RNA3            UAUAAGCAUUAUGCCUAAACCAAGAAGCAAAUUUCUUGCUUUAUGUACCAAAUUAUUUGA
RNA1            U---------------------------------------------------UUA--CGA
RNA2            U---------------------------------------------------UUAUACGA
                *                                                   ***   **

RNA3            UGCCUCGCUUACGCGAGAAGUUCCAAUCAAU-AGGCGAUUUAGUUGCAUUCUGACUAAAU
RNA1            UGCCUCGAUUUAUCGAGAAGAAUCAAUCAGGUUGGCACUUUAGACGCAUUCUAUCUAAAG
RNA2            UGCCUCGGUUUACCGAGAAGAGUCAAUCAUUAAGGCGCUUUAGACGCAUUCUAUCUAAAG
                ******* **   *******   ******    ***  *****  *******  *****

RNA3            UUGAUUUGGAAAGUUUCCACUGAUAUCUAAUGAUAUCAGUGAUGCCUCUAUUUCUAGAGA
RNA1            CUGAUU----GAUUUUCCAUUAAUAUCUAAUGAUAUUAAUGAUGCCUCCAUU--UGGAGA
RNA2            UUGAUU----GAUUUUCCAUUAAUAUCUAAUGAUAUUAAUGAUGCCUCCAUU--UGGAGA
                 *****     * ****** * ************** * ********* ***  * ****

RNA3            UGC
RNA1            UGC
RNA2            UGC
                ***
```

RNA1 OL539723 155 nt
RNA2 OL539724 147 nt
RNA3 OL539725 302 nt

All sequences in the cluster

1 Soybean ilarvirus I

Accession number / CP stop and 3'-adjacent 6 nt / CP-frame translation of sequence from immediately after CP stop codon to next in-frame stop codon (*), if present, or to end of available sequence

OL539725 UGA AGGUCA RSTSSGSELPCIMNNSCARCGFRPPYVIDQYIWSCALCHYKHYA*

Note the unusual 3' context for RT, but RNA3 does contain an insert relative to RNAs 2 and 3, and the downstream peptide contains a Zn-finger domain, suggesting that RT does occur.

CLUSTER 26

Unable to generate alignment of 3'UTRs from RNAs 1, 2 and 3 since the only available sequences are MN386958.1 (RNA3) and MN386957.1 (partial RNA2 without 3'UTR).

All sequences in the cluster

1 Apple ilarvirus 1

Accession number / CP stop and 3'-adjacent 6 nt / CP-frame translation of sequence from immediately after CP stop codon to next in-frame stop codon (*), if present, or to end of available sequence

MN386958 UGA AUCGCG IAEFRPCDDARTGAECSDDESDLDSTVVHDYSDDDFYYSDVYQFDDDRI*

CLUSTER 27

Alignment of 3'UTRs from the 3 segments of a representative genome

```
RNA3            UGACUACACUCGGGUGGAGUAAAUUUCGAUACCUGGAACGGAAUUUGUUCCAGAUGCGGG
RNA1            -UAAUACUUUCGGAAGUA----------AUUCCCGAAAUGAAAAUC-UUCCA--------
RNA2            --------------------------------------UGAAAAUC-UUCCA--------
                                                       * ** *  *****

RNA3            UUUAAACCCAAGCGGUUAGUAGAUCAAUAUACAUACACUUGCCGUGAAUGCGGAAAGAUA
RNA1            -----------------------------------------CUGAAUAUUCAGUGAAGU-
RNA2            -----------------------------------------CUGAAAAUUCAGUGAAGU-
                                                         * *   ** * *  *  *

RNA3            UACUUCGCAUAAAUCUUGAUAAUUCAAGUUAAGUCUCACUUAGUGAAAUAAGACUUAAGA
RNA1            ----------------------------UUGGGUCUCAUGGAG---AUUUAACCCAAAGA
RNA2            ----------------------------UUGGGUCUCAUGGAG---AUUUAACCCAAAGA
                                            **  ******   **   * * *  *  ****

RNA3            CACAUAUCCCAUCUAUCGCUCCCUGAGAUGAUUGAUUGUGUUGCCUAAAGAACGUUAUUA
RNA1            AACUUAUGCCUUUCGCCAUAACCGAAG-------------------GAUGAAUUUUAAGU
RNA2            AACUCAUGCCUUUCGCCAC-ACCGAAG-------------------GAUGAAUUCUGAGU
                 **  ** ** *    *    **  **                    * ***   *

RNA3            CUUGACUGGUAACGAUUUUCUAUAGAUGCUCACUGUUUAUUAUAAAACAGU--GAAUGCC
RNA1            UUCGAGUGAU--CGA------AUAUACGAUCAUUUUCCCUAAUCAAACGAUUAGGAUGCC
RNA2            UUCAAGUGAU--CGA------AUAUACGAUCAUUUUCCCUAAUCAAAUGAUUAGGAUGCC
                 *  * ** *  ***      *** * * *** * *   * ** ***   *  * *****

RNA3            CCCAUUGGGA-AGC
RNA1            ACCAAGGUGAUGGC
RNA2            ACCAAGGUGAUGGC
                 ***  * **  **
```

RNA1 MZ170696 194 nt
RNA2 MZ170697 166 nt
RNA3 MZ170698 311 nt

All sequences in the cluster

1 Water chestnut virus A

Accession number / CP stop and 3'-adjacent 6 nt / CP-frame translation of sequence from immediately after CP stop codon to next in-frame stop codon (*), if present, or to end of available sequence

MZ170698 UGA CUACAC LHSGGVNFDTWNGICSRCGFKPKRLVDQYTYTCRECGKIYFA*

CLUSTER 28

Alignment of 3'UTRs from the 3 segments of a representative genome

```
RNA3            UGAGAACCGAAUGGUUCUCGGUUGUCCGAUUAGGACAUUCCCCAUUUUGGGGAUAGAAGA
RNA1            UGAUUA--GGAUCAUUUG-----AUCCGAAUUUGA----CUCGAUUUAGAGUCUUGAAAC
RNA2            UGAGUCCC-----CUUUGUUGCUCUUUGAGCAUGA----CUCGAUUUAGAGUCUUGAAGC
                ***           **        *  **    **    * * **** * *  * ***

RNA3            GAUUGAUCACUCUUCG-AAUUUCCACGAAU-GUGAAGUGCCGUGCCCCUUGGGACAAAAC
RNA1            GAUUGAUCACGUUUCGAAAUUUCCACGUUUAGUGAAGUACCGUGCCCCUUGGGAACAAAC
RNA2            GAUUGAUCACGCUUCGAAAUUUCCACGUUUAGUGAAGUACCGUGCCCCUUGGGAACAAAC
                **********  **** **********  * ******* ***************  ****

RNA3            GGCAGCGAAAGAUAGGUUUGCGUUCCCAAACCGGCGAUCAC-UUUUGCAACAAAUUCGAA
RNA1            GGUAGUGAAAUGAAGGUUUGCGUUCCCAAACCGACGAUCACAUUUUGCAACAAAUUCGAU
RNA2            GGUAGCGAGAUGAAGGUUUGCGUUCCCAAACCGACGAUCACAUUUUGCAACAAAUUCGAU
                ** ** ** *   ******************** ******* *****************

RNA3            UGAAUUUGAUUCCCACACCCGUGAAGGGUAUGGAUGCUCCGUUAGGAUCCCGGGAAUUCG
RNA1            AGAAUUUGAUUCCCACACCCGAGAAGGAUAUAGAUGCCCUGUGAGGCUAACA--------
RNA2            AGAAUUUGAUUCCCACACCCGUGAAGGGUAUAGAUGCUCCGUUAGGAACGGA-----UCC
                 ******************** ***** *** ***** * ** ***

RNA3            G
RNA1            -
RNA2            C
```

RNA1 KU947034 221 nt
RNA2 KU947033 227 nt
RNA3 KU947035 238 nt

Note: No insert in RNA3, so no evidence for RT domain.

All sequences in the cluster

1 Lilac leaf chlorosis virus

Accession number / CP stop and 3'-adjacent 6 nt / CP-frame translation of sequence from immediately after CP stop codon to next in-frame stop codon (*), if present, or to end of available sequence

KU947035 UGA GAACCG EPNGSRLSD*

Note that lilac leaf chlorosis virus also occurs as single-sequence cluster 31. This is because the two sequences, NC_025481 and KU947035, have only 70.6% amino acid identity to each other so were split into separate clusters as a result of the 80% amino acid identity used in BLASTCLUST.

CLUSTER 29

Alignment of 3'UTRs from the 3 segments of a representative genome

```
RNA3            UGACUAGAUGGUCUAGUCACUUCCGGACGUGUGUCCGAUUUGCCUCACAUAGAUGGAAAU
RNA1            --------UGAACUAGUAUUUAGGCAUCGUA-GUCGUAUCUUUUCUACGUUUAC------
RNA2            --------UAGAUUGGCAGCU---GAACGCA-UUCUAUUCAGUCCUA--UUGAU------
                        *    * *    *      **    **   *       *  *  *

RNA3            UCCUGUGUGAGAUGUGGAUAUGCUCCCCGUUACAUUGUAGAUCAAUGUUAUUGGGAAUGC
RNA1            --------------------UAGUUUCCAUCAC-----UGACUAAUGUCGGUGAUGAUGC
RNA2            ----------------------UUUCCCGUUAA-----UAUCUAAUGAUAUUAACGAUGC
                                       *  ** * *            ****    *     ****

RNA3            ACUCUUUGUUCGUAUGAACAACGGGCGUAGCAACCUCGCAUCCGGGUUUAAGACUACGUA
RNA1            ------------------------------------------------------------
RNA2            ------------------------------------------------------------


RNA3            UUCUACUAUUAUUAAUUUCUUAAUAAUAGUAUUGCCUCCAAAGGAGAUGC
RNA1            ----------------CUCUUUAAAAU-------------AAAGAGAUGC
RNA2            ----------------CUCCC----AU-------------AAAGAGAUGC
                                 **      **              ** *******
```

RNA1 NC_027928 101 nt
RNA2 NC_027929 90 nt
RNA3 NC_027930 230 nt

All sequences in the cluster

1 Privet ringspot virus

Accession number / CP stop and 3'-adjacent 6 nt / CP-frame translation of sequence from immediately after CP stop codon to next in-frame stop codon (*), if present, or to end of available sequence

NC_027930 UGA CUAGAU LDGLVTSGRVSDLPHIDGNSCVRCGYAPRYIVDQCYWECTLCSYEQRA*

CLUSTER 30

Alignment of 3'UTRs from the 3 segments of a representative genome

```
RNA3            UGACUCACUGCUGAUUCUUCAUCAACUGUUGUUGAUGAAUUGCCUCACAUGGAUGGUAAU
RNA1            --------UAGAGAGUUUCUUUCCACU-UUAGUGAUG-----------------------
RNA2            --------------------------------UGAUG-----------------------
                                                *****

RNA3            UCCUGUGUGAGAUGCGGAUUUACACCGAAACGUAUUGUAGAUCAAUACGUAUGGCGAUGU
RNA1            CCCCUUAUGGGAAGACGG-------------------------------UAUUGUUACCG
RNA2            CCUCUUAUGAGAUGAG-----------------------------------------UCU
                 *   * ** ** *

RNA3            UACCUUUGUGAUUAUCCACAAAUGGCUUAGUUUUCCCGCUCUCGAUAUAAAUCUAAGAGU
RNA1            UAAAUACGUGAUGG----------------------CGCACUCGACGCUUCUUACCGAGU
RNA2            UAGAUGUGGGCU--------------------CUCCCGCGCUU--------CUUGGGAGU
                **  *  * * *                        *** **              ****

RNA3            CCGCAGUAAACUUCCGAAUCGGACGAUUCCACGUGCAACGUGAGACCUUUGGGUCGAGUU
RNA1            GUCACGUAUUAUUUCCAA--------------------------------------AGAA
RNA2            AUUCUAAGAUUUUCCGAU--------------------------------------AAUA
                           ** * *                                       *

RNA3            UACAAGGGUUUUCCAAUGGUGUAUAAACACCAUUGAUGCC-UCUAUUAAG-AGAAGC
RNA1            AACUAAGUUUU-------------------CUUUGAUGCCUUCUAUAAAGAAGAAGC
RNA2            UAUAAUUAUAU-------------------UAUCGAUGCCUUCUAUAAAGAAGAAGC
                 *  *   * *                     * ****** ***** *** ******
```

RNA1 NC_022127 156 nt
RNA2 NC_022128 116 nt
RNA3 NC_022129 295 nt

All sequences in the cluster

1 Ageratum latent virus 1998

Accession number / CP stop and 3'-adjacent 6 nt / CP-frame translation of sequence from immediately after CP stop codon to next in-frame stop codon (*), if present, or to end of available sequence

NC_022129 UGA CUCACU LTADSSSTVVDELPHMDGNSCVRCGFTPKRIVDQYVWRCYLCDYPQMA*

CLUSTER 31

Alignment of 3'UTRs from the 3 segments of a representative genome

```
RNA3            -----------------UAGGUAUGAAUUGACCUCAUACCGAAGUACCGCCGAAU-GGCG
RNA1            UGA----UUUGAAGUGCUGGGUUUUAGUUGACCAGAAAACCGAAUACCACCGAAUAGGUG
RNA2            UGAAAACUUGUGUUUUCUAGGACCGAUUUGAUCACGGUCCGA--UACCACCGAUUAGGUG
                                 * **    * **** *      *    **** **** * ** *

RNA3            AUGGACCC-UUCCCCGAAUGGGAAGAAAGGGUCGUACAACUUAGAAAGAGCGUUCCCUCU
RNA1            AUGUACCCUUUCCCCGUUAGGGAGGUGUGGGUAGUGUAACAAAGGAAAUGCGUUCCCAUU
RNA2            AUGUACCCUUUCCCCGUUAGGGAGGUGUGGGUAGUGUGACAAAGGAAAUGCGUUCCCAUU
                *** **** *******   **** *   **** **   **  ** **  ********  *

RNA3            UUCGAAGGUCAAGUUGUGCAACAGAGACGAAUAAGAUCUCUGAUUCCUAUAUCCGUGAAG
RNA1            UCCGAUGGUCUUGUUAUGCAACA-ACACGAAUGUG----UUGAUUCCUAUAUCCGAGAAG
RNA2            UCCGAUGAUCUUGUCUCGCAACA-ACACGAAAGUG----UUGAUUCCUAUAUCCGAGAAG
                * *** * **  **   ****** * *****   *     *************** ****

RNA3            GAUAUAGAUGCUCCGUUAGGAUCCCGGGAAUUCGG
RNA1            GAUAUAGAUGC-CCUUUAGGAACC-----------
RNA2            GAUAUAGAUGCUCCGUUAGGAAC---GGAUCCC--
                *********** ** ****** *
```

RNA1 NC_025477 194 nt
RNA2 NC_025478 203 nt
RNA3 NC_025481 196 nt

Note: No insert in RNA3, so no evidence for RT domain.

All sequences in the cluster

      1 Lilac leaf chlorosis virus

Accession number / CP stop and 3'-adjacent 6 nt / CP-frame translation of sequence from immediately after CP stop codon to next in-frame stop codon (*), if present, or to end of available sequence

NC_025481 UAG GUAUGA V*

Note that lilac leaf chlorosis virus also occurs as single-sequence cluster 28. This is because the two sequences, NC_025481 and KU947035, have only 70.6% amino acid identity to each other so were split into separate clusters as a result of the 80% amino acid identity used in BLASTCLUST.

CLUSTER 32

Unable to generate alignment of 3'UTRs from RNAs 1, 2 and 3 since the only available sequences are JN107637.1 (RNA1, partial sequence without 3'UTR), JN107638.1 (RNA2, partial sequence without 3'UTR) and JN107639.1 (RNA3).

All sequences in the cluster

```
      1 Raphanus latent virus
```

Accession number / CP stop and 3'-adjacent 6 nt / CP-frame translation of sequence from immediately after CP stop codon to next in-frame stop codon (*), if present, or to end of available sequence

```
JN107639  UGA CUAUUU LFSQLSNLVDATYSDDSVSDPEN
```

CLUSTER 33

Alignment of 3'UTRs from the 3 segments of a representative genome

```
RNA3            UGACCACGCUCGGGGUGUGAAUCGUGUGGUUAUGUACCAUAUUGCCAUUGUAAAUGCUAC
RNA1            -----------------------------UAAUUUGCAAUGUU-------UAAACAUUUC
RNA2            --------------------------------------------------UAAACG----
                                                                  ****

RNA3            AAUGAUGCCUGCUCAGUGGUUGGUUGUGAGCAGAAUAUCUGGUUUAUACCAGAUGAAGUG
RNA1            ----------------------------------------------------AUGGAACG
RNA2            ------------------------------------------------------------


RNA3            UUUCCAUAGGAACCUUAUCAGACGUCUUCAGGGAUGUACUAAGUACCUCGGUCUGAUGAA
RNA1            ACUAAAUG-----------------CCUCUUGGGUGUACUUAGUA-CUUAAUUUAGUCGA
RNA2            -------------------------CUCCCGGAGUGUACUUAGUA-CGAAGUUUAAACAA
                                         *  *  *  ****** **** *    * *     *

RNA3            AUCUAUAUGCCUAGUCGCCUCUACAACUAGA-GUCCUCUAUAGAUGCCCCAUACUGUAAU
RNA1            AUCUAUAUGCCCAGCCGCCUCUACAGCUGGAUGACCUCUAUAGAUGCCUCUUAUAUUAUU
RNA2            AUCUAUAUGCCCAGCCGCCUCUACAGCUGGAUGUCCUCUAUAGAUGCCUCUUGUGUUAAC
                *********** ** ********** ** ** * ************** * *    **

RNA3            GUUUGGGAUGCCAAAUCUCUCUCUCUUGAGAGAGAUUUGAUGCCUACCUUUGGUAGAGGC
RNA1            AUGAGAGAUGCCAAGUUUC-CUUUCCGAAGAGAAACUUGAUGCCUACCUACGGUAGAGGC
RNA2            AUAAGAGAUGCCAAGUUUC-CUUUCCGAAGAGAAACUUGAUGCCUACCUACGGUAGAGGC
                 *  * ******** * ** ** **   ***** * *************  *********

RNA3            GAC--
RNA1            -----
RNA2            GGCUG
```

RNA1 OL472057 193 nt
RNA2 OL472058 164 nt
RNA3 OL472059 302 nt

All sequences in the cluster

1 Tomato ilarvirus 1

Accession number / CP stop and 3'-adjacent 6 nt / CP-frame translation of sequence from immediately after CP stop codon to next in-frame stop codon (*), if present, or to end of available sequence

OL472059 UGA CCACGC PRSGCESCGYVPYCHCKCYNDACSVVGCEQNIWFIPDEVFP*

CLUSTER 34

Alignment of 3'UTRs from the 3 segments of a representative genome

```
RNA2            UAGU-------------CUGAU---------------UCUCCGGAAAUGCUCAGCAGU--
RNA1            UAAC-------------UCAAU-------------AGUCCCAGGACAGUCUCAGACGU--
RNA3            UGACCACACUCGGGGGGUCGAUGUUUGGUUUGCGAAGUCUCUUGGGGCUUGCAAACGUAU
                *                   **               ** *  *       **   **

RNA2            -------------------------------------------------------UCGG-
RNA1            -----------------------------------------------------UGUUGA-
RNA3            ACUCGAAUCGACGACUUUAGUCUUCGAGACGAAUGCCUGUAUUGUGGAGCAGAUGCUGAA
                                                                         *

RNA2            --------AUUA------------------------------------------------
RNA1            --------GUUAUU----------------------------------------------
RNA3            AUCGGACUGUUAUGUCCGUCUUUCAAAUGCCCUCAGGGGUGCACUCAGUGCCUUUGAUUU
                         ***

RNA2            --ACGAGUUUAUGUUCCAAUCGUUUGUUUCUCCCGAUUGAUGCCUAUUGGUGUUCAUAAA
RNA1            -------UCUAUAUACCCACUGUAUGUUUCUCCCAGUGGAUGCCUAUUUAAGGUGUUUAU
RNA3            GGAUGAGUUUAUAUUCCGUUCGUAUGUUUCUCCCGAACGAUGCCUA--UAUGGUGUUUAU
                       * *** * **    ** **********    ********     * *  * *

RNA2            AAAAAAAAAUGUAC-AAUGUCAUGUG---CUACGCUUAUUUACGUGAGCU----UCAUCA
RNA1            AGAAGAUUAUGUGCAAGUCACAUAAGUUCCUUAUCUCUCUCUCAGGAGAGGAGAUAAG--
RNA3            AGACGUGCAUGCAUUUAU-GUAUGUGUACCUAAUCUCUCUCUCAGGAGAGGAGAUUAGAA
                * *     ***      *   **  *   **   **   *  *  ***      * *

RNA2            CUUGAAUCAGUAUCAAUCGUAGUGAAUGUGAAUAUACACC--------------------
RNA1            ---------------------------------AUGCCUCUAAGGAG-------------
RNA3            AAAAAAAAAGCAGAGAAAGUGUUAGAUGGAGUAAUAUAACAAAGAAGAAUAACUAUUGUA
                                                 **   *

RNA2            AU---
RNA1            AUGC-
RNA3            AUACG
                **
```

RNA1 OL964100 173 nt
RNA2 OL964099 190 nt
RNA3 OL964098 362 nt

All sequences in the cluster

1 Carpotroche-associated ilarvirus

Accession number / CP stop and 3'-adjacent 6 nt / CP-frame translation of sequence from immediately after CP stop codon to next in-frame stop codon (*), if present, or to end of available sequence

OL964098 UGA CCACAC PHSGGRCLVCEVSWGLQTYTRIDDFSLRDECLYCGADAEIGLLCPSFKCPQGCTQCL*

CLUSTER 35

Alignment of 3'UTRs from the 3 segments of a representative genome

```
RNA2              UAAUCAUAUU--------------------------------------------------
RNA1              UAGCU-------------------------------------------------------
RNA3              UGACCACACUCGGGGAGUCGUUGUCUUGUUUGUGGGACCUCUUGGUCUCUGCAGACUUAU
                  *

RNA2              ------------------------------------------------------------
RNA1              ------------------------------------------------------------
RNA3              ACUUAUCUUGAAGAUUUUUCUCUCCGAGAUGAAUGCCUGCUAUGUGGAGCAGAUGCUGAA


RNA2              ---------GUGUUUAUUCCAUUCAAAGACCCUCUUGGGCGCACUCAGUG-CUUAACUUU
RNA1              -----------------------------CCCUCCGGGGU-CACUCAGUG---UUAGUUA
RNA3              AUCGGACUGAUUUGUCCGUCAUUCAAAUGCCCUCUGGGGUGUACUCAGUACCCUUGUUUU
                                               *****  ***   *******    *   **

RNA2              GGAUGA-GUUUGUAUUCCGACUGUUUGUUUCUCCCAGUCGAUGCCUGUAGGCGUUUACAG
RNA1              AGAUGAUGUUUGUUUUCCGACUGUUUGUUUCUCCCAGUCGAUGCCUGUUGGUGUUUACAG
RNA3              GGAUGA-GUUUGUAUUCCGACUGUUUGUUUCUCCCAGUCGAUGCCUGUUGGUGUUUACAG
                   ***** ****** ********************************** ** ********

RNA2              ACGAGUACGGUUUUUACCGUAUGUUCCUAUUCUCUCUCUCAGGAGAGGAGAAUAGAUGCC
RNA1              ACGAGUAUGGUUUUGACCAUAUGUUCCUAUUCUCUCUCUCAGGAGAGGAGAAUAGAUGCC
RNA3              ACGAGUAUGGUAUUGACCAUAUGUUCCUAUUCUCUCUCUCAGGAGAGGAGAAUAGAUGCC
                  ******* *** ** *** *****************************************

RNA2              UCCAAAGGAGUCGC
RNA1              UCCAAAGGAGUCGC
RNA3              UCCAAAGGAGUCGC
                  **************
```

RNA1 EU919668 166 nt
RNA2 NC_038777 193 nt
RNA3 NC_038776 313 nt

All sequences in the cluster

1 Lilac ring mottle virus

Accession number / CP stop and 3'-adjacent 6 nt / CP-frame translation of sequence from immediately after CP stop codon to next in-frame stop codon (*), if present, or to end of available sequence

NC_038776 UGA CCACAC PHSGSRCLVCGTSWSLQTYTYLEDFSLRDECLLCGADAEIGLICPSFKCPLGCTQYPCFG*

CLUSTER 36

Alignment of 3'UTRs from the 3 segments of a representative genome

```
RNA2            UAAUUGAUCGA----------------------AUUCCUCACUU----------------
RNA1            UGAAGGGACCAUAUGGUCCAUACCGCUUGCGCGAUGCCCCUAACGGGAUG----------
RNA3            UGAAAGGUUGCUGCCUCGGGUGUAGUCCCCGAGAUGCCACACGUGGAUGGAAAUUCCUGC
                * *  *                           ** ** *

RNA2            ---------------------------------------------------------AUG
RNA1            ---------------------------------------------------------AUG
RNA3            GUGCGAUGCGGAUAUACACCCAAAUAUUUGAUAGAUUCAUAUAUUUGGACGUGUUUCAUG
                                                                         ***

RNA2            UGAGAAGACUAUCAAGAUAGGCUAUCUCUACGCUUCUUUUG-GAGAUACUUGAUAUGUUU
RNA1            UGAUGAGAUG----------GCAAUCUCGACGCUUCU--AACGAGAUGCUCAUCAUUUAC
RNA3            UGCUCAUAUGAGCAAUAUGCAUAGUUGUCACGCUUCUUAGACGUAAUUCUAUGUAUUCGC
                **   * *                *    ********     *  ** **    **

RNA2            CCAGUGUUAUUUAUU----------UAAUAACAC------------------------UG
RNA1            CAA--------CAUUAUCAAA----UGAUAAUGU------------------------UG
RNA3            AUACAAGUUUUCCUUCGCAAACUUGUAAUGAUGCCAUUAGUGUAUAUAUAUACACUAAUG
                  *          **          * ** *                           **

RNA2            AUGCCUUCCAUGUGGAAGAAGC
RNA1            AUGCCUUCCUAAUGGAAGAAGC
RNA3            AUGCCUCCCUAA--GGAGAAGC
                ****** **     * ******
```

RNA1 MG800789 147 nt
RNA2 MG800790 132 nt
RNA3 MG800791 260 nt

All sequences in the cluster

1 Peanut virus C

Accession number / CP stop and 3'-adjacent 6 nt / CP-frame translation of sequence from immediately after CP stop codon to next in-frame stop codon (*), if present, or to end of available sequence

MG800791 UGA AAGGUU KVAASGVVPEMPHVDGNSCVRCGYTPKYLIDSYIWTCFMCSYEQYA*

CLUSTER 37

Alignment of 3'UTRs from the 3 segments of a representative genome

```
RNA2            UGA---------------------------------------------------------
RNA1            UGA---------------------------------------------------------
RNA3            UGACUACACUCGGGAGAUUACCUGAGUGAGCUUGAGUACGACCCGAUGUCGGGUUGGUUU
                ***

RNA2            --------------------CUGUUGUUAU--GAAUUUUAAUGGUUAUUUCG--------
RNA1            --------------------GUGUUGUUGUUCCA---------GUGCGUUUG--------
RNA3            CUCAAUAGAGGAGUUAGAUGGUAUUACUGUCCAAUUCCUGCUUGUGUUUUUACCAACGGU
                                     * **  * *   *         **   **

RNA2            -----CUUUGGCGAAGGUCAUUAAUGCUAACUC-UUUGAGUUAAUGGUUAGUCACUGUGU
RNA1            ------------------CACUGAUGCUAACUC-UUUGAGUUAAUGGUUAGUCACUGUGU
RNA3            UAUCGCCUUGGCGAGGACCAUUGAUGCUAACUCUUUUGAGUUAAUGGUUAGUCACUAUGU
                                  ** * ********** ********************** ***

RNA2            GCGACUGCUACGAGAUGAACCUAUGGGGAUUCAAAUAGUGGUGAGAUAUCUUAAUCUGUC
RNA1            GCGACUGCUACAAGAUGAACCUAUGGGGAUUCGAAUAGUAGUGAGAUAUCUUAAUCUAUC
RNA3            GCGACUACUACGAGAUGAACCUAUGGGGAUUCGAAUAGUAGUGAGAUAUCUUAAUCUGUC
                ****** **** ******************** ****** ***************** **

RNA2            GGUUGCCCGAAGAUCGUAUGAUAUCGACUCCAGAUUAGUAUGGAGAUUCUACAUUUAACU
RNA1            GGUUGCCCGAAGAUCGUAUGAUAUCGACUCCAGAUUAGUAUGGAGAUUCUACAUUUAACU
RNA3            GGUUGCCCGAAGAUCGUAUGAUAUCGACUCCAGAUUAGUAUGGAGAUUCUACAUUUAACU
                ************************************************************

RNA2            UACGUUAAAUGUAAAGUAGCUAAGGCUACC-
RNA1            UACGUUAAAUGUAAAGUAGCUAAGGCUACCG
RNA3            UACGUUAAAUGUAAAGUAGCUAAGGCUACC-
                ******************************

RNA2            -
RNA1            A
RNA3            -
```

RNA1 NC_040435 248 nt
RNA2 NC_040436 264 nt
RNA3 NC_040437 360 nt

All sequences in the cluster

1 Tea plant line pattern virus

Accession number / CP stop and 3'-adjacent 6 nt / CP-frame translation of sequence from immediately after CP stop codon to next in-frame stop codon (*), if present, or to end of available sequence

NC_040437 UGA CUACAC LHSGDYLSELEYDPMSGWFLNRGVRWYYCPIPACVFTNGYRLGEDH*

Supplementary Data D

CP amino acid sequences from the representative RNA3 accessions (one per cluster, as defined above). Sequences were aligned with MUSCLE [7] with default parameters. All cysteine and histidine residues are highlighted.

```
MN386958        --------MSSSNKGTSVSANNRQSAGPNNAPQGGNAPQGKINA----------PSGRRV
JN107639        ----------------MSSSRG--VRSVNRCPTHVDELDCSANNC--RWTPAGSVNARQR
OL539725        -----------MNSRVRDKSLFGITSGCTNTSIITPDHPSNVMSSSQGSSGQ--VSNRQR
MG800791        -------------------MSSSGKNGQ--------------------------VTSKQR
NC_005854       --------------MYELFNHSLQPNQLTPCLRTRANGGQ--------------VSNRQS
OM323991        -------------------MSSQRNSGQETT-----------------------VSNRQR
OL472062        -------------------MSSSKGNGQCQCFDDLDAAANSCRRCNGRKANSQKPSNRDI
NC_027930       -------------------MSARGNNNNHVCQHRFDELDAASSRCEICHP----VSNRQR
NC_011555       MSTLTKNPDQPSNAMSANRGRNGISNSNRGCPNCFDNLDAAASNCTRCNPA---VSNRQR
NC_008706       --------------MSVNRGKNVLNNNSNGCPTCFDKLDAAATNCARCMRNGG-VSNRQR
OL584350        ----MITSTTNPDLPSNAMSTSRGKNNNGKCPIHFDELDALAHNCNCVGGS---VSNRQR
NC_003845       ---MNTLIQGPDHPSNAMSSRANNRSNNSRCPTCIDELDAMARNCPAHNTVNT-VSRRQR
NC_040394       -------------------MSTSRSRNNNQCPTCFDELDAAANNCSRHGGVNS-PSNRQR
NC_022129       -----MNTLNKSPDLPSNAMSTSRSFNNRNCQVCFDELDALANNCPRHGKVNQ-PSNRQR
NC_006066       ----------MSNAFCASCGLRSHVGNCPPRRKA---SPGQKNRT-RIYALER-KRANVR
OL964098        -------MNAQGNAFCALCGGAKLPNMGRRCQ-----PPCAG------------SAPRRG
NC_038776       -----------------MSTAVVRV-----------------------------LPRGVG
OL472059        --------------MATNNNCPVTLNGRTYVPAG-NNAP---------------ARQGRR
ON932436        -----------------MSNNAIEINGQWYIPQSGRQPKSVNNAP---------VIRGRR
NC_003810       -----------------MSGNAIEVNGQWYSPVTNNNAPSR-------------GRGRGR
NC_011807       -----------------MSGNAIEVNGRWYTPAP-NNAPTR-------------GRGGRR
NC_009536       -----------------MSGNAIEINGRWYQPAP-NSAPTR-------------GRGGRR
NC_003570       -----------------MSGNAIEVNGRWYQPAP-NNAPTG-------------SRGGRR
NC_003546       ---------------MANRSNAIEVNGVWYNRA--DNAPVA-------------NARGRR
NC_003835       ---------------MSNFSNAIEVNGKWYSPV---NAPP--------------NRGGRR
NC_039076       ---------------MSNTSNAIEVNGKWYSPM--NNAPA--------------NRGGRR
NC_040437       -------------------------------------MSTN-------------TQQKKT
NC_003453       -------------MLKMNAPQNKGKKKQNARTTQFAQRRAA-------------AARGEI
NC_003480       -------------MVCKYCNHT-HPGSCAGCKWCHSTNRFAPPKR---------AVARQA
HG328285        -------------MVCKYCGHT-HPGACAGCKWCHGTSRSAPPKN---------AVSRAQ
NC_022252       -------------MVCKFCNHT-HAGGCGQCKKCHGTKAAGPSVK---------AQDRAK
KU947035        -------------MVCKLCNHT-HARGCAKCKKCHPQGAAPPSAQ---------NVQRSK
NC_025481       -------------MVCKLCGHT-HAGGCVKCKKCFPTGAAPASER---------ARQRAR
NC_040470       -------------MVCNRCHHT-HAGGCRSCRQCHPRDAAPPP-----------PRARAR
NC_004364       -------------MVCRICNHT-HAGGCRSCKRCHPNDALVPLR----------AQQRAA
MW962375        -------------MVCRRCNHT-HPGGCDKCKKCHPTTASSSNPR---------QVARAA
NC_006568       ---------------------------MAFCNVCGKQMPCG-------------VVHRRG
MN527504        ---------------------------MAFCNVCGKQMPCG-------------VVHRRG
MZ170698        ---------------------------MTFCSTCGKRMPCGMNHN---------KSKGKS
NC_008038       -----------------MSGKAIKSGKPTTRSQSF-------------------ALARKN
KX196164        -----------------MSGKAVSKGKPTARSQSF-------------------ARQRKM


MN386958        RNQQRAARWRSGLASGQ-------------AGNVNVPL-PVP--------VTITGPMNGP
JN107639        RNARRAATFRNNQA-------------------ASVPL-PVP---------V--RVTQAP
OL539725        RNARRAAAYRNKLQQQP-------------IAKVPVPV-PVV-----PTRPIPVYPASQQ
MG800791        RNAQRAAKFRNSQPA---------------AAKIPLPV-PVV------------SVSRPQ
NC_005854       RNVRRAAAFRNSQQT---------------SARIPVPV-PVI------------PVSRPN
OM323991        RNARRAASFRNS------------------QAAARVPL-PVPVVPTGPISVI--PASRSQ
OL472062        RNQRRSAAYRQKLA----------------AANTPLPV-PVV------------QVCRPN
NC_027930       RNQRRAAAFSNAITNN--------------NASQNVRR-PVP------------VIPVGN
NC_011555       RNARRAANFRNQRKFDGLR-----------ALQAPVPL-PVV------------PVPQPA
NC_008706       RNARRAANFRNQ-----------------------RPV-PVV------------PVSAPT
OL584350        RNARRAAMFRN-------------------AQAKPVVVGPVY------------NQVSRP
NC_003845       RNAARAAAYRNA------------------NARVPLPL-PVV------------SVSRPQ
NC_040394       RNARRAAQYRNMQR----------------VATQSVPL-PVP------------VAPVIR
```

```
NC_022129       RNARRAANYRN-------------------QTAMVRPV-PVV------------PVSRPK
NC_006066       VPPGTTLNPKFFAQFSGYNAEECDLLDDVINAIPSVSV-SAA--------SQ--KRVTEA
OL964098        KPTNRSRNYAMNRQRNS-------------CMQATQPMIGTI------------PVSLGY
NC_038776       KPTQRSRNFAAQRQRSN-------------AMQATQPMIGSI------------PVALGY
OL472059        KPTQRSRNFA--------------------AARRSTPP-PML------------MGCHPT
ON932436        KPTSRSQAWA--------------------QGNRSQPPDRMM------------FGAMRS
NC_003810       GPTARSRNWAQSRA----------------NAARSRPQ-TLV------------IGTMPT
NC_011807       QPTARSRQWAQGQA----------------NARRSQPQ-ALM------------IGSVPT
NC_009536       QPTARSRQWAQGLA----------------NVRRSQPQ-QLL------------VGSMPT
NC_003570       QPTARSRQWAQQA-----------------SVRRSQPP----------------LSVNSV
NC_003546       RPTNRSRNWA--------------------QGQRSQPQ-RMV------------VGSMPI
NC_003835       KPTARSRNWA--------------------VQQRSQPP-NMV------------VGSMPI
NC_039076       KPTARSRNWA--------------------QQQRSQPS-DMM------------VGSMPI
NC_040437       RQNKRSTQFGQNRAKQR-------------AQSTGESS-TVP----NFGTLRLEWVRRGP
NC_003453       ETSSRNITAR--------------------SGGPPVPR-SIR--------TE--WELVGP
NC_003480       NPNKGKIPVSVS------------------RAGRSIRR-GGQ------------LGRLGA
HG328285        ANPNKGKIPVRV---------------------FNSPS-VRR--------TA--WEVRGP
NC_022252       NNPNKGSPSGTKSPGAK-------------GAPKDKQV-QKR--------TD--WTVIGP
KU947035        NNPNRIGATSRV------------------GSSSSAPVVRTS------------WTVIGP
NC_025481       NNPNRVVTSRVG------------------SSSKGANA-PTS------------WTVNGP
NC_040470       AQNAVARTLARP------------------ATSAGEPR-RLQ------------WTVIGP
NC_004364       NNPNRNRNPNRVSSGIG-------------PAVRPQPVVKTT------------WTVRGP
MW962375        NNPNRIR-----------------------AVQQSQPR--TT------------WLTRGP
NC_006568       RPTQRSQNFARRGGMAPRP-----------NTANNLAM-PTA--------VRTEWEINGP
MN527504        RPTQRSQNFARRGGMAPRI-----------NTANNLVM-PTV---------RTEWEINGP
MZ170698        KPTSRSQNWAQHRKSGVGNSNT--------SGSRQLPK-ILP--------SRTDWVMHGP
NC_008038       NNTTPPAGFVKKQFPS--------------GSSKSISE----------------WMLHGP
KX196164        NAPLPSSSKRTSL-----------------SESSKSSE----------------WRLHGP


MN386958        KRDV-FRLPRGQMWVKIAAQSFSAK-PTTSN--DAIQLSSLIGLCSQI-TDEVKIFRLIF
JN107639        PRPD-FRLPNGQVWVRRNPTEFAAK-GDDAD--DAVKVGTLIDAIPEI-NADTKLYRVLV
OL539725        KAKNPLKLPNNQVWVRRDPGVWNAK-TNDTN--DAIPITTMLSGIPEI-RPETKIYRLIF
MG800791        GSKASLKLPNNQIWVCKKAKEWGAK-TSDYN--DALTFTSILEGIPEI-KDDTKNHRLII
NC_005854       GPKASLKLPNNQVWVYKVASELAAK-TSDAN--DAISLTTMLSGISDV-KPETKLYRLVF
OM323991        GSKSALKLPNSQVWVCKAAAEWGAK-TTDAN--DAIPIKTMFSGIPEI-KPETKFFRLII
OL472062        ESKSPFKLPGNQVWVSRKAEDWGAK-TAETN--DAIALTTIFRKFPEI-TPETKIYRLLF
NC_027930       SRPT-FRLPGNQVWIRLTASSWAAK-TVDTN--DVLPLKNIFNGINEI-DSETKIFRLLI
NC_011555       TQRN-LRLPNGQVWVTRKPTDWAAK-VNDAN--DAMLLKTIFDGITEI-KPDTKVFRVLI
NC_008706       TSKS-LKLPGNQVWVTRKPSDWAAK-TVDTN--DAITLKTIFNGIPEI-NDDTKIFRVLI
OL584350        VPKASFRLPNNQVWVTRKAGEWAEK-STDTN--DAITLRTIMEGIPEI-GEDTRVFRILI
NC_003845       AKAS-LRLPNNQVWVTRKASEWSAK-TVDTN--DAIPFKTIVEGIPEI-GAETKFFRLLI
NC_040394       SRRVNFRLPNSQVWVTRKASEWAAK-TTDTN--DAISLRTILNGIPEI-SDETKVFRLLI
NC_022129       TTAS-LKLPGNQVWVTRKASEWAAK-TVDTN--DAIPLKTIMDGIPEI-TEESKIYRLLI
NC_006066       QLVVGDPLKGIQGPCTHMGDATITA-RTGGVRGFYMALDALLTGIPDA----TRLKSLVM
OL964098        NLPT--NFPSGE-WHKMSGYSFGSS-SASLV--YYTTLEAELKKFPDLIHANTAVYSVLI
NC_038776       SLPV--QFPGSE-WHKISGYSFGST-STNYV--YYQTFEAELKKFPDLIHANTVVYSVLI
OL472059        HLPVWRSFPNEQ-WHEIGGFSFPKSWSGGNL--AIMNFKNEFDKVKSL-HSTTKTYSVML
ON932436        SLPSWISTPGEQ-WHEVEGLSFPASWTTGSV--ASASMRTELGKIRPL-HDSTKVYSVMY
NC_003810       NLPTWKSFPGEQ-WHVVSGFSFPDRWNNGNI--AYASMKTELGKIKTL-HEITKVYSVMY
NC_011807       SLPTWKSFPGEQ-WHEVSGYSFPNSWGSGTL--AWLTMANELNKIKTL-HDTTKVYSVML
NC_009536       NLPTWKSFPGEQ-WHEVSGYSFPDRWGSGTI--AYMTLRSELSKIRTL-HDTTKVYSVMI
NC_003570       PALTWRSFPGEQ-WHEVSGYSFPDKWSAGTL--AYMTLRAELGKIKTL-HDTTKVYSVMI
NC_003546       DTITWKSFPGEQ-WHEFSGFSFPDAWGSAKI--AYASMRTELGKIKSL-HHTTKVYSVMY
NC_003835       NLPTWKSFPGEQ-WHEVSGFEFPASWISGDI--AYASMRTELGKIKAL-HDSTKVYSVMY
NC_039076       GLPTWKSFPGEQ-WHEVSGFEFPTSWGSGDI--AYASMRTELGKIKTL-HDTTKVYSVMY
NC_040437       GDLDILQFPYQ--WVAKAESSLKAT-SNDIW--YIIDLHEYVKDMMEF---PTSVKGFVF
NC_003453       SVGA-RLIQGPHNEVVKGEAVASST-TAGQY--FSIQISTYLEKFLEM---GVQLNSMTV
NC_003480       RMLS-RKFLKG--HRVLSSREVTAT-VEGRF--VNIDFADVFRDLLEK---DLKVYTFII
HG328285        NVEP--RVPKG--HRVFSSREVTAT-VEGKF--VNIDFADVFRDLLEK---NLKVYTFII
NC_022252       NVQP-VNHPNG--FTLRSCRDVQAT-VAGKF--LHINFKTAFPQLLNQ---ELKIYSFAV
KU947035        NAVP--NVPRG--TVTRNYADVVAS-APSRY--LTVDFSDRFPHLVGN---KLRILSVLM
NC_025481       NTEPIV--PRG--TVLRSYTDVVAS-APGKF--LTIDFSERFPNLLGH---KVRILSILL
NC_040470       NEVP--RVPRG--YVAHSNREVVAT-SAGKF--LHVNFSTTFPQLLGL---NLRILSVVV
```

```
NC_004364        NVPP--RIPKG--YVAHNHREVTTT-EAVKY--LSIDFTTTLPQLMGQ---NLTLLTVIV
MW962375         NEVPLV--PRG--HVVKSVHEVTVT-SAGKY--LSIDFSAAFPQLQNQ---KLNVLSILL
NC_006568        NTQV-RRFPGL--VTVSKGVAIRST-GAGTY--WGFAISTAFHNLMGQ---RVLCDALVM
MN527504         DAQV-RRFPGL--VTVSKGVVIRAT-VAGSY--WGFSLATAFNSLNGQ---RVLCDAMVL
MZ170698         NVKP-KRFNGV--VSTSMGTAVTAP-KKGSY--YSISIKDCLRILGNT---DTTIFGIII
NC_008038        NVPV-KSFSGM--ISRTENLTVNST-ASGVY--YTMKVRELFKDF-AV---DTKVYGIVF
KX196164         NVPV-KRFTGM--VSRSENLSTNSV-ARGEY--FATQIKDLFKGHDSS----TKVYGIVF
                                                      .   .               .

MN386958         GFIA-KSDGHF-ALVE---EAGDNIPSLPVVGRVSFAEGVYRSREIHF-AGLTSSDL--S
JN107639         GFVA-VSDGIF-AFVR--DV-GDDIPNLPVVGRVGFTRQTYRSRSISL-NGAVASDC--R
OL539725         GFVA-ESNGSF-GIVEDEGVAGNTVPDPPVVGREKFQKHEYTSRDVNL-EGKTSDEL--K
MG800791         GFVA-ESDGSF-GIVEDEKVNDGTVPDPPVVGRMGFKKHTYKCRDINL-EGKTRDEL--K
NC_005854        GFVA-ESDGSF-GVVEDENVSGNVVPDPPVVGRAGFKKHTNKCRDVNL-EGKTPDEL--K
OM323991         GFVA-ESDGSF-GVVDDENVASNVVPDPPVVGRVGFKKHTYKCRDINL-EGKTPDEL--K
OL472062         GFVA-ESAGFY-GVVN--GVSGDTVPDPPIVGRVGFKKHCYRCRDVDL-GGKTTEQL--A
NC_027930        GFVA-MSAGTF-GLVD--GVTSTSVPDPPIVGRVGFEKNTYRSRDFDL-GGKTPLQL--D
NC_011555        GFVA-MSDGTF-GLVD--GVTGDTVPELPIIGRWSFQRDVYRSRDIGL-DGQPADQL--S
NC_008706        GFVA-VSDGTF-GLVD--GVVGGSIPDPPIIGRLGFKKNTYRSRDFRF-DGKLTPQV--A
OL584350         GFVA-KSDGTF-GMVD--GVTTDVVPDPPIVGRLGFQKNTYRSRDFHL-GGKLSTQV--A
NC_003845        GFVA-VSDGTF-GMVD--GVTGDVIPDPPVVGRLGFKKNTYRSRDFDL-GGKLLNQL--D
NC_040394        GFVA-VSDGTF-GLVD--GVTGDVIPDPPVVGRLGFQKNKYRCRDFDL-EGKTPPNL--A
NC_022129        GFVA-VSTGTF-GIVD--GVTGDVIPDPPVVGRLGFQKNTYRCRDINL-EGKTPSQL--D
NC_006066        HITFLSDGGVL-GLNT--E-RSDQYVEPNPLKRKRFEKESPFWAQILFPDNVKLSDV--K
OL964098         GFTA-KSDGYA-GFDN--AFDSAALKEPEAPNRNRFLNGKYCGLEMVMPAGTTASDL-NG
NC_038776        GFTV-KADGYP-GFDE--AFDSTALKEPEAPNRHRFLSSKYCGLEKVFPAGTVASDL-NG
OL472059         GFTC-LYDGYA-GFVA--GTEGKTPTAPVAPDRMRVKAGKYGARQHVYAPGTAVLDA--S
ON932436         GFTC-KSDGYA-GFVE--GFDVNNPTGPIAPNRVRVKAGKYAARQLRCPPGTTIADL-KA
NC_003810        GFIC-KSDGHA-GFIT--GFDTNNPTGPVAPDRVKVKKNAYCAKQQVFPAGTTVSEL-KN
NC_011807        GFTC-NYDGYA-GFVE--GFSTSNQIHPIAPDRIRVKKGKYGARQQVFPTGTTVSEV-RS
NC_009536        GFVC-KSDGYA-GFMD--GFDVNNATGPVAPDRIRVKRGKYCAKQQVFPTGTTVSEV-KA
NC_003570        GFVS-KSDGYA-GFMD--NFDANHATGPVAPDRIRVKKGKYCAKQQVFPTGTTVSEV-KA
NC_003546        GFTC-KADGYA-GFMD--NFDSANATGPVAPNRIRVKAAKYCARQLVLPPGSTVADL-RD
NC_003835        GFIT--KAGWLCRLKD--DFDPSNATGPNAPNRTRVKAGKYSAKQLVFPPGTTVAEV-KS
NC_039076        GFIC-KADGYA-GFKD--DFDPSNATGPNAPNRIRVKAGKYCARQMVFQPGTTVSDV-KS
NC_040437        LFEA-NLSGQA-CLVT--KKTGSFKDAFSSLNAFKFEKGCHTAVQLLAPSILTFATFSAG
NC_003453        LCASLQGSGIV-CLTH--KFTTCEA-AISTLSGLRFREGRRLGFQVLPPSDTVAGDV-AS
NC_003480        RVNSLSSNGWI-GLVE--DYDESNPKGPNPMDRKGFKKDQPRGGMWEAPPNTTFDDF-VR
HG328285         RVNSLSSNGWI-GLVE--DYDESSPKGPNPMDRKGFKKDQPRGWQWEAPPNTNFDDF-VK
NC_022252        RCSTSIGNGWV-GLVR--GFNPSSPTGPAVLTRKGFLKDQARGWQWLAPSDLEYDKF-SE
KU947035         RVNSFHSGGWV-GIVE--DYDVSSPKGPDPMTRKGFSKDQARGWQWLAPSGLEFDDF-AR
NC_025481        RVNAFHSNGWV-GLVE--DYDVSSPTGPDPMRRKGFMANQARGWQWMAPSGLEYDDF-AK
NC_040470        RASCLVSAGWV-GMLE--DHDENHLRGPSALSRKGFRQDQPRGWQWLAPSDLEYDRF-AN
NC_004364        RMNSMSSNGWI-GMVE--DYKVDQPDGPNALSRKGFLKDQPRGWQFEPPSDLDFDTF-AR
MW962375         RLNSFHSNGWV-GLVQ--GYDASNPRGPDALNRKGFLQDQPRGWQWLAPTNLEYDDF-VR
NC_006568        RFISDHSGGVV-GVVR--GYNPDHPTAPNALTRRRFHKGVATGLQFLAPTNQVVDEI-AD
MN527504         RFVSDHSGGVV-GVVR--GYDLDHPTAPNALTRRRFHKGVATGIQFLAPTNQVVDEI-AD
MZ170698         RFCSDHASGVF-GLVK--DFTPTDPIPPNPLSRRKFVKGEATGIQLLAPINLKVNDV-NS
NC_008038        RYCLDVSNGVY-GLIK--GFDVNAPVAPNPLQRRKFTAKQASGVQILAPTGMTVGDI-PD
KX196164         RYCLDFSNGTF-GLIK--EFDVNNPTEPNPLSRRKFIKMCASGVQILAPTDVTVADI-PD
                         *    .                     .

MN386958         ELKLVWDLKDDNSRKAAGR----IETSDYWIAISRPNDVYPPGDILRNSD----------
JN107639         NHAFLWSL--NDHKRDARR----VVTADYWFAISKPAMLMPPEDFLENSN----------
OL539725         YKAVIWCL--DENRRAEKR----VSLTHYWFAISRPPPLMPPADILVNGNGNY-------
MG800791         NRAIVWCL--EENRKAAKR----VAMTHYWIAIDRPPVLMPPEDILVDGNQ---------
NC_005854        NMAVVWCL--DENRKVAKR----IAFTHFWFAISRPSPLMPPENILVDGNQ---------
OM323991         HRAVVWCL--EDNRKVAKR----VMMTHYWFAISRPPPLMPPENILVDGNSLS-------
OL472062         DKAIVWCL--DENRKAAKR----VTLADFWVAISRPAPLMPPDDFLVEGDQ---------
NC_027930        GKAVVWCL--EEHRRDEKR----VQLADYWVAISRPQPLMPPEDFLVNSQ----------
NC_011555        EKAVVWCL--NTNKRAEKR----VRLADFWVAIAKPKPLMPPPDFLVEDN----------
NC_008706        DKAIVWCL--DSNRRDAKR----VRLADFWIAISRPNPLMPPDEFLSGDSD---------
OL584350         SKAIVWCL--DDKRREAKR----VSLANYWLAISRPAPLLPPEDFLVEGSN---------
NC_003845        DRAVVWCL--DERRREAKR----VQLAGYWIAISKPAPLMPPEDFLVNQD----------
```

```
NC_040394       DKAIVWCL--DSGRRDAKR----VSLANYWLAISRPTPLMPPSDFLVESD----------
NC_022129       GKAIVWCL--DSNKRDAKR----VMLANYWLAISKPAPLMPPEDFLVESSD---------
NC_006066       NYAIMIKF--DSDYSANTP----LYTRECWVNHYQLPSAVIPEEAFARK-----------
OL964098        SHAFVWAI--DAAFPTTMPANSGIKVHSMWWQTAKLPPMKPPQNFLACEK----------
NC_038776       SHAILWVI--DAAFPSTMNANSGIKVHSIWWQTAKLPPMKPPQNFLQCEK----------
OL472059        DLWFVWQF--DSTPKDGDSNV--ITVTKIYISTMPLPGLKPPPNFLVCEE----------
ON932436        SWSFVWQF--DAAPATATVNQ--ISVVGFWVSTTPLPGVKPPPDFLVCEE----------
NC_003810       DWSFIWEF--DAAPVVGTEKQ--VTVTQFWVSTTPLPGVKPPSNFLVCED----------
NC_011807       NWTFVWNF--DAAPPTGSKDA--ITVTKFYIATSPLPGVKPPSNFLVVEE----------
NC_009536       NWNLVWDF--DTAPATGAVHE--ISITKFYVSTTPLPGVKPPSNFLVVEE----------
NC_003570       NWNMVWDF--DTAPATGSERE--IFITRFYVSTTPLPGVKPPANFLVVEE----------
NC_003546       NYNFVWEF--DAAPAAGTANI--ISVTKFYVSTVPLPGIKPPPNFLVCEMDDK-------
NC_003835       AYNFVWQF--DSAPASGTANV--VKVVKFYVSTTPLPGVKPPANFLVCEE----------
NC_039076       SYNFVWQF--DAAPAANAPNM--VKVVKFYVSTTPLPGVKPPSNFLVCEE----------
NC_040437       EVALVFKF--EGTLTAGAE----FMTRKVWGQSSMLPKVEINKNLLRGQ-----------
NC_003453       NVRLVFKF--DKAFEANAP----LIERKVWVSTSRLPEIMIPANLLVPDED---------
NC_003480       KFRLVLEF--KTNFAAGAK----VFMRDLYVITSELPPVQIPTNVLLIDEDLLEL-----
HG328285        KFRLVLEF--KTNFAAGAK----VFMRDLYVITSELPPVQIPSNVLLIDEDLLDL-----
NC_022252       EYELVFEF--KSDYPIGV-----VMTRDLYVVTSSLPRVRIPDDLLFVDEDLLEI-----
KU947035        THRLVFEF--RTDFAATAK----VLCRDIYVVTTELPRMMIPSDLLFVDEDLLED-----
NC_025481       KHRIVLEF--KTEFAAEQK----VLTRDLYVVTTELPKVTIPGDILFVDEDLLDV-----
NC_040470       SHRLVFEV--KNEFTAGVK----VLVRDIYIVVSDLPRIVIPNDILMVDEDLLDV-----
NC_004364       THRVVIEF--KTEVPAGAK----VLVRDLYVVVSDLPRVQIPTDVLLVDEDLLEI-----
MW962375        SQRLVFEF--KTNFVANTK----VLSRELYVVVSDLPRVEVPRDLLMVDEDLLEI-----
NC_006568       DIYIVFQF--DTAFTANAV----LLTRDRYLQHNAVPRVEIPADVLRTEALPVEDIRG--
MN527504        DIFVVFRF--DTAFVANTT----LLTRDRYLQHNAVPRIEIPADVLRTEAMPVEDQRG--
MZ170698        NTFLVLKF--NTDFEAGAI----LWMRDMYLQHSAPPKVDIPESVLYTDSLPTEEVD---
NC_008038       DLWFVIKY--DNAFQPNVP----VWFCTQYLQHSMPKRVEVPDSVLYAERDTALMDAMDK
KX196164        DLWFVIKY--DKSFDANVP----VWVRTQYLQHSMPPKIEIPDSVLMAERDSILVDAMDK
                   .:                  .     :             . :

MN386958        ----
JN107639        ----
OL539725        ----
MG800791        ----
NC_005854       ----
OM323991        ----
OL472062        ----
NC_027930       ----
NC_011555       ----
NC_008706       ----
OL584350        ----
NC_003845       ----
NC_040394       ----
NC_022129       ----
NC_006066       ----
OL964098        ----
NC_038776       ----
OL472059        ----
ON932436        ----
NC_003810       ----
NC_011807       ----
NC_009536       ----
NC_003570       ----
NC_003546       ----
NC_003835       ----
NC_039076       ----
NC_040437       ----
NC_003453       ----
NC_003480       ----
HG328285        ----
NC_022252       ----
KU947035        ----
NC_025481       ----
```

NC_040470	----
NC_004364	----
MW962375	----
NC_006568	----
MN527504	----
MZ170698	----
NC_008038	IVSG
KX196164	IVYG

Supplementary Data E

Sequences of the asparagus virus 2 clone used in this study.

atg ORF start codons
taa ORF stop codons
G 5′ end of the AV2 sgRNA4 as determined by RACE-PCR

>RNA1

```
gtattgtgcagtattatttacgcacgacccggattaatcagttttctgagtatatacccgatagaaactcaatggatac
gatcgtgcagcatgaatctaccgtgttacgtgtcgatgacctgattaatgatacgatcaggagaaatgctgctcgtccag
aaacagctgttggcaggcttgtttctgatgccgctgtgcaagtcgttgctaggcaatgtgaccttacaccagcgaagcct
cttaatgtgtcttttcaattgtcaccggaagaccagaatgcattaaggagagattttcctggaagagagctccaatttag
gaattcgtcatattcttctcactcttttgcggctgcacaccgcgtttgtgagacggattatatatatggacgcttccaga
ctgaaaataccatgatcatcgatataggaggaaattttgcacccatagtaaaatgggtagggataatgttcattcgtgt
tgccctatattggatgttcgtgatggtgctaggtatacggatagatttatgtcagttgctggatcattggaaaaacaacc
agatcgtgaattacggttgaattattgttccaatagattcgaagaatgtgatatttccgctccttgggcgatggcaatac
attctatttccgatattcctatcactaccgtggttaaacattgttttagaagaggagtgaaaaaactcattgcttcgata
atgatggatcccatgatgctcatcgctactaagggtttcattcctcgacttaatgtccaatgggaaattgaaaccgaaag
cgatggaaagtcccgacgtatttcttttcatttttgtcgatgctcccggattgtcatattctcataactatgatgtgctaa
tgcagtacatgacttgtaatcaagtcataatatcagggaaagccgcgtacagagttgaacgcgttgcagatctatcgggt
gttttcattgtcgagatcacccttgcagctactgctggtattgagcgtttagatctagttcccatgcgcgatgtttcatg
cgcttggatgacgtctttaagacggaagaccttggtgagggtggccgttccccagtataggcatgcatgggagataaaat
acatcattgctgacacagattttgtcagaagagtggctgaagtttcattccgtcaatataagcctgacaccaaattggaa
gatttggtgaaatccattgcgacgatgatttcctcttcttcgaaccattgcattgttaatggcgttactatgcagactgg
cacaccagtagccattgaggattatgttccaatggcagttactttcgttgcctttgctatatcacgtcataagtccattg
ctaaaggaattaaaatggtggagcagagaggtactaacataatggacccaaatgatcattatgattatgaaaaggatgga
tccgatgttaagagtttttccaaccacattattcccatgaagaataccatcaagtccttattttcccctcgaaagcgaa
aatcagtgataattcaatgttgatttacagtcaaagtctcatgtctactgttgttgatgagattaaaacgcttttggat
gggatgtttgggatactgatgatgcagtcatacaatcattgccctcctttataaaatggaggatgtgttccaagttacc
tccgaccattattgtttgtcacacacgttgtctgttgattattggttggagggtttgtatgatagctatgatgcccttcg
taaggtgcataagaaaaagcttgaagaggaggaggctcataaaacaaaggttgaaaatgccctcctaaaaattgctgagg
tcttggagaaacccgatgttgatgacgggttgaaacagttgaaaacaatacctctgataaattcattattggagaagaaa
acggaagaaattgttacaaagcctcaatgcagggattctgagaaaccccacattaatccctatgctgatgcaatcaagga
agctattgcttattaccatgagcttgaggtggtaaatacgcgtaatctgagaggtgttggtgattacctcggatggcgca
cgaaaacgaactatgccagtgtgtgggctggtgaagagtccagaatggtgctagaaccaatggccagaaagttccattct
aaggaccgtgatgttcccatacccgagtatgagcgaggtatgacggttgatggtttcatttcccttgcttggaaggatgg
tgaaatcacaagggagacgtggaaatcccttgccaagtacaacgttgttttgttcgatcaatcgtgtgtatttgatgccg
gttcacgacttttaccagggttgaacaaagcccttgtgatggatgcttcttttaaagtcgttatcgaggatggtgtggct
ggatgtggaaaaacaacatctctactcaagcaagcgaaacctgattcagatttgttgttagcggccaaccgtgagactgc
aaaagatgcaaaaaccagcggagttatcccggatgctctattgtaccgagttagaactgttgattcttacttgatgctca
agaactggtttactgccgaccgtttattggttgatgagtgtttcctagttcatgctggtttgatctatgcagctgccacc
ttggcacgagtgaaggaggttattgcttttggtgatactaagcaaataccattcgtgtctcgtattccaacggtcacttt
acgtcacgcttcagtgatcggtgttttgaagcccaggacaataacctatagatgtcctagggatgttaccgccattttga
gcgagaagttttataataccaaggtgaaaactttcaacccagtgaaaacttcattggatttgattaatatcaattcagga
atggaaattcccgtggtgaaagatgctctatatatagctcacactaaagctgataagtttgccttgaagaaattgccagg
aatggggaacgttgatgtgctcaccactcatgaagcgcaagggaaaacgcgtgacaatgttatccttgctagactaagta
ggactactcatttactgtattccgggaagatgcctgatgctggtagttcacataacttagtgggtttgtcccggcataag
aagagtttgaaatacttttctgtttttgccgatgatccagatgatcaaattgcctctggaattcgttggtctaaaacttt
agacgaacaagagctgtccgcgtatagggcagctaatagtgtttttttccatgaaacctagtgaagggtcctccgggatgt
gctcagcacctagcctaagctaagtccatatgcccatctttgctgctccggatggatgtttatacccgctatggatgcct
attactgaaatgtaatagatgcctaatactctctctcatggagagagtttagatgcctccaaaggagatgc
```

>RNA2

```
gtattgtgcagtattatttacgcacgacccggattaatcagttttctgagtatatacccgatagaaactcaatggatgc
tcattatctcctttccgaattgtgtgacataaggaaatttgtctcaactcgtgttattgaacagagttttgacatcaagg
atgatgagactacaagctatcttcaagcttggatgtattcccgcattcttgtgcctgttatgaagttccacctttgtggt
gaagttctgctggacaagattgaggatgtgttccgcgaaactcttggttgcgaagccgagcccaatgatgatggttacat
gccagttttcattcatcttgatgatgacgatgatgaacctccaccagaagcttacttgccaaaggaagagcgtgaagcaa
tacacgcggcaacgtttctaggtcatgaacattgggctagtgttacctctggatctgattttatgatgacattgagatg
ctcgctgatgagaccatctatgatgatttgcagactgataagtgtgcggaaatgcccgctgattatcaaatcatgtggaa
tgatggtgccatagatgctgtgtggtctagtcagtttgaatgtgaagaaccacccaaaccaaagttcaaaccccagtcgg
ttgacaagggatgtgatccagtggttattcaagcagcaattgatgatatattcccatttcatcatgaaatggatgatagg
```

tatttccaaacattagtggaaacgcaggacatttcccttgaagtctctaagtgttggattgatgcttcgaatttcagaga
tttcgttaagggacaagattcttatgctgttcctgtgatacaatccggtgccactagcaagcgtgttcatacacagcgtg
aagcgctattagcagtgaagaaaaggaatatgaatataccggaattgcaatccactttttgatcttgatgcggaagtcaac
acgtgttttaaaaggtttcttactcatgttgttgatgttccaagattgaggagacttcaaccgatgactggtgttgaagt
ggaatttttttaaccaatatttattggggaagaatccacctctcaaagaataccaaggaccattaccactatgttcgcttg
ataaatacatgcacatggtgaagactattgttaagccggttgaggataactcgttaaaatacgagagacctctttgtgcc
acgataacttatcataagaagggtatcgtgatgcagtcctcacccctttttttgagtgctatgtcaagattgttttatgt
cttgaaatcaaaaattcacatacctagtggaaagtggcatcaacttttcactttagatgctgagaaattcgacgctgcta
aatggttcaaggaagtcgatttctcaaagtttgacaagtctcaaggcgaacttcatcataaagtgcaggagaaaattttc
gaatgtttgaaattacctcctgggtttgtggagatgtggttcactgcacatgaacgatcacatattgttgatagagaggc
tggtattgggttttccgttgattttcaacgacgaactggagacgctaacacatatttggggaacacattggtgacccttta
tatgtttagctagggtttatgatttgtgtaacccgaatatcacattcgttattgcctcaggtgatgattccttgattgga
agtgtggatgagttgcctcgtggtcctgaggatttattcactactttgttcaatttttgaggctaagtttccgcataatca
accttttatatgttcaaagtttttagttagtgttgatcttgcggatggtggtcgtgaagttattgccgtgcctaacccag
cgaaattgttgattcgtatgggtagacgtgactgtcaataccaagccttagatgatttgtatacctcgtggcttgatgtc
atatattatttccgagattcccgtgtttgtgaaaaagttgcttctctatgcgcttttagacagacgcgaaggtcatcgat
gtatttattgagtgctttgttaagcttacccagttgttttgctaataaaaaaaaattcaaatggctctgttatcgtttaa
cagagcaagagtgtctgaagattacaaaacaacctggaagttctcattatactaagaaaaatgaatgctcaaaggaaagt
gtcaaaaatttcccacaccgacggagctcgcgtggacgctgggaatttattcccaagattaagtccgatgatgattaccg
gatttttcctaatggtgatcttagcaccattagaggctcgtcagtttccaagacagactgcggttcaggaaagtgctccg
acaccgccagctcaattggtaataaaaataccagacctcgcaatcgatttcgagctgaaggagttcaccaacccagcggt
tgttattcaaaccgtttaccgccgaatcatcggcgaagttcctaaaggttggtatggtttgaagtcctggtcttcctcca
attatggagatgtggtgacagggctgaaaagggccgcacgggctaaagtgcatttttctattccgggttctgattgggct
tatacactcaatctatccgatgtggtttctgggcttgcactacccaagttaccaattcctgagaaatatctcaagatgcc
catttcttttgaaatggatgattccgtctttggtgacagaatttctgatcgagtaccatctccatggtatctcggtcaat
gacgtgtttctttcatgaaacctagtgaagggtcctccgggatgtgctcagcacctagcctaagctaagtccatatgccc
atcttcgctgctccggatggatgtttatacccgctatggatgcctattactgaaatgtaatagatgcctaatactctctc
tcagggagagagtttagatgcctccaaaggagatgc

>RNA3

gtattcaactgagtttaaataccctttcgagactaatagaattcaatcaaacgagtacgagatcaatcgtgaaatttcta
ataggcgtcagaaatttcggtttcccactacctacttaactgtggattcagtggattttttccgtttgattaagtccgcaa
gtttgttaagaggtggagtcctcttttattagtcttgcaagaaagacacgtgaacgttcattctccgtgtaattgtgaat
actctcgacttgtacgggaaagccctcacaaacgctattcctcttagtgttgggttctgttgaggagtactagctcgaaa
gaaacttcgaaaatcgccgaagtttattattatggccttggttaaaacaaaacctctcgtccttacagccgccaatgaag
aacagttgtacaagcaaatttctggcgcgctagaatccgtgaacaagaatttaaaaatgtttagagcctgtgtgccacta
gagttaaaaaataatggttttacatcatttgaactctgtgatgaagatacacgttcccttgtaacagcatttttctgtcaa
agtccgaaagaacattaatgttgatcatccccacatttttcctcttgtgggttcctcggattttgaaatctacctcagcca
ccgcgattattaaatgtaaatatctggcgactggagatgagaaagcagttgggaagtttccgctcaatgaagccttcatc
ttttcttttggatgggaaagatccattaggatgagggatgcttatgatgggaagggtttgcacttgtttattcagtgctt
tgcccctaattctgaagcgaaggctcccttaggcaggttagtccctatgtgggataattgtgccactgctaaaatgcgtt
actcggaagatgtgggttcctcacttaccacagcagatgaaatgcgtgtgagaaatgtgcttactgacaaagacacgagg
aacctgctacgatcttatatggctaccgagttcgcttgtcgcgaatttgaagataagttcacaggtgcaccgagagtgca
attatcggacgatttacccgagacgcttgattttacacgtcttggagggccgacacctgaatgttcagatgctaccgaaa
ataaacgaagaactgttgtttgtggaaatggtgtagtggagattgcgatggaagcgcagtctcgaaatactccgtagtgg
tgagttataaagtctcgtgggatggatacccttatttgggtcccccggggactgcttgtccgggaggagatttgtcttga
agtttccttctccactttccgtcacctattcttccactacaacaacatgtctggtaatgctattgaagttaatggtcgtt
ggtatactccagcacccaataatgccccgaccagaggtcgtggaggtcgtcgtcaacccactgctcgttctaggcagtgg
gctcaaggacaggcgaatgctcgtcgatcccagccacaggcgctgatgattggttccgtgcctacatctttacccacctg
gaagtctttcccaggggaacaatggcacgaggtttctgggtattctttcccaaattcgtggggttctggaacattggctt
ggttgaccatggccaatgaattgaataagataaaaaccctacatgataccaccaaggtttactcggtgatgttgggcttc
acttgcaactatgatggttatgccggctttgtcgagggtttcagtaccagtaatcaaattcatcctatcgctccggatag
gatacgagtgaagaaagggaagtacggtgctagacagcaagttttccccactggtactacggtttccgaagttaggtcca
attggactttcgtttggaacttcgatgctgctccccctactgggtcaaaggatgctatcactgtgacgaagttttacatc
gccacttcccctttacccggtgtgaagccaccaagtaatttcttggttgttgaggaatgaccacgctcgggtgagtgtgt
atacctccattctaatgaatgtgagaagaagaatgtgattccattctgtcattgtgaatgttatgacaatgcttgcaaca
ccttgtggtgttctgcaaatgtcttttgtttccacgaaccttaatggttagtgatgaagtgtttctttcatgaaacctag
tgaagggtcctccgggatgtgctcagcacctagcctaagctaagtccatatgcccatctttgctgctccggatggatgtt
tatacccgctatggatgcctattactggaatgtaatagatgcctaatactctctctcatggagagagtttagatgcctcc
aaaggagatgct

Supplementary Data F

Sequence of the AV2 RNA3 with the AV2 RT sequence replaced by the TSV RT sequence.

atg ORF start codons
taa ORF stop codons
G 5′ end of the AV2 sgRNA4 as determined by RACE-PCR
nnnn TSV-derived sequence

>RNA3-TSV_RT
gtattcaactgagtttaaatacccttttcgagactaatagaattcaatcaaacgagtacgagatcaatcgtgaaatttcta
ataggcgtcagaaatttcggtttcccactacctacttaactgtggattcagtggatttttccgtttgattaagtccgcaa
gtttgttaagaggtggagtcctcttttattagtcttgcaagaaagacacgtgaacgttcattctccgtgtaattgtgaat
actctcgacttgtacgggaaagccctcacaaacgctattcctcttagtgttgggttctgttgaggagtactagctcgaaa
gaaacttcgaaaatcgccgaagtttattattatggccttggttaaaacaaaacctctcgtccttacagccgccaatgaag
aacagttgtacaagcaaatttctggcgcgctagaatccgtgaacaagaatttaaaaatgtttagagcctgtgtgccacta
gagttaaaaaataatggttttacatcatttgaactctgtgatgaagatacacgttcccttgtaacagcattttctgtcaa
agtccgaaagaacattaatgttgatcatccccacatttttcctcttgtgggttcctcggattttgaaatctacctcagcca
ccgcgattattaaatgtaaatatctggcgactggagatgagaaagcagttgggaagtttccgctcaatgaagccttcatc
ttttcttttggatgggaaagatccattaggatgagggatgcttatgatgggaagggtttgcacttgtttattcagtgctt
tgcccctaattctgaagcgaaggctcccttaggcaggttagtccctatgtgggataattgtgccactgctaaaatgcgtt
actcggaagatgtgggttcctcacttaccacagcagatgaaatgcgtgtgagaaatgtgcttactgacaaagacacgagg
aacctgctacgatcttatatggctaccgagttcgcttgtcgcgaatttgaagataagttcacaggtgcaccgagagtgca
attatcggacgatttacccgagacgcttgattttacacgtcttggagggccgacacctgaatgttcagatgctaccgaaa
ataaacgaagaactgttgtttgtggaaatggtgtagtggagattgcgatggaagcgcagtctcgaaatactccgtagtgg
tgagttataaagtctcgtgggatggatacccttatttgggtcccccccgggactgcttgtccgggaggagatttgtcttga
agtttccttctccactttccgtcacctattcttccactacaacaacatgtctggtaatgctattgaagttaatggtcgtt
ggtatactccagcacccaataatgccccgaccagaggtcgtggaggtcgtcgtcaacccactgctcgttctaggcagtgg
gctcaaggacaggcgaatgctcgtcgatcccagccacaggcgctgatgattggttccgtgcctacatctttacccacctg
gaagtctttcccaggggaacaatggcacgaggtttctgggtattctttcccaaattcgtggggttctggaacattggctt
ggttgaccatggccaatgaattgaataagataaaaaccctacatgataccaccaaggtttactcggtgatgttgggcttc
acttgcaactatgatggttatgccggctttgtcgagggtttcagtaccagtaatcaaattcatcctatcgctccggatag
gatacgagtgaagaaagggaagtacggtgctagacagcaagttttccccactggtactacggtttccgaagttaggtcca
attggactttcgtttggaacttcgatgctgctccccctactgggtcaaaggatgctatcactgtgacgaagttttacatc
gccacttcccctttacccggtgtgaagccaccaagtaatttcttggttgttgaggaatga**ctagatggtcacctcggtag**
gaccgagttgccgtacattagaggtaattcctgtgtacgatgtgggtttacaccaccacgtattgtagatcaatacgttt
ggtcgtgtaacttttgttcctatgaacaacatgcatagtggttagtgatgaagtgtttctttcatgaaacctagtgaagg
gtcctccgggatgtgctcagcacctagcctaagctaagtccatatgcccatctttgctgctccggatggatgtttatacc
cgctatggatgcctattactggaatgtaatagatgcctaatactctctctcatggagagagtttagatgcctccaaagga
gatgct

References

1. Zimmermann L, Stephens A, Nam SZ, Rau D, Kübler J, Lozajic M et al. A completely reimplemented MPI bioinformatics toolkit with a new HHpred server at its core. J Mol Biol. 2018;430: 2237-2243. pmid: 29258817

2. Burley SK, Bhikadiya C, Bi C, Bittrich S, Chao H, Chen L et al. RCSB Protein Data Bank (RCSB.org): delivery of experimentally-determined PDB structures alongside one million computed structure models of proteins from artificial intelligence/machine learning. Nucleic Acids Res. 2023;51: D488-D508. pmid: 36420884

3. Mistry J, Chuguransky S, Williams L, Qureshi M, Salazar GA, Sonnhammer EL et al. Pfam: The protein families database in 2021. Nucleic Acids Res. 2021;49: D412-D419. pmid: 33125078

4. Ronquist F, Teslenko M, van der Mark P, Ayres DL, Darling A, Höhna S et al. MrBayes 3.2: efficient Bayesian phylogenetic inference and model choice across a large model space. Syst Biol. 2012;61: 539-542. pmid: 22357727

5. Langmead B, Trapnell C, Pop M, Salzberg SL. Ultrafast and memory-efficient alignment of short DNA sequences to the human genome. Genome Biol. 2009;10: R25. pmid: 19261174

6. Bol JF. Replication of alfamo- and ilarviruses: role of the coat protein. Annu Rev Phytopathol. 2005;43: 39-62. pmid: 16078876

7. Edgar RC. MUSCLE: a multiple sequence alignment method with reduced time and space complexity. BMC Bioinformatics. 2004;5: 113. pmid: 15318951

8. Altschul SF, Gish W, Miller W, Myers EW, Lipman DJ. Basic local alignment search tool. J Mol Biol. 1990;215: 403-410. pmid: 2231712

9. Smirnova E, Firth AE, Miller WA, Scheidecker D, Brault V, Reinbold C et al. Discovery of a small non-AUG-initiated ORF in poleroviruses and luteoviruses that is required for long-distance movement. PLoS Pathog. 2015;11: e1004868. pmid: 25946037

10. Sõmera M, Truve E. The genome organization of lucerne transient streak and turnip rosette sobemoviruses revisited. Arch Virol. 2013;158: 673-678. pmid: 23111554
