# Supplementary material for: A novel ilarvirus protein CP-RT is expressed via stop codon readthrough and suppresses RDR6-dependent RNA silencing
Source: PLoS Pathog. 2024 May 30;20(5):e1012034. doi: 10.1371/journal.ppat.1012034 (PMC11166343; doi:10.1371/journal.ppat.1012034)
Supplement: Attachment — Submitted filename: ResponseToReviewers_1e.pdf [file ppat.1012034.s002.pdf]

Responses to review comments

We thank all three reviewers for their careful assessment of the manuscript, their kind comments, and their constructive suggestions for how to improve the work. A detailed point-by-point response follows below.

Part I - Summary

Reviewer #1: (No Response)

Reviewer #2: The manuscript authored by Lukhovitskaya et al reports a novel readthrough protein (CP-RT) shared by a variety of ilarviruses, and, by using the pathosystem of asparagus virus 2 and Nicotiana benthamiana, discloses its functional role in suppression of host RNA silencing to facilitate viral persistent infection. I appreciate the thorough bioinformatic analysis in defining a putative readthrough domain immediately followed by CP ORF among many subgroups of ilarviruses. The authors adopted a series of approaches, e.g., reverse genetic, wheatgerm extract-based in vitro translation, and ribosome profiling, to demonstrate an actual existence of the readthrough event and its essentiality in supporting viral persistent infection. Along with a hypothesis that RNAi might possibly contribute to the failure of viral persistence / recovery-like phenotype, the authors corroborated a counter-defense role of the readthrough protein likely via targeting the RDR6-involved step in RNAi pathway. Overall, the bioinformatic analysis and experimental design and methods are appropriate, and the obtained results support the conclusions. The novel findings greatly enhance our understanding on the fundamental aspects of ilarvirus biology. However, I still have several comments that need to be properly addressed before its publication, see my concerns in Part II and III.

Reviewer #3: This manuscript presents an extensive investigation into a new protein variant of ilarviruses. These viral genomes are compact and maximizing use of its capacity is assumed. In this study, a novel short extension of the CP by read-through is bioinformatically predicted and extensively proved both to exist in native infections as well as contributing to infection by RNA silencing mechanisms enabling the virus to persist in meristems. Furthermore, demonstrating that the same RT domain from another ilarvirus can complement broadens the context nicely. The work is truly substantial, methodology is top-level and the resulting claims are fully justified. In fact, the only “weakness” I can identify is that the paper is so heavy, i.e. almost 100 pages supplementary. There are some typos and small things in the text, but the lack of line numbering made it too tedious for me to point out such minor faults.

Altogether, I see no need for additional experimentation with the confession that I have limited detailed expertise to evaluate technicalities regarding the sequencing analyses. I think the study has taken the best methods to evidence the predicted RT and its functional meaning.

One interesting point for biological relevance would be whether the presence of the RT controls vertical transmission of the virus.

This is indeed a good suggestion; thank you. Our model suggests that CP-RT, as a suppressor of RNAi, changes the pattern of meristem invasion by AV2 which would be expected to subsequently affect vertical transmission. Since AV2-2st is unable to infect *N. benthamiana* persistently and is cleared from the upper parts of the plant by 14 dpi, it is an unsuitable model to study vertical transmission. As a subject of future work, it would be interesting to compare AV2 and AV2-2st infection in a different host. AV2 has been shown to infect at least twenty-seven species of plants in seven families so we can hope to find a host where the effect of CP-RT knockout would be milder, and it may be possible to separate changes in virus accumulation in seeds from general clearance of the virus.

Part II – Major Issues: Key Experiments Required for Acceptance

Reviewer #1: (No Response)

Reviewer #2: Amongst different subgroups of ilarviruses, the CP stop codon and surrounding motif for a putative readthrough event is present. In line with this, the "CP-RT" protein was detected by in vitro translation assay, immunoblotting analysis with an antibody against AV2 CP or Myc/HA tag fused at the C-terminus of RT. I believe that the discovery of the readthrough event is a breakthrough in our understanding the fundamental biology of ilarvirus. To further consolidate the conclusion, I suggest following experiments for considerations: 1. Purification of Myc-tagged CP-RT for mass spectrometry (MS) analysis, and thus, a portion of the peptide sequences from MS analysis are expected to correspond with RT sequence.

Thank you. Indeed this is a great suggestion which we did also consider during our work.

In an effort to identify its cellular binding partners, we attempted to purify transiently overexpressed CP-RT (stop codon replaced with UGG) by immunoprecipitation with anti-CP antibodies. Unfortunately, despite the addition of protease inhibitors and other precautions, we were not able to detect CP-RT even in the input fraction after tissue lysis and preclearing of lysates (Fig. A). When samples were prepared immediately after collection of leaf material, we could detect overexpressed CP-RT (stop codon replaced with UGG) with anti-CP antibodies but in much lower quantity compared to CP, even though both CP and CP-RT proteins were expressed from the same vector (Fig. B). These observations suggest inherent instability of CP-RT. Although we can detect C-terminally Myc- or HA-tagged CP-RT by WB, the tag attenuates AV2 and samples for IP could be collected only during the early stages of infection (Fig. 4D, Fig. S9) when the virus titre is still low.

As a result of the instability issues, besides the very low efficiency of readthrough in AV2 (Fig. 5D), we do not think it will be possible for us to obtain sufficient quantity of CP-RT by immunoprecipitation from infected plants for MS analysis. Thus, to avoid lengthy delays, we are resubmitting the manuscript without this analysis. However, we can still try to do this experiment if the reviewer thinks it is essential.

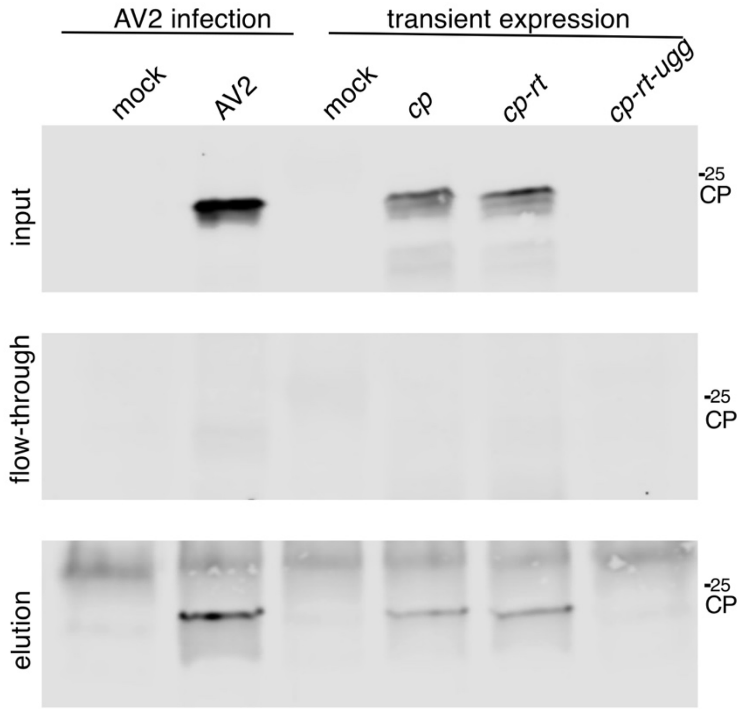

Figure A. Immunoprecipitation of CP and CP-RT with protein G pre-bound anti-CP antibodies. Samples were collected at 7 dpi from upper non-inoculated leaves of AV2-infected plants and at 4 dpi from agroinfiltrated leaves transiently expressing *cp, cp-rt* and *cp-rt-ugg.* Immunoprecipitates were subjected to western blot analysis using the same antibodies. The position of CP and sizes of molecular weight markers are indicated on the right.

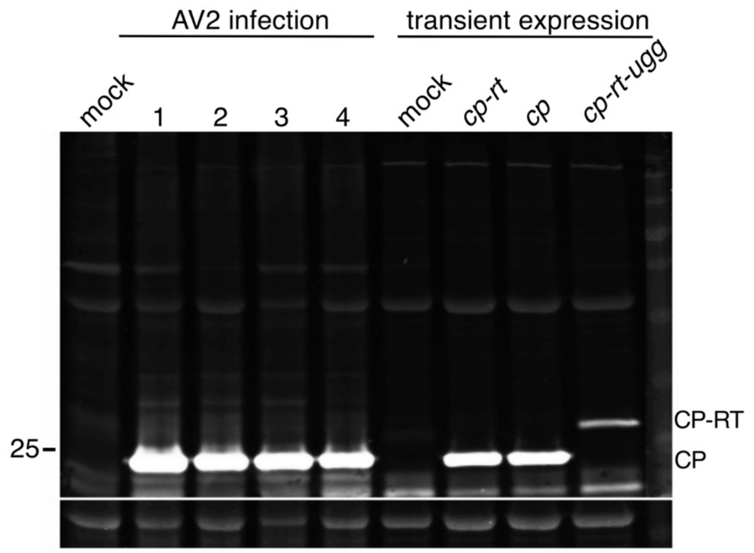

Figure B. Detection of CP and CP-RT by western blot in plants infected with AV2 and plants agroinfiltrated with *cp-rt, cp* and *cp-rt-ugg.* Samples were collected at 7 dpi from upper non-inoculated leaves of infected plants and at 4 dpi from agroinfiltrated leaves. Positions of CP and CP-RT are indicated on the right. Sizes of molecular weight markers are indicated on the left, and Ponceau red staining (lower panels) was used as a loading control.

2. Based on the authors' analysis, the existence of RT domain is commonly shared by a large number of ilarviruses. It should be a real situation in ilarviral infection in

nature. The experimental data about the presence of CP-RT is lacking. Performing immunoblotting analysis of AV2 CP-RT using CP antibody is suggested.

We did indeed try to do this (Fig. 3G). Unfortunately, due to the very low readthrough efficiency (Fig. 4B, Fig. 5D), besides its probable instability (see previous point), we were not able to detect CP-RT in AV2-infected plants using anti-CP antibodies (Fig. 3G, Fig. B). Thus, our experimental evidence for expression of CP-RT during AV2 infection is the ribosome profiling of infected plants (Fig. 5D) and detection of the C-terminally Myc- or HA-tagged CP-RT in plants infected with AV2-myc (Fig. 4D, 4E) or AV2-HA (Fig. S9). This of course is complemented by the comparative genomic and knockout mutant phenotype approaches.

Reviewer #3: None needed for acceptance, vertical transmission regulation by RT appears as a relevant biological possibility.

See response above in Part I.

Part III – Minor Issues: Editorial and Data Presentation Modifications

Reviewer #1: (No Response)

Reviewer #2: 1. As shown in Figure 2, for many ilarviruses, they encode both 2b and a potential CP-RT to function in counteracting host RNA silencing. The authors should discuss the functional differences between them in detail. Some ilarviruses have a single 2b (Apple ilarvirus 2), and some contain neither 2b nor CP-RT (Apple mosaic virus - 6a, Blueberry shock virus, etc.). Why are viruses in the same genus so different in viral suppressors of RNA silencing?

This is of course an excellent question, and one that we have asked ourselves also. There are of course a number of examples of plant RNA viruses containing multiple VSRs – for example potyvirus HC-Pro and VPg (PMID 27795417) and citrus tristeza closterovirus p25, p20 and p23 (PMID 15505219) (other references given near the end of the 2nd-last paragraph of the manuscript Introduction and in the 4th-last paragraph of the manuscript Discussion). Variations within a genus in both protein complement and proteins with VSR activity are also known – e.g. P1-PISPO, a protein restricted to a small subgroup of potyviruses, has VSR activity and indeed in these potyviruses P1 does also (PMID 30374036). In many RNA viruses, proteins with one conserved function also take on additional, often less conserved functions, e.g. the turnip crinkle virus capsid protein also acts as a VSR (PMID 12477856). These auxiliary functions often tend to be more phylogenetically restricted than the original function, but evolve and are retained because they still increase virus fitness.

It may be that 2b and RT were not present in the last common ancestor of ilarviruses, thus explaining why – even though advantageous – they are not present in all members of the genus. It is possible that one of the other viral proteins (1a, 2a, MP) also has VSR activity – even if only weakly – and was perhaps the main VSR of ancestral ilarviruses; this activity would have been supplemented and possibly supplanted in the ilarviruses that evolved 2b and/or RT. We have added to the 3rd-

last paragraph of the Discussion the text "It may be that the 2b and RT ORFs were both not present in the last common ancestor of ilarviruses, thus explaining why – even though advantageous – they are not present in all members of the genus."

Regarding a detailed discussion of the functional differences between 2b and RT: Unfortunately the only work, so far as we can detemine, on ilarvirus 2b is the Shimura et al 2013 paper. This establishes 2b as a suppressor of systemic silencing, but the mechanisms were not identified. Thus we cannot really give any more detail on the functional differences between 2b and RT beyond what is already in the manuscript.

2. It is curious that most CP-RT proteins in ilarvirus consist of zinc finger motif, and the motif is associated with RSS activity and viral persistent infection. The zinc finger in viral suppressors of RNA silencing is commonly shared by other viruses in different genera?

It is indeed true that some other VSRs also have Zn finger motifs. For example, a Zn finger is a common feature of VSRs belonging to the roughly defined group of plant RNA virus cysteine rich proteins (CRPs). This group was discussed by Koonin et al. in 1991 (PMID 1994589) and originally consisted of small proteins encoded by a 3′ ORF of the respective genomic RNAs of hordei-, furo-, tobra- and carlaviruses. Functional importance of the Zn finger for VSR activity was demonstrated for some CRPs, e.g. BSBMV p14 (Chiba et al., 2013, PMID 23013437) and (probably) BSMV γb (Bragg et al., 2004, PMID 20565621). Interestingly, the BSMV γb Zn finger is involved in RNA binding whereas the BSBMV p14 Zn finger is involved in protein dimerization, stability and nucleolar targeting. Considering the nuclear and cytoplasmic localisation of CP-RT (Fig. C) there are multiple potential roles for the CP-RT Zn finger. We are continuing to investigate this aspect of CP-RT; however we feel that this will benefit from analysis of CP-RTs from multiple ilarvirus species (not just AV2) and is beyond the scope of the current manuscript.

We have added to the Discussion the text "The mechanistic role of the zinc finger in VSR activity remains undetermined though, interestingly, a number of other plant RNA virus VSRs also contain zinc fingers that in some cases have been shown to be involved in the VSR activity (Bragg et al., 2004; Chiba et al., 2013)."

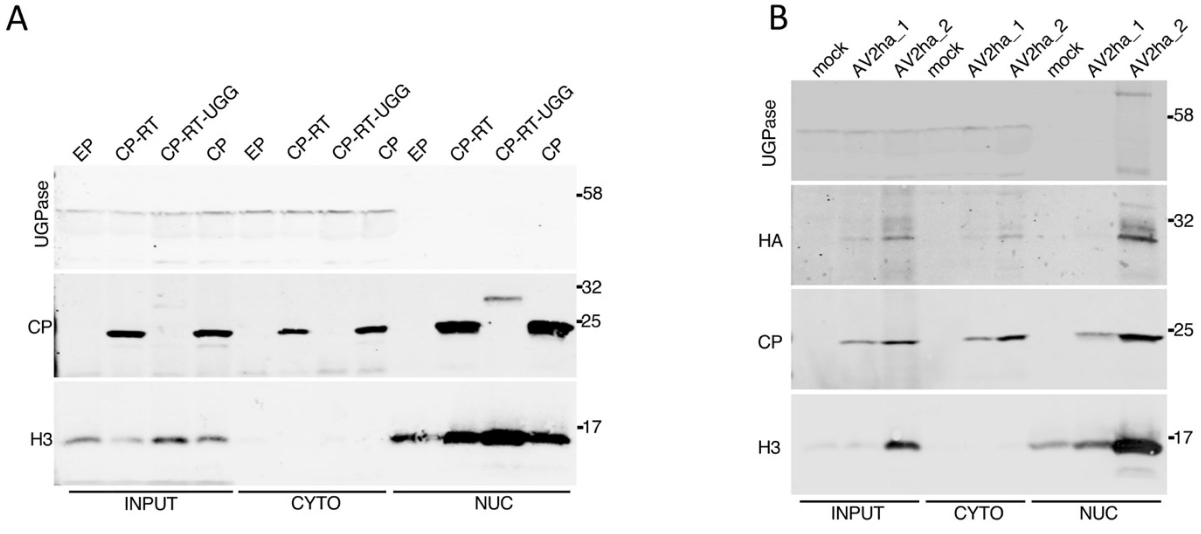

Figure C. Presence of CP-RT and CP in the nuclear P10 and cytoplasmic S30 fractions. Subcellular fractionation of *N. benthamiana* agroinfiltrated with *cp-rt, cp* or *cp-rt-ugg.* Samples were collected at 3 dpi (A). Subcellular fractionation of upper non-inoculated leaves of *N. benthamiana* infected with AV2-HA. Samples were collected at 4 dpi (B). Fractions were analyzed by western blot against CP, histone H3 as a nuclear marker, and UGPase as a cytoplasmic marker (A and B) and against HA tag (B). Positions of proteins are indicated on the left. Sizes of molecular weight markers are indicated on the right.

It is whether or not CP-RTs serve as transcription factors in regulating the expression of RDR6's expression to affect RNAi pathway in an indirect manner? Does the CP-RTs have a nucleus-localized signal?

We tested CP-RT from the 29 cluster reference sequences with an RT domain with various nuclear localisation and/or nuclear localisation signal (NLS) prediction software, including WoLF PSORT, NLStradamus, cNLS Mapper and NucPred. However we found only very occasional predictions of nuclear localisation / nuclear localisation signals, and not in the AV2 sequence. However it should be noted that such predictors are not particularly accurate and there may be types of NLS that are not yet characterised.

Using qRT-PCR, we analysed *rdr6* mRNA accumulation in the apical parts of plants infected with AV2, AV2-2st or AV2-mutZF. However, we did not find any significant differences (Fig. D). Thus it is unlikely that CP-RT directly regulates *rdr6* expression.

We also obtained RNA-seq data for plants that were infected with AV2 or AV2-2st virus or mock infected, with the hope of performing a differential expression analysis to look for CP-RT-mediated transcriptional regulation. However, based on quality control analysis (PCA, clustering, etc) we were concerned that the variation between replicates was not small enough to allow a robust differential expression analysis. (These RNA-seq data appear in the manuscript but, due to this issue, they were used only for analysing virus transcript expression ratios and the stability of the virus infectious clone, where data could be shown separately for each of the three repeats; Fig. S8).

By colocalization with RFP-tagged fibrillarin, a marker for nucleolar/cajal bodies, we found that, when transiently overexpressed, a CP-RT with a C-terminal GFP fusion (CP-RT-UGG-GFP) localises at least partly to the nucleus and nucleolus (Fig. E). Using tissue fractionation, transiently overexpressed CP-RT-UGG was detected only in the nuclear fraction whereas CP-RT-HA expressed upon infection with AV2-HA virus was detected in both nuclear and cytoplasmic fractions (Fig. C). At present, we do not know which fraction of CP-RT – nuclear or cytoplasmic (or both) – is responsible for its VSR activity.

However these data are preliminary so, while we don't think that CP-RT regulates *rdr6* mRNA transcription, and this is not a priority for our future work, we don't think that we can include these data in the current manuscript.

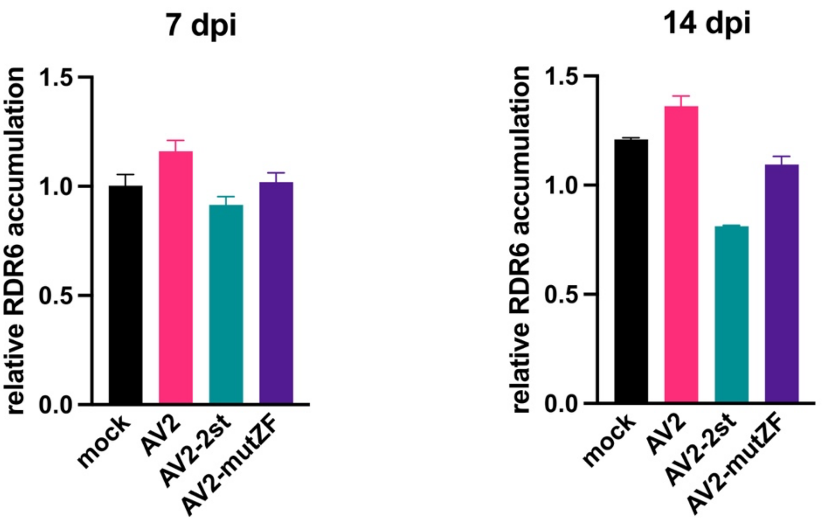

Figure D. RT-qPCR analysis of *rdr6* accumulation in apical tissues of infected plants. Wild type *N. benthamiana* plants were infected with AV2, AV2-2st or AV2-mutZF, and samples were collected at 7 and 14 dpi. Shoot apices of five infected plants were pooled together for RNA extraction. *Nb ACT-b* (GI:380505031) was used as a reference gene. Values are expressed in arbitrary units and represent the mean +/− SEM of three technical replicates.

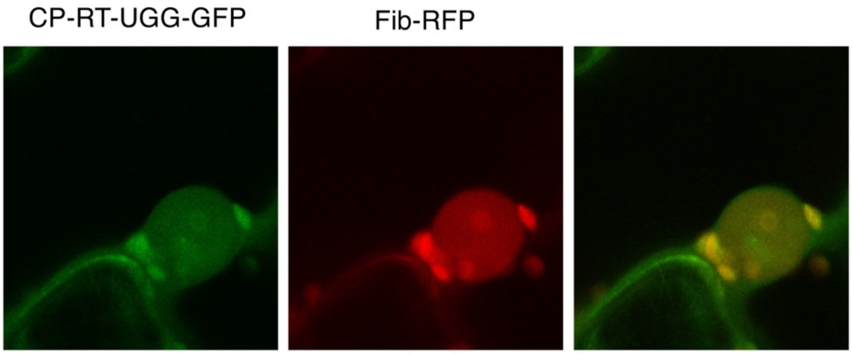

Figure E. Co-localization of CP-RT-UGG-GFP with the nuclear/nucleolar/cajal bodies marker mRFP-Fibrillarin transiently co-expressed in *N. benthamiana* epidermal cells at 3 dpi. Confocal microscopy images showing localization of GFP fusions (left), mRFP-Fib (middle) and the superposition of these two images (right).

3. In Page 12 “we were unable to see a clear unambiguous effect of …………on gfp siRNA accumulation (Figure 7D)”. The suppression of RNA silencing is usually accompanied with a less accumulated vsiRNA? Please clarify this point.

Yes, indeed, this result was unexpected. To clarify this, we have changed "However, whereas ..., we were unable to see ..." to "Unexpectedly, whereas ..., we were unable to see ...".

One of the possible explanations for the lack of effect of CP-RT on *gpf* siRNAs could be the weak VSR activity of CP-RT in transient silencing suppression assays. Co-expression with *cp-rt-ugg* does significantly increase *gfp* mRNA accumulation (Fig. 7C), but this effect is much less pronounced than for p19, where we can also observe a negative effect on siRNA accumulation. When we compare by deep sequencing of siRNAs the levels of vsiRNAs in plants infected with AV2 or AV2-2st, we do see an obvious increase in vsiRNA accumulation in the absence of CP-RT.

However, it is difficult to correlate transient silencing suppression assays with virus infection in terms of dynamics, expression levels, etc. Deep sequencing of siRNAs is also a much more sensitive and quantitative approach than northern blot so it is possible that there is a difference in *gfp* siRNA accumulation but that the northern blot analysis was not sensitive enough to robustly detect it.

Reviewer #3: The text is somewhat heavy, especially the cross-referencing to the table in the first results section. It makes it hard to grasp at a general level so I therefore suggest to simplify and clearly lift out conclusions to the extent possible without compromising scientific quality of course.

We do appreciate the reviewer's concern and agree that the text is rather lengthy and therefore can be heavy going. However we are reluctant to embark on modifications to the style. We have aimed to adopt an approach that is thorough and complete while still keeping the text as concise as possible. We feel that changing the comparative genomics section to make it lighter will also result in the loss of important detail. Given that the manuscript describes a new protein, we feel that a complete description of the comparative genomic evidence is useful.
